# A reproducible three-dimensional model of human brain tissue to investigate physiological and disease-associated microglia phenotypes

**DOI:** 10.1038/s41593-026-02367-0

**Published:** 2026-08-04

**Authors:** Julien Klimmt, Carolina Cardoso Gonçalves, Jessica Valentina Montgomery, Stephan A. Müller, Merle Bublitz, Severin Filser, Lars Paeger, Brigitte Nuscher, Angelika Dannert, Sigrun Roeber, Veronica Pravata, Martina Schifferer, Joshua J. Shrouder, Nathalie Schulz, Judit González-Gallego, Silvia Cappello, Thomas Misgeld, Nikolaus Plesnila, Eduardo Beltrán, Jochen Herms, Elena De Domenico, Marc D. Beyer, Joachim L. Schultze, Christian Haass, Stefan F. Lichtenthaler, Caterina Carraro, Dominik Paquet

**Affiliations:** 1 Institute for Stroke and Dementia Research (ISD), LMU University Hospital, LMU Medizin, LMU Munich, Munich, Germany; 2 Graduate School of Systemic Neuroscience (GSN), LMU Munich, Munich, Germany; 3 Systems Medicine, Deutsches Zentrum für Neurodegenerative Erkrankungen (DZNE) e.V., Bonn, Germany; 4 German Center for Neurodegenerative Diseases (DZNE) Munich, Munich, Germany; 5 Neuroproteomics, School of Medicine and Health, TUM University Hospital, Technical University of Munich, Munich, Germany; 6 Institute of Neuropathology, LMU Medizin, LMU Munich, Munich, Germany; 7 Metabolic Biochemistry, Biomedical Center (BMC), Faculty of Medicine, LMU Munich, Munich, Germany; 8 Chair of Physiological Genomics, Biomedical Center (BMC), LMU Medizin, LMU Munich, Munich, Germany; 9 Munich Cluster for Systems Neurology (SyNergy), Munich, Germany; 10 Max-Planck-Institute of Psychiatry, Munich, Germany; 11 Institute of Neuronal Cell Biology, Technical University of Munich, Munich, Germany; 12 Institute of Clinical Neuroimmunology, LMU University Hospital, LMU Medizin, LMU Munich, Munich, Germany; 13 Biomedical Center (BMC), LMU Medizin, LMU Munich, Munich, Germany; 14 PRECISE Platform for Genomics and Epigenomics, Deutsches Zentrum für Neurodegenerative Erkrankungen (DZNE) e.V. and University of Bonn and West German Genome Center, Bonn, Germany; 15 Immunogenomics & Neurodegeneration, Deutsches Zentrum für Neurodegenerative Erkrankungen (DZNE) e.V., Bonn, Germany; 16 Genomics and Immunoregulation, Life & Medical Sciences (LIMES) Institute, University of Bonn, Bonn, Germany

**Keywords:** Neuroimmunology, Induced pluripotent stem cells, Alzheimer's disease, Neurodegeneration, Experimental models of disease

## Abstract

Stem-cell-based in vitro models offer promising potential to elucidate human brain cell functions and interactions, but limitations in reproducibility, maturation and cell-type diversity persist. Especially, prolonged incorporation of mature microglia and studies of neuroinflammation have proven challenging. Here, we developed a human induced pluripotent stem cell-based three-dimensional cortical brain tissue model (3BTM) containing neurons, astrocytes and microglia with high reproducibility, maturity and viability. 3BTMs show morphological, functional and proteomic maturation of all cell types, leading to high similarity to their in vivo counterparts. Incorporated microglia survive for over 6 months and display mature morphology, functions and gene expression. Importantly, when engineered to model Alzheimer’s disease pathology, 3BTMs recapitulate key disease hallmarks, including amyloid deposition, increased phospho-tau levels and neuroinflammation, with microglia shifting their transcriptional landscape to disease-relevant signatures. Treatment of Alzheimer’s disease 3BTMs with anti-Aβ immunotherapy cleared deposits and largely reversed disease signatures in glia. Together, our microglia-containing model provides a platform for studying physiological and pathological states of human brain tissue.

## Abstract (web summary)

Klimmt, Cardoso Gonçalves et al. developed a reproducible human three-dimensional brain tissue model with neurons, astrocytes and microglia, replicating in-vivo-like maturation and enabling the study of Alzheimer’s disease-relevant perturbations and drug responses.

## Main

Investigating physiological and disease-related interactions of human neurons and glia requires experimentally accessible and well-characterized model systems. Recent progress in stem cell research has allowed generating multiple brain cell types from induced pluripotent stem cells (iPSCs), studying their interactions in co-culture and investigating pathomechanisms in disease-relevant cell types. Although two-dimensional (2D) models containing neurons and glia co-cultured in a dish allow simple model generation, they often lack physiological morphology, maturation, homeostatic behaviors and intercellular crosstalk found in tissues^1–3^. Three-dimensional (3D) models better recapitulate cell–cell and cell–matrix interactions in a more physiological environment and enable investigation of extracellular pathologies, such as accumulation of amyloid-β (Aβ) in Alzheimer’s disease (AD), besides intracellular events such as tau phosphorylation.

Current human iPSC-based 3D models include hydrogel-based systems, spheroid-like cultures and neural organoids (NOs). Hydrogel-based systems embed iPSCs^2^ or neural precursor cells (NPCs)^4^ into synthetic or cell-derived matrices such as Matrigel but cannot yet reproduce the brain’s unique extracellular matrix (ECM), limiting the establishment of physiological cell states, functions and disease responses^5^. Spheroid cultures, generated by the aggregation of NPCs^6^, or NPCs and astrocytes^7^, and their maturation in 3D, show good reproducibility in microwells but lack stable incorporation of mature and homeostatic microglia (MG), the brain-resident immune cells, and long-term in vitro maintenance. This limits replication of tissue characteristics, including formation and maturation of the ECM and establishment of mature cell morphologies, functions and interactions. NOs develop in 3D directly from stem cells and uniquely model aspects of brain development^8^, giving important insights into human-specific mechanisms. However, NOs in vitro show limited suitability to study MGs, which have major roles in the maintenance of homeostasis and neurodegenerative diseases^9^. MGs arise from the mesoderm; therefore, they are not observed in guided, neuroectodermal organoids, whereas unguided protocols can yield few MGs^10^. Exogenously added iPSC-derived microglia (iMG), promote organoid maturation^11^, but rapidly decline in numbers and usually disappear within a few weeks^12,13^. Furthermore, these iMGs lack mature and homeostatic signatures or morphologies in vitro^11,12,14–16^. Such maturation was only achieved after xenotransplantation into the mouse brain^12^, confirming the ability of iMGs to mature in iPSC-derived models; however, these models are highly complex and low throughput, limiting their suitability for basic and translational research.

Therefore, we hypothesized that mature, homeostatic MGs require extrinsic factors not found in current in vitro models, including a postmitotic, mature brain-parenchyma-like environment, low cell death and associated stress, and presence of mature astrocytes. The inapt environment provided by current models, such as the lack of mature astrocytes in trackable time frames^17^ and the proliferative state, associated large-scale growth and subsequent necrotic core formation^18^ present in NOs, may thus prevent formation of mature iMG phenotypes. Furthermore, the artificial, environment-related activation observed in non-homeostatic iMG in vitro may impair studies on microglial responses to disease, such as AD.

To overcome the drawbacks of current models, we developed a highly reproducible, iPSC-derived 3D cortical tissue model with high viability for many months in a postmitotic state without necrotic core formation, and efficient and reproducible incorporation of cortical neurons (iNEs), astrocytes (iAS) and iMGs across multiple iPSC lines. Co-cultured cells form a 3D structure with brain-like ECM formation, maturation of cell types on a morphological, functional and protein expression level, and adoption of a mature iMG state with homeostatic marker expression and ramified morphology. Using CRISPR–Cas9 knock-in of synergistic *APP* mutations, we established an AD model replicating disease hallmarks, including amyloid deposition, increased phospho-tau (p-tau) levels, neuroinflammation and disease-associated microglial signatures. We showed formation of Aβ plaque-like structures upon addition of exogenous Aβ and confirmed suitability of the model to test anti-amyloid antibodies and their effect on brain cell types. Taken together, we developed a fully iPSC-based 3D in vitro brain tissue model that allows studying physiological and pathological phenotypes of mature iNEs, iAS and iMGs in a reproducible, human-tissue-like environment.

## Results

### Generation of a reproducible human 3D brain tissue model containing homeostatic MG

**Fig. 1:**
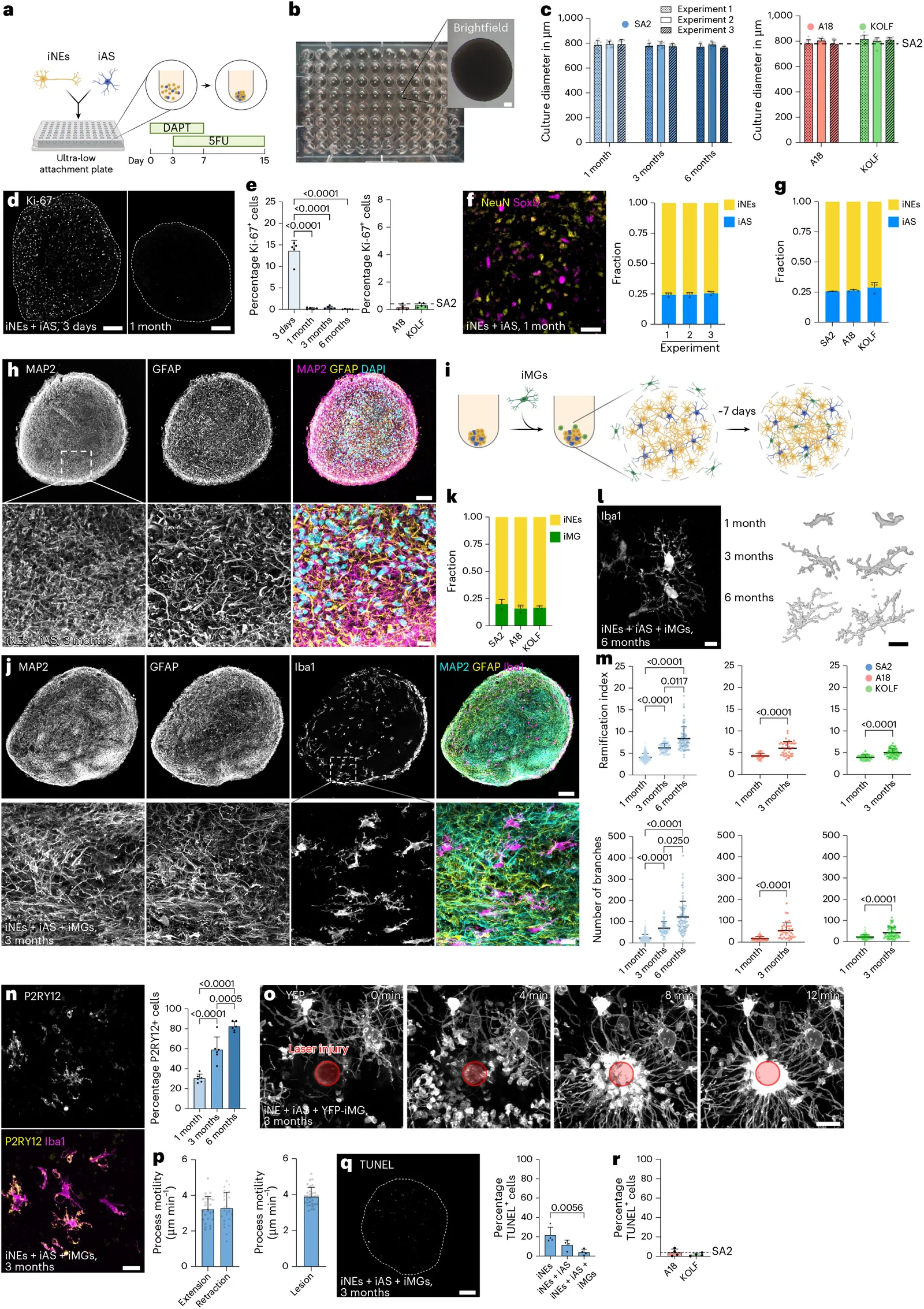
Generation of a reproducible human 3D brain tissue model containing homeostatic MGs. **a**, Experimental approach to generate 3BTMs. **b**, Overview of a 96-well plate with 3BTMs at 1 month of age. Right: Brightfield image at 1 month. **c**, Quantification of culture diameters at 1, 3 and 6 months in line SA2 (left), and at 3 months in lines A18 and KOLF (right, the dashed line indicates the average diameter in line SA2 at 3 months) (*n* = 9 (A18 experiment 1), *n* = 10 (remaining), *n* = 11 (SA2 1 month experiment 1, KOLF experiment 2) 3BTMs (technical replicates) per experiment and time point, 3 experiments (biological replicate) shown). **d**, Immunofluorescence (IF) staining of Ki-67^+^ cells in SA2 3BTMs at 3 days and 1 month. **e**, Quantification of Ki-67^+^ cells in line SA2 over time (left) and in lines A18 and KOLF at 3 months (right, the dashed line indicates the value in line SA2 at 3 months) (*n* = 5 experiments (biological replicate) with 1–2 3BTMs each, one-way analysis of variance (ANOVA) with Tukey’s multiple comparisons test). **f**, IF staining showing NeuN^+^ iNEs and Sox9^+^ iAS in SA2 3BTMs at 1 month, quantified across experiments (right) (*n* = 3 3BTMs (technical replicate) per experiment and time point, three experiments (biological replicates) shown). **g**, Quantification of NeuN:Sox9 ratio at 3 months (*n* = 3 experiments (biological replicates) with 2–3 3BTMs per line). **h**, IF staining of iNE+iAS 3BTM at 3 months for MAP2 (iNEs) and GFAP (iAS). **i**, Experimental approach to generate 3BTMs containing iNEs+iAS+iMGs. **j**, IF staining of 3-month-old iNE+iAS+iMG 3BTM for iNEs, iAS and iMGs (Iba1), scale bar: 100 µm. **k**, Quantification of fractions of iNEs and iMGs (based on NeuN and PU.1 staining) at 3 months (*n* = 3 experiments (biological replicates) with 2–3 3BTMs per line). **l**, Left: IF staining of iMGs at 6 months. Right: 3D reconstructions of MGs in 3BTMs at 1, 3 and 6 months. **m**, Quantification of MG morphology over time measured using the ramification index (top) and number of branches (bottom) per cell (SA2 1 month: *n* = 192, 3 months: *n* = 61, 6 months: *n* = 81; A18 1 month: *n* = 105, 3 months: *n* = 91; KOLF 1 month: *n* = 132, 3 months: *n* = 119 cells (biological replicates), Kruskal–Wallis tests with Dunn’s multiple comparisons test). **n**, IF staining at 3 months for P2RY12 in line SA2 (left). Right: Quantification of P2RY12^+^ cells over time (*n* = 6 experiments (biological replicates), two 3BTMs per experiment and time point; one-way ANOVA with Tukey’s multiple comparisons test). **o**, Two-photon live-cell imaging of 3BTM at 3 months in line SA2 with iMGs expressing membrane eYFP, showing reaction to a focal laser injury (red circle). **p**, Quantification of iMG process motility under baseline conditions (left, *n* = 25 tracks (=measured process) per experiment and direction, taken from three experiments (biological replicates) with 8–9 tracks each) and after focal laser injury (right, *n* = 31 tracks from three experiments (biological replicates) with 10–11 tracks each). **q**, IF staining showing levels of cell death by TUNEL in SA2 3BTM at 3 months. Right: Quantification showing comparison of cell-type combinations (*n* = 4 experiments (biological replicates) with two 3BTMs per cell-type combination; one-way ANOVA with Tukey’s multiple comparisons test). **r**, Quantification of TUNEL^+^ cells in 3BTMs at 3 months in the A18 and KOLF cell lines (*n* = 4 experiments (biological replicates) with two 3BTMs per line; one-way ANOVA with Tukey’s multiple comparisons test; the dashed line indicates the value in the SA2 line at 3 months). Data are presented as the mean + s.d. **b**,**e**,**h**,**j**,**q**, Scale bar: 100 µm. **h**,**j**, Zoom-in scale bar: 20 µm. **f**, Scale bar: 30 µm. **l**, Scale bar: 10 µm. **n**,**o**, Scale bar: 20 µm. Panels **a,i** created in BioRender; https://biorender.com/h8a1o1y (2026). Source data

**Extended Data Fig. 1:**
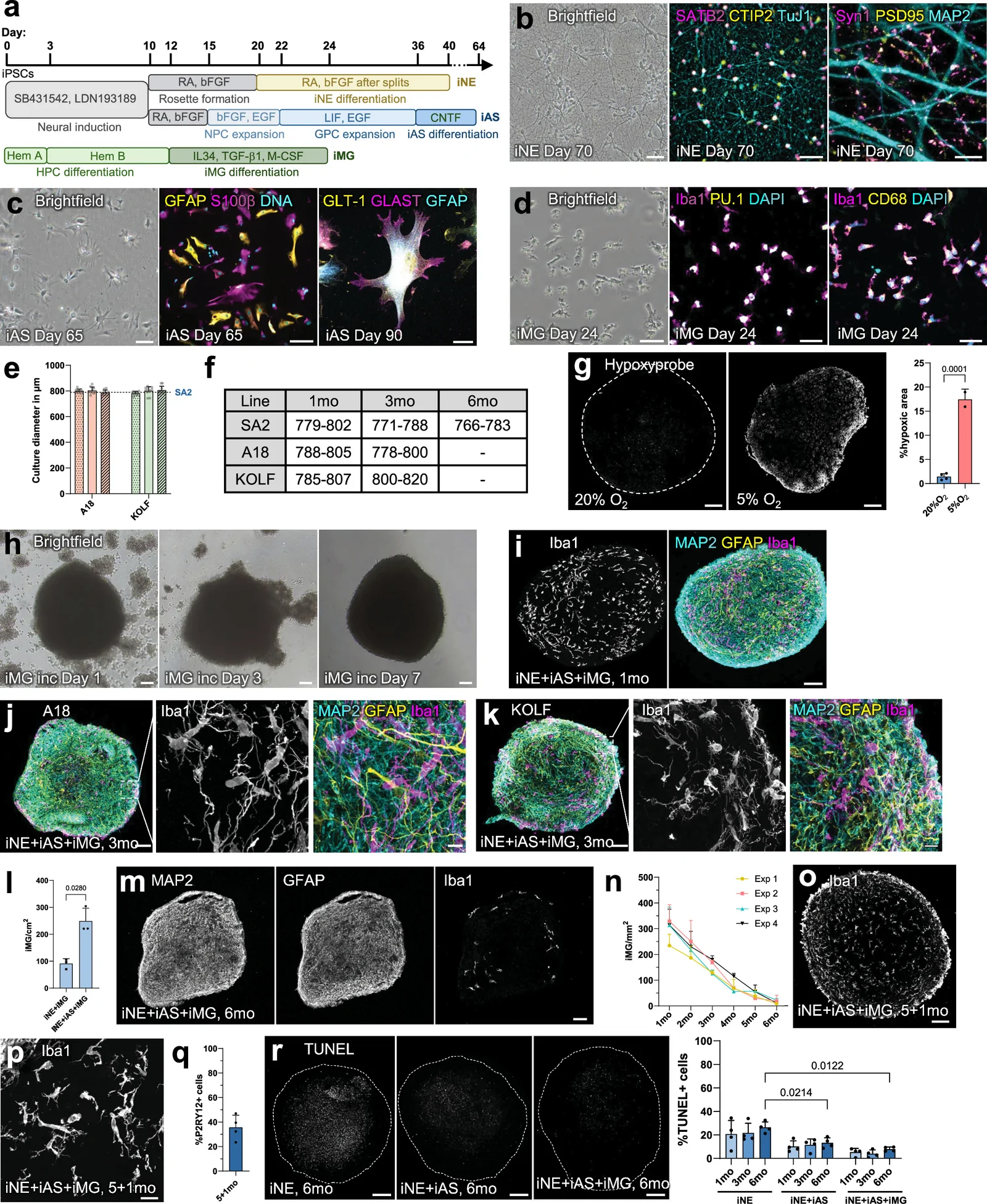
Differentiation of iPSC-derived brain cell types and general characterization of 3BTMs. (**A**) Overview of protocols to differentiate iPSCs into cortical neurons (iNEs), astrocytes (iAS), and microglia (iMGs). (**B**) iNEs in 2D monoculture on Day 70 after iPSC stage. Left: Brightfield image, scale bar 50 µm. Middle: IF staining showing expression of cortical markers SATB2 and CTIP2 and beta-III tubulin (TuJ1), scale bar 50 µm. Right: IF staining showing MAP2-positive neurites and colocalization of presynaptic Synapsin-1 (Syn1) and postsynaptic PSD95, scale bar 5 µm. (**C**) iAS in 2D monoculture on Day 65. Left: Brightfield image, scale bar 100 µm. Middle: IF stainings for S100beta and GFAP (scale bar 100 µm). Right: IF stainings for GLT-1 and GLAST (scale bars 20 µm). (**D**) iMG in 2D monoculture on Day 24. Left: Brightfield image, scale bar 50 µm. Middle: IF stainings for Iba1 and PU.1, scale bar 30 µm. Right: IF stainings for Iba1 and CD68, scale bar 30 µm. (**E**) Quantification of culture diameters at 1mo in lines A18 and KOLF, dashed line indicates average diameter in line SA2 (n = 10 3BTMs (tech. repl.) per experiment and timepoint, 3 experiments per line (biol. repl.) shown. (**F**) Table showing 95% confidence intervals of culture diameter in all lines over time. (**G**) IF stainings in 3-month-old iNE+iAS SA2 3BTM using Hypoxyprobe^71^, under normal (20% O_2_) and hypoxic conditions (5% O_2_, positive control). Quantification on the right (n = 2 (5% O_2_) and n = 4 (20% O_2_) experiments (biol. repl.) with two 3BTMs each (tech. repl.)), scale bars 100 µm. (**H**) Brightfield images showing migration of iMGs into 3BTM in line SA2, scale bars 100 µm. (**I**) IF staining of iNE+iAS+iMG SA2 3BTM at 1mo for Iba1, MAP2 and GFAP, scale bars 100 µm. (**J**) IF staining of a 3mo A18 3BTM, scale bar 100 µm. Zoom-in on the right, scale bar 10 µm. (**K**) IF staining of a 3mo KOLF 3BTM, scale bar 50 µm. Zoom-in on the right, scale bar 10 µm. (**L**) Quantification of iMG numbers in SA2 3BTM without and with iAS at 1mo (n = 3 experiments (biol. repl.) with two 3BTMs each, paired *t*-test). (**M**) IF staining of SA2 3BTM at 6mo showing the different cell types, scale bar 100 µm. (**N**) Quantification of iMG numbers in SA2 3BTMs over time (n = 4 experiments (biol. repl.) with two 3BTMs each, represented by colored lines). (**O**) IF staining of 6mo SA2 3BTM repopulated with iMG at 5mo, scale bar 100 µm. (**P**) Close-up of IF staining as in (**O**), scale bar 20 µm. (**Q**) Quantification of %P2RY12-positive cells at 6mo in SA2 3BTM repopulated with iMG at 5 months of age (n = 4 experiments (biol. repl.) with two 3BTMs each). (**R**) IF TUNEL stainings of 3BTMs containing iNE, iNE+iAS, or iNE+iAS+iMG at 6mo, scale bars 100 µm. Quantification on the right (n = 4 experiments (biol. repl.) with two 3BTMs each, Repeated measures two-way ANOVA with Tukey’s multiple comparisons test comparing all cell type combinations between each timepoint, data for 3mo iNE+iAS+iMG also shown in Fig. 1r). Data are presented as mean + SD. Source data

We hypothesized that generation of a reproducible and controllable 3D cortical brain tissue model (3BTM) can be achieved by aggregating brain cells that adopt a stable fate but are plastic enough to allow tissue integration (Fig. 1a), to reduce heterogeneity originating from 3D differentiation of stem or progenitor cells. To test this approach, we optimized small-molecule-based 2D differentiation protocols to separately obtain homogeneous and pure iNE^19^, iAS^20^ and iMG^21^ cultures (Extended Data Fig. 1a). We confirmed typical cell morphologies, marker expression and purity using immunostaining (Extended Data Fig. 1b–d). To generate 3BTMs, the differentiated cells were self-aggregated into 3D assemblies in ultra-low attachment plates (Fig. 1a). We avoided artificial ECMs, such as Matrigel, to minimize variability of exogenous cues and increase reproducibility and simplicity. To ensure reproducibility, we validated 3BTM generation using iNEs, iAS and iMGs differentiated from the iPSC line 7889SA2 (‘SA2’, male) and confirmed key results in two additional lines, that is, A18944 (‘A18’, female) and KOLF2.1J (‘KOLF’, male, proposed as the ‘reference iPSC line’ for neuroscience research^22^). We combined iNEs and iAS in a 3:1 ratio, mimicking the human cortex^23^, and confirmed aggregation into uniform, sphere-like structures of about 800-µm diameter (Fig. 1b). To avoid growth and necrotic core formation and reach a postmitotic state rapidly after aggregation, cultures were treated with DAPT and 5-fluorouracil (5FU)^19^ (Fig. 1a). Combining optimized differentiation protocols, aggregation time points and compound treatments yielded 3BTMs with largely stable diameters over several months in all three lines (Fig. 1c and Extended Data Fig. 1e,f). Further confirming the efficacy of the compound treatments, proliferating cells were abundant on day 3 after culture generation, but almost absent at all later time points (Fig. 1d,e). Additionally, cultures maintained the initial iNE:iAS ratio of 3:1 (that is, 75% iNEs, 25% iAS) across experiments, time and lines (Fig. 1f,g), demonstrating reproducibility and stability. iNEs and iAS formed dense and uniformly distributed networks (Fig. 1h), indicating mature morphologies and absent necrosis. Hypoxia was largely absent in 3BTMs, unless cultured under hypoxic conditions (Extended Data Fig. 1g). To increase culture complexity and physiology, and enable analysis of microglial states and functions, we next added iMGs to 3BTMs (Fig. 1i). iMGs migrated and spread in 3BTMs within days (Fig. 1j and Extended Data Fig. 1h–k), resembling tiling in the brain. iMG incorporation was improved in the presence of iAS (Extended Data Fig. 1l), and reproducible across lines, yielding consistent iNE:iMG ratios (Fig. 1k). Triple co-cultures could be maintained for more than 6 months without necrotic core, and with dense iNE and iAS networks (Extended Data Fig. 1m). Strikingly, iMGs adopted increasingly ramified morphologies (Fig. 1l,m) and expressed the homeostatic marker P2RY12 in the membrane of more than 75% of cells at 6 months (Fig. 1n), suggesting a broadly present homeostatic state in vitro, induced in organoids only by transplantation in vivo^12^. To further investigate iMG homeostasis and functionality, we analyzed membrane-bound enhanced yellow fluorescent protein (eYFP)-expressing iMGs in 3BTMs at 3 months using two-photon live-cell imaging. Strikingly, after focal laser injury, iMGs immediately extended processes toward the lesion, leading to complete ensheathment after 10–12 min (Fig. 1o and Supplementary Video 4). Quantifying movement speed, we found similar values for process extension and retraction under baseline conditions, and higher speeds after injury (Fig. 1p), as in vivo^12,24^. Thus, iMGs in 3BTMs can launch an in vivo-like injury response, confirming the presence of functional P2RY12 on the cell surface required for this rapid reaction^25^. iMGs were present in 3BTMs for over 6 months and slowly decreased over time (Extended Data Fig. 1n), possibly because of senescence or migration toward a growth factor gradient into the medium. However, survival was higher than in current in vitro models^12,13^, enabling analyses at advanced ages of 3 to 6 months. In addition, iMGs can be incorporated into 3BTMs later, for example, at 5 months, leading to a dense, ramified population at 6 months (Extended Data Fig. 1o,p). P2RY12 expression was similar to iMGs at 1 month (Extended Data Fig. 1q). Finally, we found consistently low levels of cell death in 3BTMs, especially when all three cell types were present (Fig. 1q and Extended Data Fig. 1r), suggesting that iMGs both benefit from and contribute to a physiological tissue environment, for example, by phagocytosis of potentially toxic degradation products, dead cells and debris. In summary, we established a highly reproducible and controllable 3D cortical tissue model containing iNEs, iAS and iMGs with mature cell morphologies.

### Maturation of 3BTMs increases similarity to human brain tissue

**Fig. 2:**
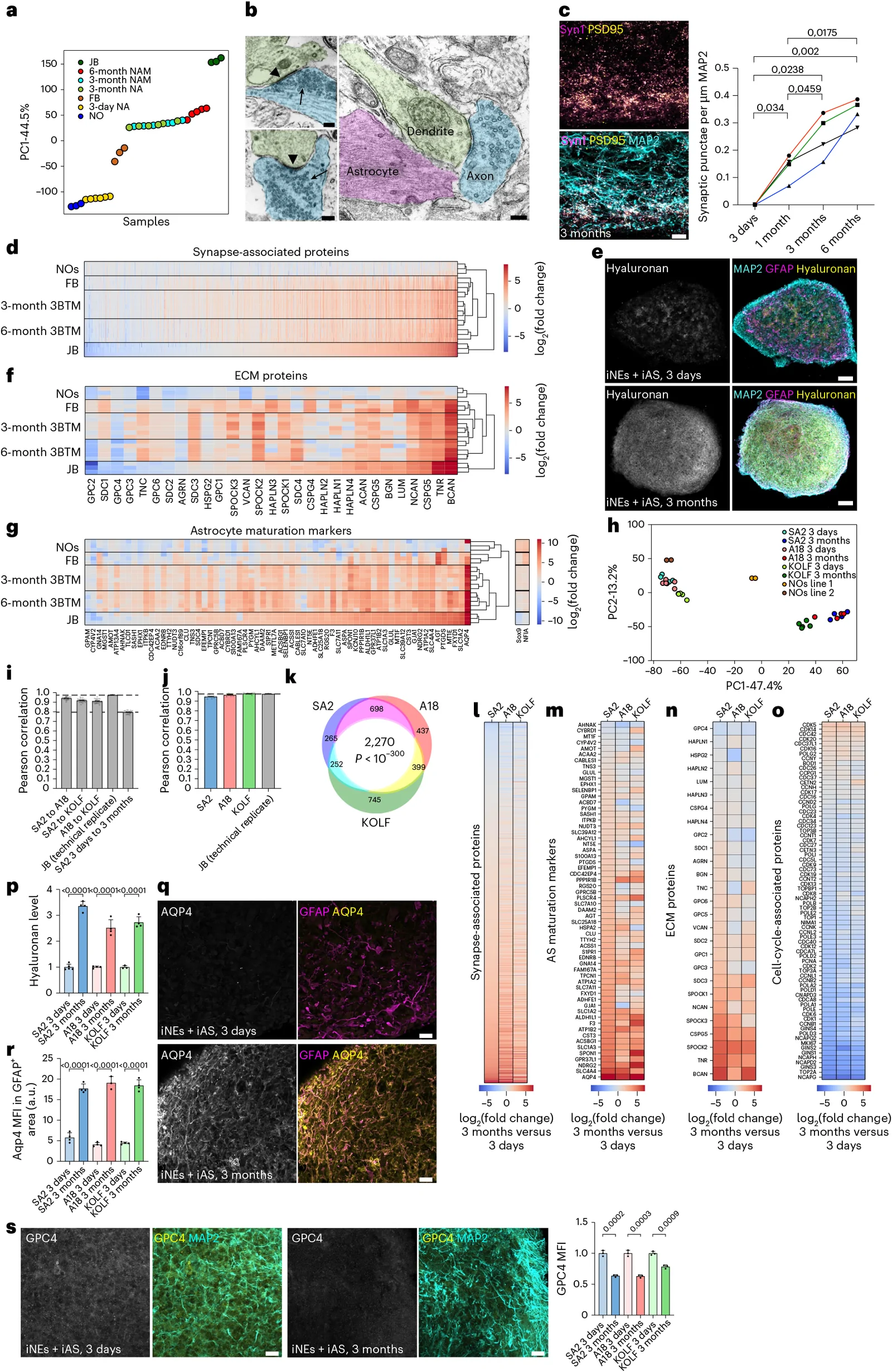
Maturation of 3BTMs increases similarity to human brain tissue. **a**, PCA of proteomic dataset 1 based on all proteins after imputation. NO (day 90, three technical replicates), 3 day NA = 3-day-old 3BTMs of iNEs + iAS (six biological replicates), FB (GW = 15–17, three biological replicates), 3 month NA = 3-month-old 3BTMs of iNEs + iAS (6 biological replicates), 3-month NAM = 3-month-old 3BTMs of iNEs + iAS + iMGs (six biological replicates), 6-month NAM = 6-month-old 3BTMs of iNEs + iAS + iMGs (five biological replicates), JB (6 years, three technical replicates). **b**, Electron micrographs of 3BTM at 3 months, showing formation of synapses (left) with presynaptic terminals (arrows) and postsynaptic densities (arrowheads) and a tripartite synapse (right) made of presynaptic axon, postsynaptic dendrite and adjacent iAS (in purple). **c**, IF staining of 3BTM at 3 months for presynaptic Syn1, postsynaptic PSD-95 and MAP2 (left). Right: Quantification of colocalized puncta, normalized to MAP2 over time (*n* = 4 experiments (biological replicates) represented by the colored lines with two 3BTMs each; repeated-measures one-way ANOVA with Tukey’s multiple comparisons test). **d**, Heatmap showing the log_2_(fold change) in protein levels of synapse-associated proteins found in proteomics datasets compared to 3-day-old iNEs + iAS cultures (set to zero). Right: Hierarchical clustering of the different samples. **e**, IF staining of iNE + iAS 3BTMs at 3 days and 3 months, showing deposition of hyaluronan. **f**, Heatmap and clustering as in **d**, showing the log_2_(fold change) of ECM proteins. **g**, Heatmap and clustering as in **d**, showing the log_2_(fold change) in astrocyte maturation markers and the housekeeping/identity proteins Sox9 and NFIA. **h**, PCA of proteomic dataset 2 based on all proteins after imputation. NO (day 90, 2 lines each run in technical duplicates), 3BTMs: *n* = 5 experiments (biological replicate) with three 3BTMs pooled per experiment and line. **i**, Pearson correlation coefficients of log_2_ label-free quantification (LFQ) intensities of all detected proteins between the different cell lines. Pearson correlation of technical replicates of JB samples (upper dashed line) and between 3-day and 3-month samples of the SA2 line (lower dashed line) shown for comparison (*n* = 25 comparisons (SA2 to A18/KOLF and A18 to KOLF); *n* = 20 comparisons (SA2 3 days to 3 months); *n* = 3 comparisons (JB)). **j**, Pearson correlation as in **i** between biological replicates of each line, the dashed line showing correlation between the technical replicates of JB samples for comparison (*n* = 10 (SA2, A18, KOLF), *n* = 3 (JB)). **k**, Venn diagram showing overlap of upregulated proteins in 3-month versus 3-day samples for all three lines; the number of proteins per set is indicated. A hypergeometric test was used to assess statistical significance of the triple overlap. **l**, Heatmap showing log_2_(fold change) in protein levels of synapse-associated proteins found in the proteomics dataset at 3-month-old compared with 3-day-old 3BTMs (set to zero) for each cell line. **m**, Heatmap as in **e** showing the log_2_(fold change) in astrocyte maturation markers. **n**, Heatmap as in **e** showing the log_2_(fold change) in ECM proteins. **o**, Heatmap as in **e** showing the log_2_(fold change) in cell-cycle-associated proteins. **p**, Quantification of IF staining for hyaluronan in all three cell lines at 3 days and 3 months (*n* = 3 (KOLF 3 days) *n* = 5 (SA2 3 days) and *n* = 4 (remaining) experiments; two 3BTMs were used per line and time point, unpaired *t*-tests). **q**, IF staining of iNE + iAS 3BTMs at 3 days and 3 months for Aqp4 and GFAP. **r**, Quantification of Aqp4 mean fluorescence intensity (MFI) in the GFAP^+^ area in all three cell lines (*n* = 3 (A18 3 months) and *n* = 4 (remaining) experiments; two 3BTMs per line and time point, unpaired *t*-test). **s**, IF staining of 3BTMs at 3 days and 3 months showing the ECM proteins GPC4 and MAP2. Right: Quantification of GPC4 MFI for each line (*n* = 3 experiments with two 3BTMs per line and time point, unpaired *t*-test). The bar graphs are presented as the mean + s.d. **b**, Scale bar, 200 nm. **c**, Scale bar, 10 µm. **e**, Scale bar, 100 µm. **q**,**s**, Scale bar, 20 µm. Source data

**Extended Data Fig. 2:**
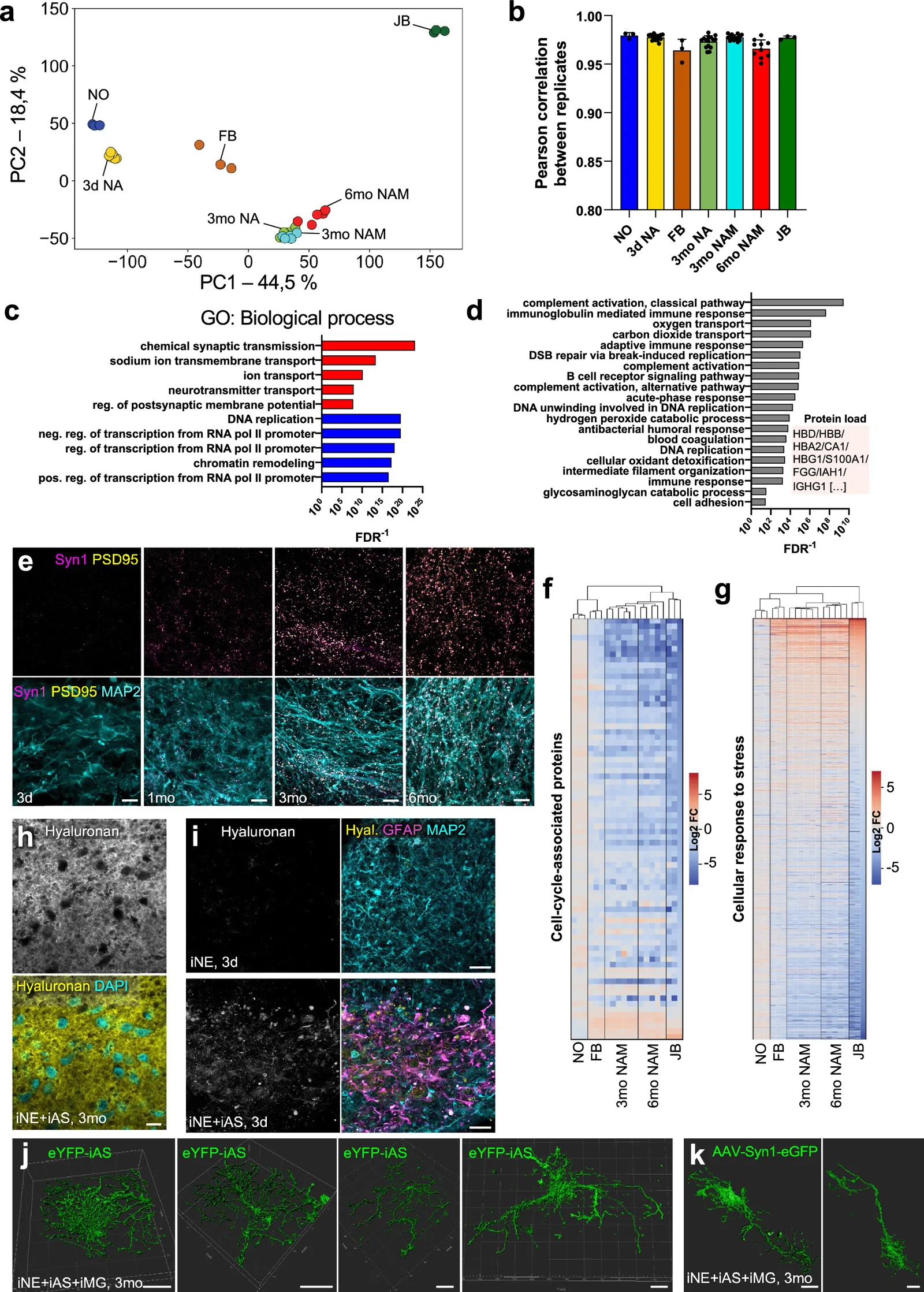
Mass spectrometry analysis of 3BTMs and morphologies of individual iNE and iAS. (**A**) Principal component analysis (PCA) of proteomic dataset based on all proteins after imputation showing principal components PC1 and PC2. (**B**) Pearson correlation of log2 LFQ intensities of all detected proteins between biol. replicates of each sample (tech. repl. for NO and JB). Data presented as mean + SD (n = 3 (NO, FB, JB), n = 15 (3 d NA, 3mo NA, 3mo NAM), n = 10 (6mo NAM). (**C**) Gene ontology (GO-BP) analysis of top proteins influencing PC1, shown are processes in which upregulated proteins (red) or downregulated proteins (blue) are enriched, sorted by false-discovery rate (FDR). (**D**) Gene ontology analysis (GO-BP) of top proteins influencing PC2, sorted by false discovery rate (FDR). A subset of the top proteins contributing to PC2 load is shown. (**E**) IF stainings of SA2 3BTM over time for presynaptic Synapsin-1 (Syn1), postsynaptic PSD-95, and MAP2, scale bars 10 µm. (**F**) Heatmap showing log2-fold changes (log2FC) in protein levels of cell-cycle-associated proteins found in proteomics dataset compared to 3-day-old iNE+iAS 3BTM (set to zero). Hierarchical clustering of the different samples on the right. (**G**) Heatmap as in (**D**) showing proteins in GO term “cellular response to stress”. (**H**) IF staining showing extracellular deposition of hyaluronan, as seen by exclusion of staining in DAPI-positive cell bodies, scale bar 10 µm. (**I**) IF staining for hyaluronan in 3BTMs containing only iNE or iNE+iAS at 3 days, scale bars 20 µm. (**J**) 3D rendering of eYFP-labelled, single iAS, scale bars 50 µm. (**K**) 3D rendering of AAV-hSyn1-eGFP-labelled, single iNE, scale bars 20 µm. Source data

We next hypothesized that the stable and postmitotic environment 3BTMs provide may allow all incorporated cell types to develop similar phenotypes to their in vivo counterparts, resulting in high similarity to brain tissue. Therefore, we performed mass spectrometry (MS) analysis of 3BTMs at 3 days, 3 months and 6 months, and compared them to NOs (day 90), human fetal brain (FB) (gestational weeks (GWs) 15–17) and juvenile human brain (JB) (6 years). Principal component analysis (PCA) (Fig. 2a and Extended Data Fig. 2a) revealed dense clustering of 3BTMs of similar ages and replicates, showing high reproducibility, further confirmed by strong correlation between 3BTM biological replicates, reaching coefficients similar to technical replicates of NOs and JBs (Extended Data Fig. 2b). Characterizing principal component 1, we found that samples separated in a putative maturation-dependent manner, with NO and 3-day samples on one end, and 3-month and 6-month 3BTM samples placed between FB and JB samples (Fig. 2a). Gene ontology (GO) enrichment analysis for biological processes (GO-BP) confirmed that upregulated proteins were involved in maturation-dependent processes, including synaptic transmission and ion/neurotransmitter transport, whereas downregulated proteins were involved in cell-proliferation-related processes (Extended Data Fig. 2c). We focused on principal component 1 because principal component 2 explained much less variance (Extended Data Fig. 2a) and was driven by ‘blood-dependent’ processes or DNA replication irrelevant for matured 3BTMs (Extended Data Fig. 2d).

To confirm the maturation of 3BTMs over time, we analyzed synapse formation as the main process driving principal component 1. Electron microscopy analysis showed differentiated synapses (Fig. 2b, left) and occasionally brain-typical tripartite synapses (Fig. 2b, right), suggesting iNE network formation and neuron–glia interactions. Additionally, immunostaining for presynaptic Synapsin-1 (Syn1) and postsynaptic PSD95 revealed increasing colocalization over time (Fig. 2c and Extended Data Fig. 2e), indicating synapse formation. For a global overview of synaptic components, we analyzed synapse-associated proteins in our proteomic dataset and found many strongly upregulated in 3-month-old and 6-month-old cultures (Fig. 2d and Supplementary Table 1), positioning them closer to FB and JB samples than to organoids or the 3-day cultures used for comparison. We next confirmed downregulation of cell-cycle-associated proteins in older cultures, again indicating higher maturity and similarity to JB and confirming postmitotic state (Extended Data Fig. 2f and Supplementary Table 1). To check if stress markers are increased in 3BTMs, we analyzed proteins found in the GO term ‘cellular response to stress’. We found similar levels in 3BTMs, FB and JB (Extended Data Fig. 2g, and Supplementary Table 1), again indicating a physiological environment in 3BTMs. Interestingly, ‘cell adhesion’ was another altered process in our GO-BP analysis, and ‘extracellular region’ and ‘extracellular space’ were enriched as cellular compartments (Supplementary Table 1). Therefore, we investigated tissue maturation based on brain ECM formation in 3BTMs. Brain ECM is essential for several cell functions^5^ but could not yet be replicated in vitro^26^. Therefore, we avoided exogenous ECM during culture generation and hypothesized that the cells would produce their own, brain-like matrix. Strikingly, we found that hyaluronan, a main component of brain ECM, was present at low levels on day 3 and strongly increased at 3 months to a homogeneous staining (Fig. 2e) sparing cell bodies (Extended Data Fig. 2h), indicating deposition in an ECM-like structure. Hyaluronan levels were strongly increased in the presence of iAS (Extended Data Fig. 2i), suggesting iAS as main producers. For a global overview of ECM formation, we analyzed ECM-related proteins using proteomics. We detected a variety of ECM molecules, including brain-specific or brain-enriched core proteins of chondroitin sulfate proteoglycans, such as neurocan (NCAN), brevican (BCAN) and CSPG5, the linker proteins tenascin (TNC) and HAPLNs, and other relevant molecules, such as hyaluronan sulfate proteoglycans (Fig. 2f and Supplementary Table 1). We found strong upregulation of many brain ECM proteins in our 3-month and 6-month samples, again increasing the similarity to the FB and JB samples (Fig. 2f). Moreover, a set of downregulated proteins in aged cultures matched reduced expression in JB versus FB, for example, GPC-2 and GPC-4, especially at 6 months, suggesting both buildup and brain-like remodeling of the cultures’ ECM.

As astrocytes are the main drivers of brain maturation, we analyzed mature human astrocyte markers described previously^27^. Proteomics showed strong upregulation of most astrocyte-specific maturation markers at 3 and 6 months, whereas housekeeping proteins stayed largely constant (Fig. 2g and Supplementary Table 1). This resulted in higher similarity to JB than to FB samples according to hierarchical clustering, suggesting high maturity of iAS in 3BTMs. To further study iAS maturity, we incorporated few membrane-eYFP-expressing iAS to investigate single-cell morphologies. We found different types of iAS, including highly ramified, ‘bushy’ cells resembling protoplasmic astrocytes in the human brain^28^, less ramified cells extending several processes over 200–300 µm and cells with few processes over 600–700-µm distance (Extended Data Fig. 2j). Similarly, single iNE morphology revealed using AVV-Syn1-eGFP transduction indicated mature cell morphologies with branched dendrites and a long, ramified axon (Extended Data Fig. 2k), resembling pyramidal neurons. Together, our analysis confirms mature iNE and iAS morphologies, protein composition and ECM formation, similar to JB cells in vivo.

To confirm reproducibility of proteome composition and maturation, we repeated analyses with 3BTMs from all three iPSC lines at 3 days and 3 months and compared them to NOs from two different iPSC lines. PCA analysis showed close clustering of all 3BTMs, especially compared to the NO samples (Fig. 2h). This was confirmed by high overall correlation coefficients of 3BTMs between lines (Fig. 2i), with far lower inter-line than over-time variance and coefficients similar to technical replicates of JB run simultaneously. Similarly, intra-line variance was low and similar to technical replicates (Fig. 2j). To confirm maturation across lines, we next compared all proteins upregulated from 3 days to 3 months and found a strong overlap between lines (Fig. 2k and Supplementary Table 1), also validating high reproducibility. This was substantiated by highly concordant changes of maturation markers, including synapse-associated, astrocyte-maturation-associated, ECM-associated and cell-cycle-associated proteins (Fig. 2l–o and Supplementary Table 1) across lines. Immunostaining confirmed reproducible upregulation of the ECM component hyaluronan (Fig. 2p) and astrocyte-maturation-related AQP4 (Fig. 2q,r), and downregulation of the ECM protein GPC-4 (Fig. 2s).

Together, our analyses suggest that 3BTM proteomes are highly reproducible, creating a brain-tissue-like environment inducing maturation of the cells, gradually increasing similarity to their in vivo counterparts.

### iMGs mature in 3BTMs and adopt transcriptomic signatures similar to the human brain

**Fig. 3:**
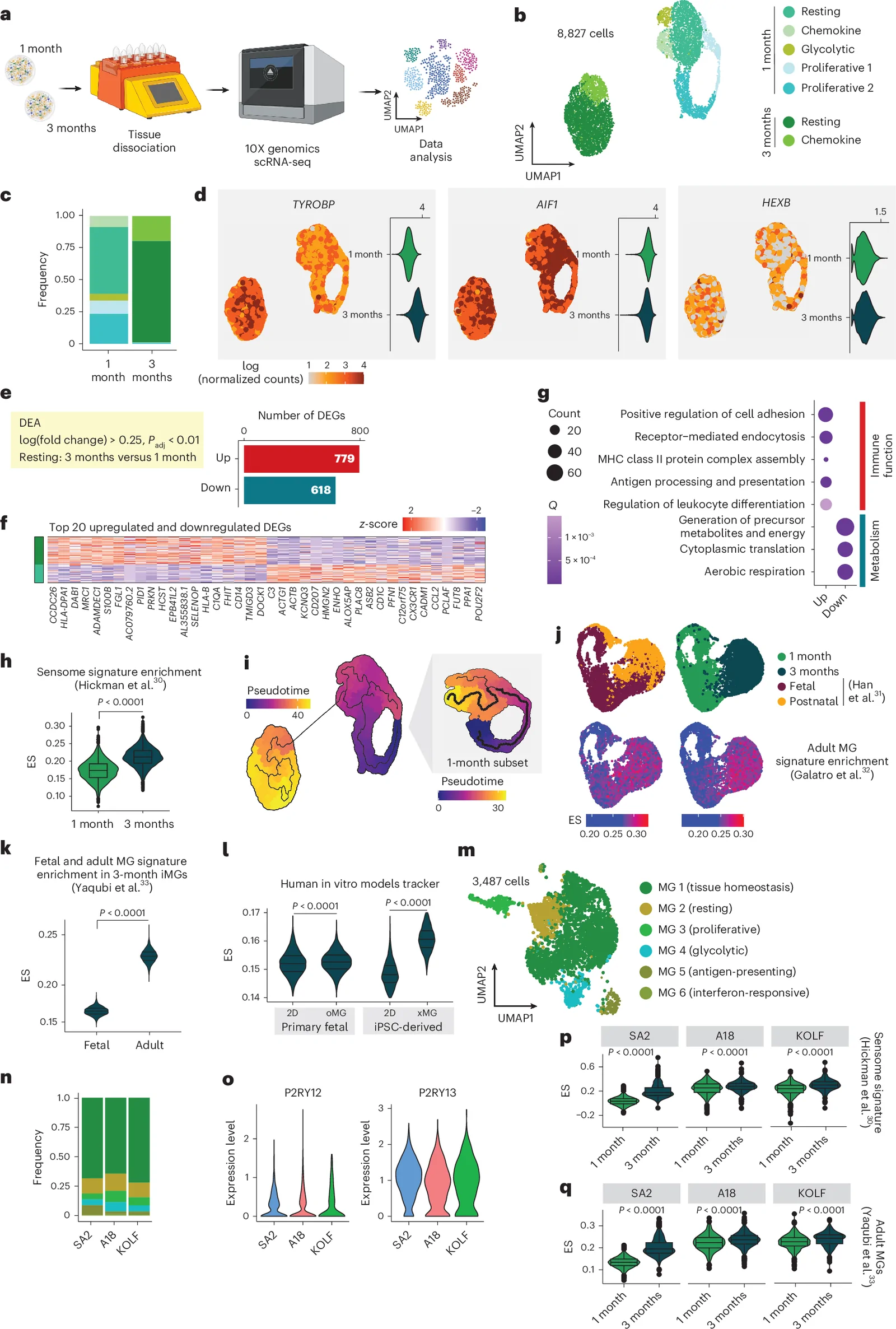
iMGs mature in 3BTMs and adopt transcriptomic signatures similar to the human brain. **a**, Schematic overview of the scRNA-seq experiment to investigate iMG phenotypes in 3BTMs at 1 and 3 months. iMGs from two independent experiments (biological replicates) were used. Created in BioRender (https://BioRender.com/i2438ox). **b**, UMAP representation of 8,827 cells and cluster annotation of iMGs at 1 and 3 months. **c**, Cell state frequencies reported for iMGs at 1 and 3 months separately. **d**, Normalized expression of key MG markers (*TYROBP*, *AIF1*, *HEXB*) both UMAP-projected and as a violin plot in iMGs at 1 and 3 months. **e**, Number of DEGs obtained by comparing 1-month and 3-month resting clusters, including reference statistical thresholds (MAST test with Bonferroni correction for multiple comparisons). **f**, Heatmap showing the normalized scaled expression of the top 20 upregulated and downregulated DEGs. **g**, ORA (GO-BP) of DEGs showing enrichment of upregulated genes in immune-function-related processes and of downregulated genes in metabolism-related processes. **h**, Violin plot showing the MG sensome signature^30^ enrichment scores (ES) for iMGs at 1 and 3 months (Wilcoxon rank-sum test). **i**, Pseudotime analysis highlighting phenotypic progression of iMGs from 1 month to 3 months. Right: One-month subset. **j**, Top: UMAP plot after integration of a human fetal and postnatal, ex vivo MG dataset (Han et al.^31^) with iMGs at 1 and 3 months, split according to dataset. Bottom: Same UMAP plot showing the projected adult MG signature (Galatro et al.^32^) ES, split according to dataset. **k**, Violin plot reporting fetal versus adult MG signature (extracted from Yaqubi et al.^33^) ES in iMG at 3 months, (Wilcoxon rank-sum test). **l**, In vitro tracker reporting the ES of iMGs at 3 months across different model-specific signatures derived from the integrated dataset by Popova et al.^13^, including primary human fetal MGs cultured in 2D (primary fetal) or incorporated into cortical organoids (oMGs, primary fetal), and human iMG cultured in 2D (iPSC-derived) or xenotransplanted into the mouse brain (xMGs, iPSC-derived) (Wilcoxon rank-sum test). **m**, UMAP representation of 3,487 cells and cluster annotation of iMGs from 3BTMs of the WT lines SA2, A18 and KOLF at 3 months. **n**, Cluster frequencies reported for all three lines separately. **o**, Violin plots showing expression of the homeostatic markers P2RY12 and P2RY13 in all three lines. **p**, Violin plots showing the MG sensome signature^30^ ES for iMGs at 1 and 3 months for each line (Wilcoxon rank-sum tests. **q**, Violin plots showing the adult MG signature (extracted from Yaqubi et al.^33^) ES for iMGs at 1 and 3 months for each line (Wilcoxon rank-sum tests). Data in the box plots are presented as the median, 25th and 75th percentiles (box) ± 1.5 times the interquartile range (IQR) (whiskers). Panel **a** created in BioRender; https://biorender.com/i2438ox (2026). Source data

**Extended Data Fig. 3:**
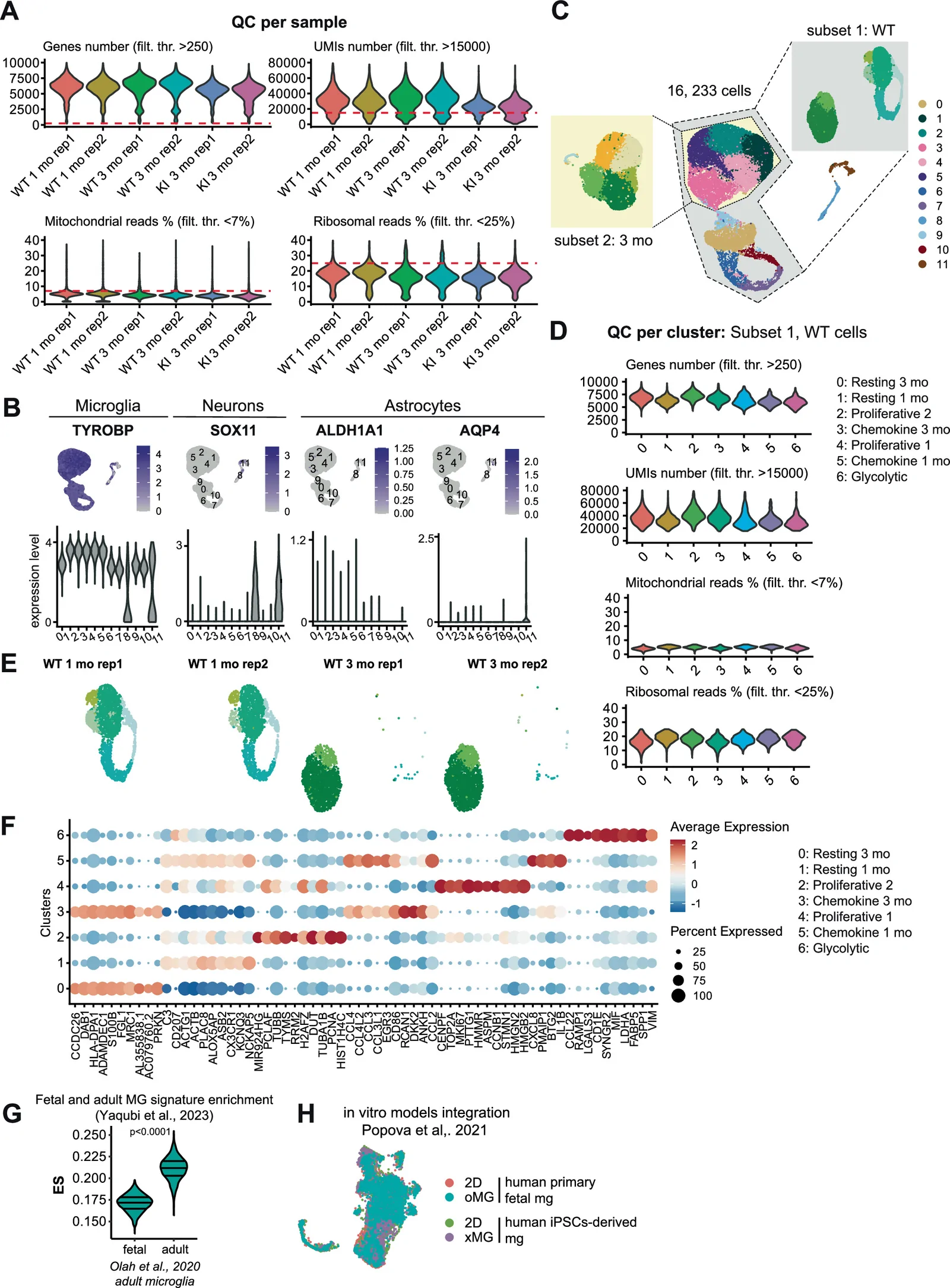
Single-cell RNA sequencing analysis of iMG from 3BTMs at 1 and 3 months. (**A**) Per cell quality controls (QC) across all clusters including counts per cell (>250), UMIs per cell (>15,000), mitochondrial reads (<7% per cell), and ribosomal reads (<25% per cell). (**B**) Expression of markers for microglia (*TYROBP*), neurons (*SOX11*), and astrocytes (*ALDH1A1*, *AQP4*) across the identified dataset clusters. Only pure iMG clusters were selected for downstream analyses (clusters 8 and 11 were excluded). (**C**) UMAP-based overview of the overall dataset subsetting strategy: clusters 8 and 11 were excluded from the initially QC-filtered dataset focusing on iMG analysis, considering their expression of neuronal and astrocytes markers. WT (Fig. 3) and 3mo (Fig. 5) subsets were produced for downstream analyses. (**D**) Per cell QC as in (**A**) across the WT subset clusters (Fig. 3b). (**E**) UMAP reported in Fig. 3b split by biological replicates. (**F**) Top 10 marker genes (only positive, detected in >25% of cells with log_2_FC > 0.25, ordered by log_2_FC) across the WT subset clusters. (**G**) Violin plot showing enrichment of adult microglia (dataset from Olah et al.^34^) for the fetal and adult MG signatures from Yaqubi et al., used as external validation for signatures from the dataset of Yaqubi et al. Box plots show median, 25^th^ and 75^th^ percentiles (Wilcoxon rank-sum test). (**H**) UMAP representation of the integrated Popova et al.^13^ *in vitro* model dataset, including primary human fetal MG cultured in 2D (2D, human primary fetal) or incorporated into cortical organoids (oMG, human primary fetal) as well as human iMG either cultured in 2D (2D, human iPSCs-derived) or xenotransplanted into mouse brain (xMG, human iPSCs-derived). Source data

To characterize iMG maturation and homeostasis already suggested by ramified morphology and P2RY12 expression, (Fig. 1j), we assessed iMG transcriptomes at 1 and 3 months (Fig. 3a) using single-cell RNA sequencing (scRNA-seq). Focusing on iMGs, we performed gentle dissociation, including actinomycin D, to prevent artificial activation^29^. Consequently, after quality filtering (Extended Data Fig. 3a), iMG-optimized extraction resulted in preferential recovery of iMGs over iNEs and iAS (Extended Data Fig. 3b), which because of their complex structure and connectivity probably require harsher dissociation for efficient retrieval. We excluded iNE, iAS and mixed clusters from the analysis and focused on the wildtype (WT) iMG subset at 1 and 3 months (Extended Data Fig. 3c). After quality filtering (Extended Data Fig. 3d), we identified 8,827 iMGs mainly separating according to 3BTM age (Fig. 3b). Cluster composition and uniform manifold approximation and projection (UMAP) from two independent experiments were highly similar, demonstrating reproducibility of iMG states in 3BTMs (Extended Data Fig. 3e). Furthermore, upon clustering, similar major cell states were identified at 1 and 3 months. To annotate those, we used marker genes (Extended Data Fig. 3f) and identified resting and chemokine-associated clusters (Extended Data Fig. 3f) at both 1 and 3 months and additional proliferative and a small glycolytic cluster at 1 month (Fig. 3b,c). All clusters expressed typical MG markers *AIF1*, *TYROBP* and *HEXB* (Fig. 3d). Differential gene expression analysis (DEA) on 3-month versus 1-month resting iMG yielded 779 upregulated and 618 downregulated genes, suggesting a phenotypic transition with age (Fig. 3e,f). Overrepresentation analysis (ORA) of differentially expressed genes (DEGs) indicated a metabolic shift over time and upregulation of genes involved in immune-related functions, (Fig. 3g), suggesting acquisition of an immune-sensing state, a hallmark of MG maturation, enabling tissue surveillance and reaction to aversive cues^24^. When comparing our iMG signatures at 1 and 3 months to a published sensome signature^30^, we found significantly higher enrichment at 3 months, indicating increased maturity and ability to interact with their environment (Fig. 3h). Pseudotime analysis validated the phenotypic progression of iMGs (Fig. 3i) from proliferative cells at 1 month over the intermediate 1-month resting state toward the 3-month states. Supporting the cluster annotations in Fig. 3b for 1-month iMGs, pseudotime analysis of the 1-month subset highlighted an unfolding of phenotypes from a proliferative state through the resting state, branching into activated and glycolytic clusters. (Fig. 3i). To validate iMG maturation and compare the cells to MGs from the human brain, we integrated our dataset with postmortem human fetal (GW 10–14) and postnatal (14–23 years) MG datasets^31^ and assessed individual cell enrichment for the Galatro et al.^32^ adult MG signature. Intriguingly, iMGs from 3-month-old cultures clustered along postnatal MGs; iMGs from 1 to 3 months showed a similar transition toward a more mature phenotype as observed from fetal to postnatal MGs, attested by increased enrichment for the Galatro signature with age in both datasets (Fig. 3j). To confirm the progressive acquisition of a postnatal rather than fetal profile at 3 months in culture, we compared our iMGs to fetal and adult human MG signatures from an independent study^33^, after confirming suitability of the approach with another postmortem dataset^34^ (Extended Data Fig. 3g). We found strongly increased enrichment of our 3-month iMGs for the adult compared to fetal signature (Fig. 3k), demonstrating the maturity of iMG in 3BTMs. Finally, we compared our 3-month iMG signature to different human in vitro model signatures derived from a published dataset^13^ (Extended Data Fig. 3h), including primary fetal MGs cultured in 2D or incorporated into NOs, and iMGs cultured in 2D or xenotransplanted into the mouse brain. We found the strongest enrichment for xenotransplanted iMGs (Fig. 3l), which more closely resemble in vivo MGs^35^.

Lastly, we repeated the transcriptomic analysis in the A18 and KOLF lines together with SA2 using another scRNA-seq technology (BD Rhapsody) and found a highly similar subcluster composition and identity across lines, with each line contributing to all clusters (Fig. 3m,n). All lines showed similar expression levels of the homeostatic markers P2RY12 and P2RY13 (Fig. 3o). In addition, we confirmed increased enrichment at 3 months compared to 1 month for the sensome (Fig. 3p) and adult human MG signatures^33^ (Fig. 3q), validating iMG maturation in 3BTMs across lines.

### iNEs, iAS and iMGs in 3BTMs acquire functionalities typical for mature brain cells

**Extended Data Fig. 4:**
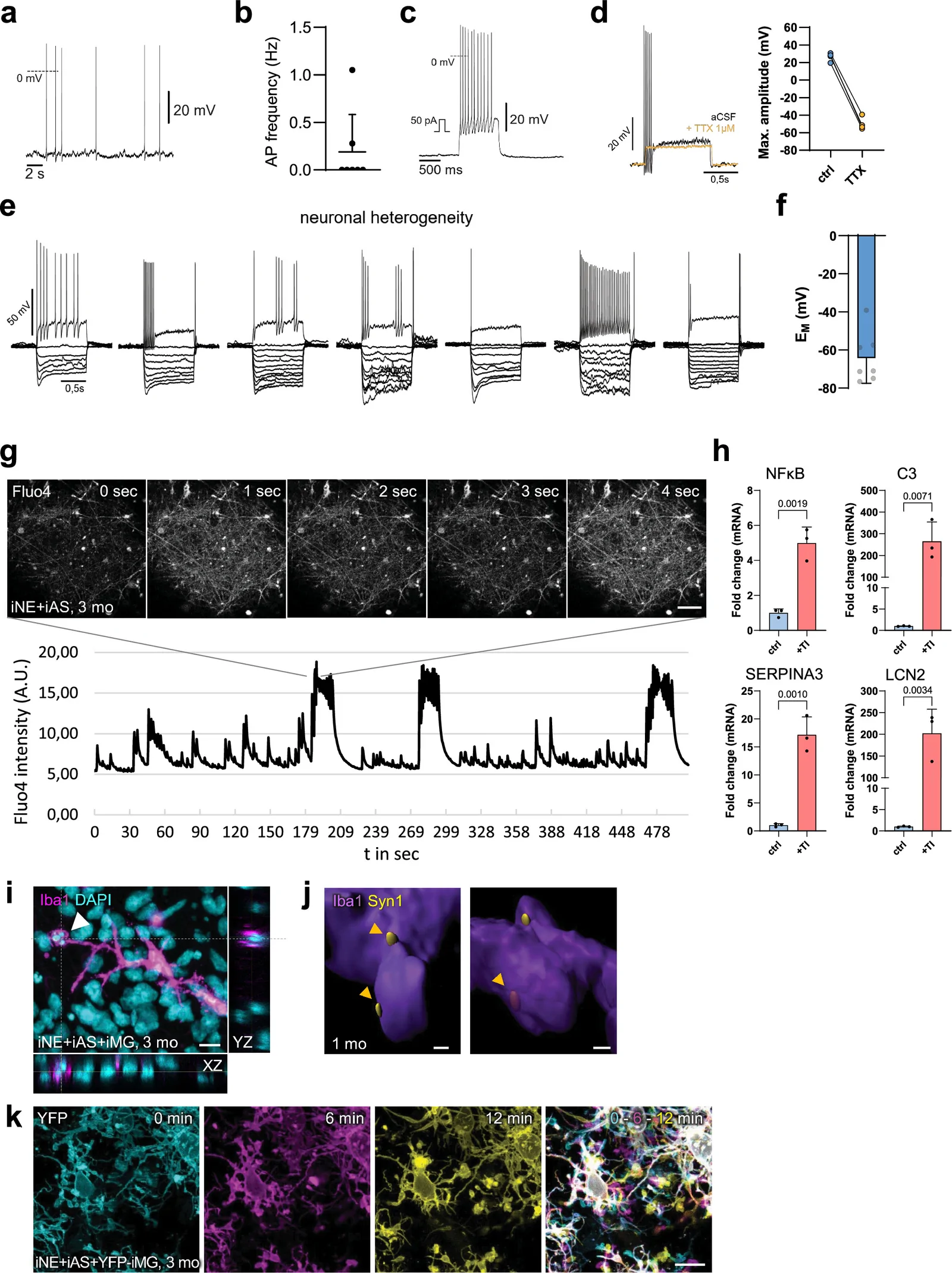
Functional assays for iNE and iAS. (**A**) Representative patch-clamp measurement of single iNE in iNE+iAS+iMG 3BTMs at 3 months showing spontaneous action potentials. (**B**) Spontaneous action potential (AP) frequency observed in patch-clamp measurements of single iNE in iNE+iAS+iMG 3BTMs at 3 months (n = 7 cells measured from two 3BTMs). (**C**) Representative patch clamp measurements as in (**A**) with current injection showing evoked action potentials. (**D**) Application of tetrodotoxin (TTX) inhibits action potential firing, quantification on the right (n = 4 cells from 2 experiments (biol. repl.). (**E**) Evoked APs observed as in (**C**) showing heterogeneous firing patterns in response to depolarizing stimuli. (**F**) Resting membrane potential (E_M_) measurements of single iNE as in (**A**) (n = 7 cells measured from 2 experiments (biol. repl.)). (**G**) Top: Example images of live cell calcium imaging using Fluo-4 in iNE+iAS+iMG 3BTMs at 3 months showing spontaneous and synchronized activity. Bottom: Quantification of Fluo-4 intensity over time, timeframe shown above is marked by lines. (**H**) qPCR analysis for astrocytic activation markers in iNE+iAS 3BTM at 1 month of age treated with PBS (ctrl) or TNFα + IL1α (TI), normalized to *SOX9* as astrocytic reference gene (n = 3 experiments (biol. repl.) with 7-10 3BTMs pooled for RNA extraction, unpaired *t*-tests). (**I**) IF staining for DAPI-positive DNA and Iba1-positive iMG in 3mo 3BTM showing microglial process engulfing a dead cell’s nucleus (as seen by bright and fragmented DAPI staining, arrowhead), orthogonal projections in YZ and XZ, scale bar 10 µm. (**J**) 3D surface rendering of IF staining for Synapsin-1 (Syn1)-positive synaptic material and Iba1-positive iMG at 1 month showing close association (left) and engulfment (right) of synaptic material by iMG, scale bars 0,5 µm. (**K**) 2-Photon live-cell imaging of iNE+iAS+iMG 3BTMs at 3 months with iMG expressing membrane-eYFP. Pictures showing different, color-coded timepoints (0, 6, 12 min), merged image on the right showing movement by color-coding whereas stable structures appear white, scale bar 20 µm. Data in bar graphs presented as mean + SD. Source data

To investigate if the observed maturation in gene expression also causes functional maturation, we performed electrophysiological patch-clamp measurements with iNEs. We observed spontaneous action potentials (Extended Data Fig. 4a,b); we evoked action potentials using current injection (Extended Data Fig. 4c) and blocked them using tetrodotoxin (Extended Data Fig. 4d), demonstrating dependence on voltage-gated sodium channels. We also observed heterogeneous firing patterns in response to depolarizing stimuli in different iNEs (Extended Data Fig. 4e), possibly indicating the presence of various neuronal subtypes described in the human cortex^36^. Furthermore, cells showed a resting membrane potential of ~−65 mV (Extended Data Fig. 4f), similar to mature neurons in the human cortex^36^. Lastly, we performed whole-mount calcium imaging and detected spontaneous and synchronized activity (Extended Data Fig. 4g and Supplementary Video 1). Altogether, the data suggest that 3BTMs contain mature iNEs, which express typical ion channels underlying intrinsic active and passive neuronal membrane properties, and contain synapses enabling functional network formation.

To investigate iAS functionality, we focused on the cells’ ability to react to inflammatory cytokines. We treated 3BTMs made of iNEs and iAS with tumor necrosis factor and Interleukin-1 alpha to promote a reactive astrocyte state. After 24 h, we found robust increases in typical inflammation and reactive astrocyte markers *NFκB*, *C3*, *SERPIN3A* and *LCN2* (Extended Data Fig. 4h), confirming the cells’ ability to switch to an activated state.

Finally, we confirmed the functionality of iMGs besides the reaction to adverse stimuli (Fig. 1). As innate immune cells, iMGs constantly surveil the environment with their processes and remove debris and excess synapses^37^. To investigate these functions, we first performed co-immunostaining for iMGs and cell nuclei or presynaptic Syn1 and observed iMGs engulfing the nucleus of a dead cell (Extended Data Fig. 4i), and iMGs engulfing or closely contacting synaptic material (Extended Data Fig. 4j and Supplementary Video 2). When looking at baseline conditions in 3BTMs, membrane eYFP-labeled iMGs also constantly surveilled their environment using their ramified processes (Extended Data Fig. 4k and Supplementary Video 3), as in vivo^12,24^. Altogether, all cell types showed brain-like functionalities with remarkable reproducibility.

### Brain cells in 3BTMs show increased maturity, homeostasis and functionality compared to 2D co-cultures

**Extended Data Fig. 5:**
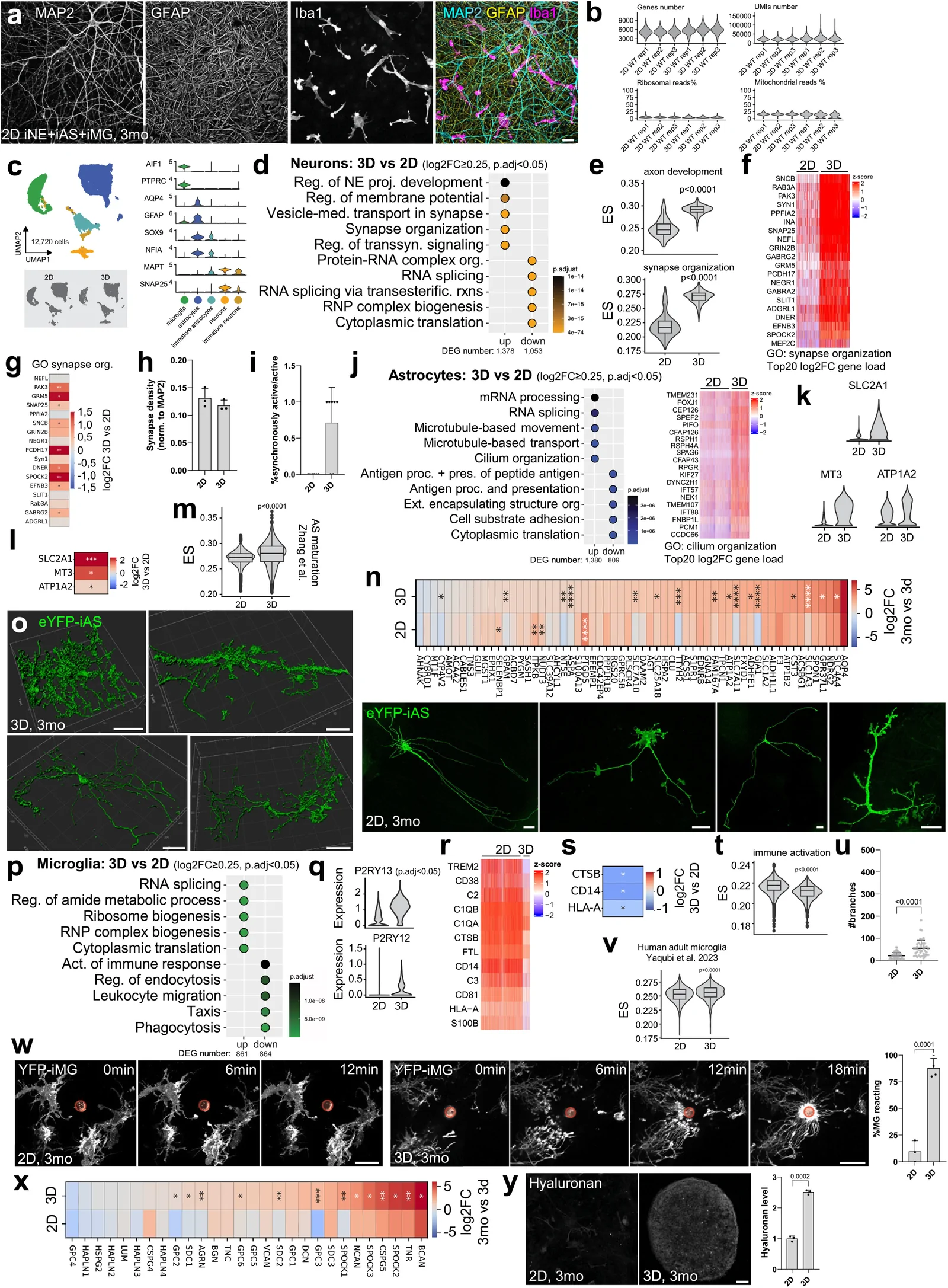
Brain cells in 3BTMs show increased maturity, homeostasis, and functionality compared to 2D co-cultures. (**A**) IF staining of 2D co-culture of iNE, iAS, and iMG at 3mo, scale bar 20 µm. (**B**) Per cell QC across samples of scRNAseq analysis of 2D co-cultures as in (**A**) and 3BTMs (3D) generated from the same cells, cutoffs: genes >500, UMIs >15,000, ribosomal reads <25%, mitochondrial reads <30%. (**C**) UMAP representation and cluster annotation (top left), and UMAP split by culture format (2D vs 3D, bottom left). Right: violin plots showing expression of cell type markers. (**D**) Differential gene expression analysis (DEA) of iNE from 3BTMs or 2D co-cultures (MAST test with Bonferroni correction for multiple comparisons). (**E**) Violin plots comparing signatures from iNE in 2D vs. 3BTMs with signatures containing axon development (top) or synapse organization genes (bottom) by enrichment score (ES) (Wilcoxon rank-sum test). (**F**) Heatmap showing expression of top DE genes in “Synapse organization”. (**G**) Heatmap showing log2FC in expression of proteins in term “synapse organization” (as in (**F**)). Only proteins detected in all replicates of both conditions are shown (n = 5 experiments (biol. repl.) with three 3BTMs or one 6-well well pooled for protein extraction per sample, unpaired *t*-tests). (**H**) Quantification of synapse density (colocalized Synapsin-1/PSD95-positive puncta, normalized to MAP2-positive area), (n = 3 experiments (biol. repl.) with two 3BTMs or coverslips each, paired *t*-test). (**I**) Quantification of synchronized activity in electrically active cultures based on Fluo-4 calcium imaging (n = 6 (2D) and n = 7 (3D) experiments (biol. repl.) with 3 3BTMs or 2 coverslips each). (**J**) Left: DEA as in (**D**) of iAS. Right: Heatmap showing expression of top DE genes in “Cilium organization” (MAST test with Bonferroni correction for multiple comparisons). (**K**) Violin plots showing expression of iAS-function-related DE genes (padj < 0.05). (**L**) Heatmap of proteins involved in iAS function. (n = 5 experiments, as in (**G**), unpaired *t*-tests). (**M**) Violin plots showing signature enrichment of iAS from 2D co-cultures vs. 3BTMs for a mature human AS signature^27^ (Wilcoxon test). (**N**) Heatmap showing log2FC in protein levels of AS maturation markers at 3mo vs. 3 d (n = 5 as in (**G**), unpaired *t*-tests). (**O**) 3D rendering of eYFP-labelled, single iAS in 3BTMs (4 left images) and IF staining of eYFP-labelled, single iAS in 2D co-cultures (4 right images), scale bars 50 µm. (**P**) DEA of iMG from 3BTMs or 2D co-cultures (MAST test with Bonferroni correction for multiple comparisons). (**Q**) Violin plots showing expression of homeostasis genes P2RY13 (top) and P2RY12 (bottom) (MAST test with Bonferroni correction for multiple comparisons). (**R**) Heatmap showing expression of DE genes related to MG activation. (**S**) Heatmap showing log2FC in expression of MG-activation-related proteins. Only significantly altered proteins detected in all replicates of both conditions are shown (n = 5 experiments, as in (**G**), unpaired *t*-tests). (**T**) Violin plots showing enrichments for immune activation signature in iMG (Wilcoxon rank-sum test) (**U**) Quantification of iMG branch number (n = 43 (2D) – 51 (3D) cells (biol. repl.), Mann-Whitney test). (**V**) Violin plots showing signature enrichments for a human adult microglia signature^33^ in iMG (Wilcoxon rank-sum test). (**W**) 2-Photon live-cell imaging of mem-eYFP-iMG in 2D co-cultures (left) or 3BTMs (right). Pictures showing reaction to focal laser injury (red circles), scale bars 50 µm. Far right: Quantification of reacting cells. (n = 3 (2D) and n = 4 (3D) experiments (biol. repl.), 1 3BTM or coverslip each, unpaired *t*-test). (**X**) Heatmap showing expression of brain ECM-related proteins at 3mo compared to 3 d (n = 5 as in (**G**), unpaired *t*-tests). (**Y**) IF staining of hyaluronan at 3mo, scale bar 100 µm. Quantification on the right (n = 3 experiments (biol. repl.) with two 3BTMs or one coverslip each, unpaired *t*-test). p-values for graphs G, M, O, T, Y: * p < 0.05, ** p < 0.01, *** p < 0.001, **** p < 0.0001. Bar graphs are presented as mean + SD. Box plots show median, 25^th^ and 75^th^ percentiles (box) ± 1.5x IQR (whiskers). Source data

To benchmark the properties of 3BTMs in comparison to 2D co-cultures of the same cells (Extended Data Fig. 5a), we performed transcriptomic, proteomic and functional analyses at 3 months. Adaptations to our dissociation protocol to increase the iNE and iAS yield allowed analyzing all cell types using scRNA-seq. We retrieved 12,720 high-quality cells of all three cell types as confirmed by typical marker expression (Extended Data Fig. 5b,c). Differential expression analysis of iNEs from 3BTMs versus 2D co-cultures showed increased expression of function-related pathways, including synapse organization and regulation of synaptic signaling in 3BTMs (Extended Data Fig. 5d). Enrichment analysis confirmed higher expression of gene signatures for axon development and synapse organization (Extended Data Fig. 5e,f), with similar effects found at the protein level (Extended Data Fig. 5g), suggesting overall improved functionality of iNEs in 3BTMs. To confirm this, we compared synapse density and synchronized network activity. Even though synapse density relative to MAP2 was similar (Extended Data Fig. 5h), network activity was different: 3BTMs regularly showed spontaneous, synchronized activity in calcium imaging, whereas 2D co-cultures only showed spontaneous firing of single cells at the same timepoint, indicating improved formation of functional synapses and neuronal interconnectivity in 3BTMs (Extended Data Fig. 5i and Supplementary Video 5). Altogether this suggests a more complex or mature iNE network in 3BTMs compared to 2D co-cultures.

In iAS, differential expression analysis of scRNA-seq data showed upregulation of genes involved in motility and cilium organization in 3BTMs (Extended Data Fig. 5j), both crucial for astrocytic support of neuronal function and astrocyte specification^38^, as well as downregulation of antigen processing and presentation, potentially indicating a less reactive state. We further found upregulation of markers for central astrocyte functions in 3BTMs, including *SLC2A1*, *MT3* and *ATP1A2* on RNA (Extended Data Fig. 5k) and protein level (Extended Data Fig. 5l). iAS in 3BTMs also showed higher enrichment for a mature human astrocyte signature^27^ (Extended Data Fig. 5m) and upregulation of several key astrocyte markers on protein level (Extended Data Fig. 5n), suggesting higher maturity of iAS in 3BTMs. This was further confirmed morphologically by higher complexity and ramification of iAS in 3BTMs (Extended Data Fig. 5o).

iMG from 3BTMs showed downregulation of genes involved in immune reactivity, including ‘activation of immune response’, ‘taxis’ and ‘phagocytosis’ compared with 2D (Extended Data Fig. 5p), indicating a more homeostatic state. This was confirmed by higher RNA expression of the homeostasis markers P2RY12 and P2RY13 (Extended Data Fig. 5q), downregulation of MG activation markers (Extended Data Fig. 5r), also on the protein level (Extended Data Fig. 5s), lower enrichment for an immune activation signature (Extended Data Fig. 5t) and morphological analysis showing higher number of branches in 3BTMs (Extended Data Fig. 5u). iMGs from 3BTMs further showed higher enrichment for an adult human MG signature^33^ (Extended Data Fig. 5v), suggesting a higher level of maturity. Functionally, iMGs in 2D co-cultures were largely unable to respond to a focal laser injury unlike cells in 3BTMs (Extended Data Fig. 5w), potentially because of the lack of P2RY12 expression (Extended Data Fig. 5r).

Finally, we compared brain ECM formation using proteomic analysis and found significantly higher levels of several key components, including BCAN, NCAN, TNC-R and CSPG5 in 3BTMs (Extended Data Fig. 5x), and higher levels of hyaluronan as crucial scaffolding component (Extended Data Fig. 5y).

Altogether this suggests a higher degree of maturity and functionality of all cell types in 3BTMs compared to 2D co-cultures, together with a more physiological, brain-like environment of the cells.

### CRISPR–Cas9-mediated insertion of synergistic *APP* mutations to model AD

**Extended Data Fig. 6:**
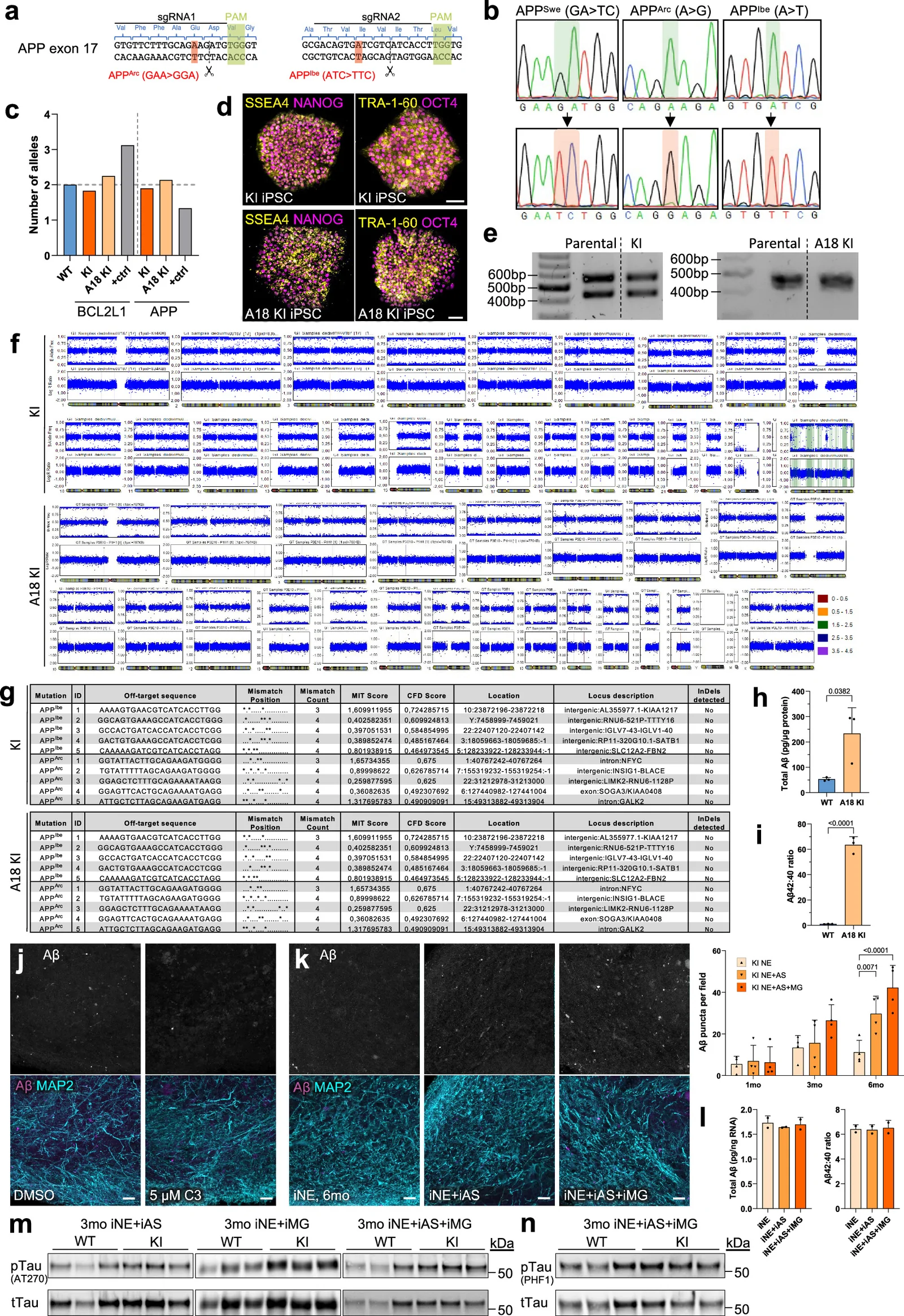
Gene editing and quality controls to introduce AD-causing APP mutations. (**A**) Overview of the edited loci in APP exon 17, sgRNAs used for editing are marked by black solid lines, associated protospacer adjacent motives (PAM) marked in green, Cas9 cut sites marked by dashed black line and scissors, mutations to be inserted are shown in red (Arctic = APP^Arc^, Iberian = APP^Ibe^). (**B**) Sanger sequencing confirming presence of the APP Swedish (APP^Swe^) mutation and successful editing for APP^Arc^ and APP^Ibe^ in line SA2. (**C**) Quantitative genomic PCRs (qgPCRs) showing normal copy number of 2 alleles at *BCL2L1* locus compared to a positive control with 3 alleles, and at the edited locus (*APP* exon 17) compared to positive control with 1 allele in both SA2 KI (KI) and A18 KI lines. (**D**) IF stainings showing expression of markers for undifferentiated state SSEA4 and NANOG (left) as well as TRA-1-60 and OCT4 (right) in both KI (top) and A18 KI (bottom) lines, scale bars 50 µm. (**E**) Fingerprinting PCR showing descent of edited KI lines from the respective parental line (SA2 left, A18 right) by same band pattern. (**F**) Molecular karyotyping of KI (top) and A18 KI (bottom) cell lines to analyze integrity of all chromosomes after editing. (**G**) Overview of most-likely off-target loci (according to crispor.tefor.net). Each locus was analyzed by Sanger sequencing and no off-target editing was found in either the KI (top) or A18 KI (bottom) line. (**H**) Immunoassay to detect Aβ in supernatants of 1-month-old A18 KI and WT 2D iNE cultures showing total Aβ levels (sum of Aβ38, 40, 42) (n = 3 experiments (biol. repl.) with 4-5 3BTMs used for media conditioning each, unpaired *t*-test). (**I**) Immunoassay as in (**H**) showing Aβ42:40 ratio (n = 3 experiments, as in (**H**), unpaired *t*-test). (**J**) IF stainings of KI 3BTMs at 6 months treated with DMSO (loading control), or 5 µM C3 (BACE-inhibitor IV) showing reduced Aβ accumulation after C3 treatment, scale bars 20 µm, quantification in Fig. 4e. (**K**) IF stainings of KI 3BTMs with different cell type combinations at 6 months, scale bars 20 µm. Quantification of Aβ puncta on the bottom showing a time course for each combination (n = 4 experiments (biol. repl.) with 1-2 3BTMs each, two-way ANOVA with Tukey’s multiple comparisons test). (**L**) Immunoassay to detect Aβ in supernatants of 1-month-old KI 3BTMs containing different cell type combinations showing total Aβ levels (left) and Aβ42:40 ratio (right) (n = 2 experiments as in (**H**)). (**M**) Western blot (WB) analysis for phospho-Tau (pTau, AT270 antibody – Thr181 epitope) and total Tau (tTau, K9JA antibody) of 3-months-old KI 3BTMs containing different cell types, quantification in Fig. 4g, h. (**N**) WB analysis as in (**M**) for pTau using the PHF1 antibody (pSer396/pSer404), quantification in Fig. 4i. Data in bar graphs are presented as mean + SD. Source data

As 3BTMs recapitulated several morphological and functional aspects of mature brain tissue, we next aimed to induce hallmarks and study the mechanisms of AD, including Aβ accumulation and neuroinflammation. Therefore, we knocked in a combination of synergistic, AD-causing mutations into SA2 WT iPSCs to induce pathogenesis while avoiding overexpression artifacts. This strategy has been successfully applied in AD mouse models; the resulting NLGF knock-in mice^39^ are broadly used in the field. To replicate the NLGF model, we started from a published line carrying the *APP* Swedish (NL) mutations^40^ and added the *APP* Arctic (G) and Iberian (F) mutations (Extended Data Fig. 6a). The resulting NLGF knock-in iPSC line was quality-controlled for successful editing using Sanger sequencing (Extended Data Fig. 6b), presence of both alleles of the edited locus and absence of *BCL2L1* trisomy (Extended Data Fig. 6c), a common pro-survival aberration in cultured stem cells. We further confirmed an undifferentiated state (Extended Data Fig. 6d), derivation from the parental cell line using fingerprinting (Extended Data Fig. 6e), absence of chromosomal aberrations using molecular karyotyping (Extended Data Fig. 6f) and absence of the most likely off-target effects (Extended Data Fig. 6g). In summary, we generated iPSCs with a triple knock-in of AD-causing *APP* mutations to study AD pathogenesis in an isogenic setting.

### AD-modeling 3BTMs replicate central AD phenotypes

**Fig. 4:**
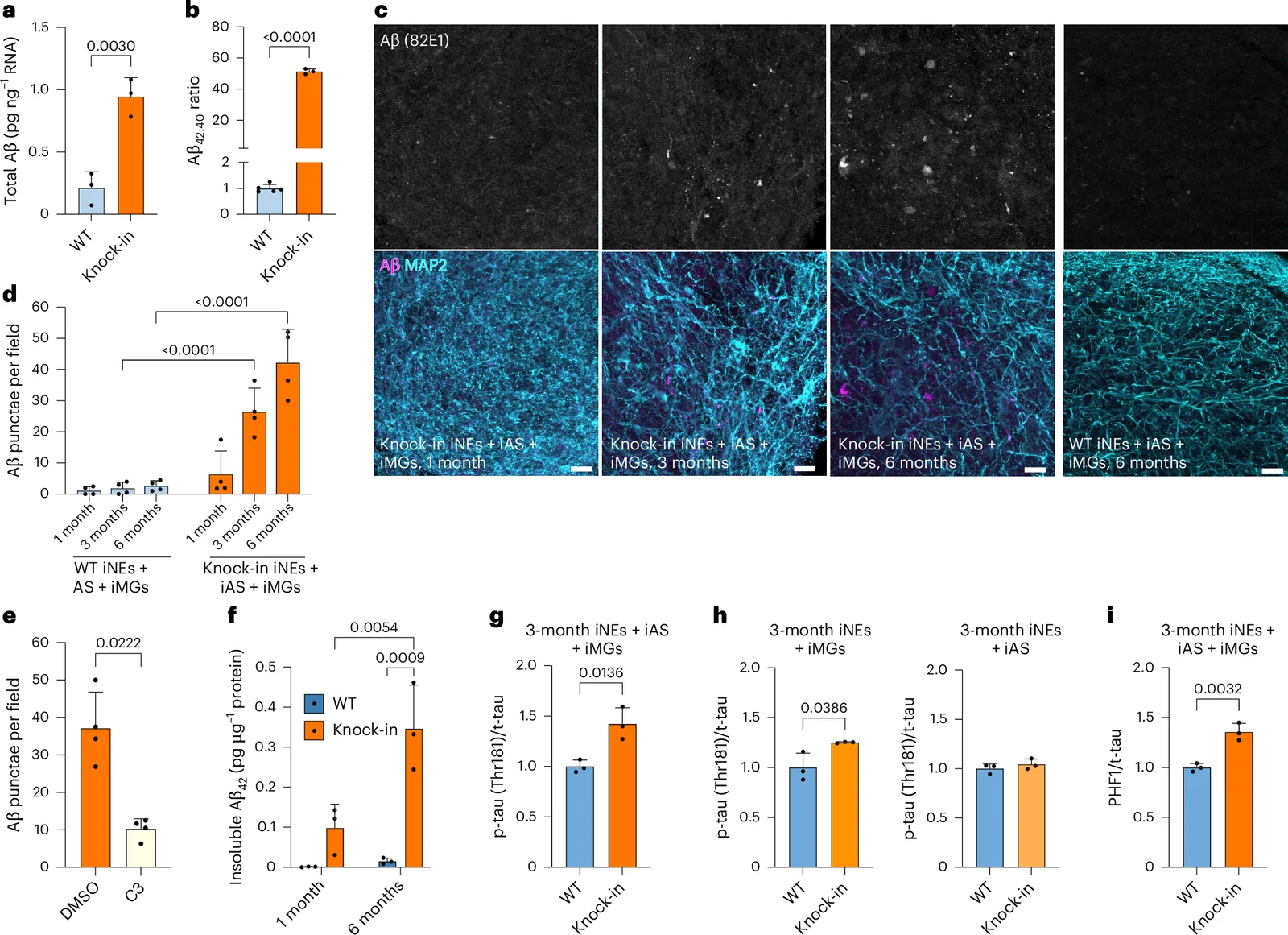
AD-modeling 3BTMs recapitulate central AD phenotypes. **a**, MSD immunoassay of 3BTM supernatants at 1 month to measure secretion of Aβ_38/40/42_ showing total Aβ levels (sum of all three species) (*n* = 3 experiments (biological replicates) with 4–5 3BTMs pooled per experiment; unpaired *t*-test). **b**, Immunoassay as in **a** showing the Aβ_42:40_ ratio (*n* = 3 (knock-in;) five (WT) experiments (biological replicates) with 4–5 3BTMs pooled per experiment, unpaired *t*-test). **c**, IF staining of knock-in 3BTMs over time and WT 3BTMs at 6 months for Aβ (82E1 antibody). **d**, Quantification of Aβ IF staining as in **c** (*n* = 4 experiments (biological replicates) with two 3BTMs per experiment and time point; two-way ANOVA with Šidák’s multiple comparisons test). **e**, Quantification of Aβ IF staining after 6-month treatment with dimethyl sulfoxide (DMSO) (as control) or 5 µM BACE inhibitor C3 (*n* = 4 experiments (biological replicates) with two 3BTMs each; unpaired *t*-test). (For IF staining, see Extended Data Fig. 6j.) **f**, Immunoassay of insoluble fractions extracted from WT and knock-in 3BTMs showing insoluble Aβ_42_ levels (*n* = 3 experiments (biological replicates) with 20–25 3BTMs pooled per experiment; two-way ANOVA followed by Tukey’s multiple comparisons test). **g**, Quantification of immunoblot (Extended Data Fig. 6m) of WT and knock-in iNE + iAS + iMG 3BTMs at 3 months detecting p-tau (AT270 antibody, Thr181 epitope) levels normalized to total tau (t-tau) levels (K9JA antibody) (*n* = 3 experiments (biological replicates) with 3–4 3BTMs pooled per experiment; unpaired *t*-test). **h**, Immunoblot analysis as in **g** of 3BTMs containing iNEs + iMGs (left) or iNEs + iAS (right) (*n* = 3 experiments (biological replicates) with 3–4 3BTMs pooled per experiment; unpaired *t*-test). **i**, Immunoblot analysis as in **g** detecting p-tau (PHF-1 antibody, Ser396/404 epitope) (for the immunoblot, see Extended Data Fig. 6n) (*n* = 3 experiments (biological replicates) with 3–4 3BTMs pooled per experiment; unpaired *t*-test). Data are presented as the mean + s.d. **c**, Scale bar: 20 µm. Source data

We differentiated WT and knock-in iPSCs into the different cell types, generated 3BTMs and analyzed AD-related phenotype formation over time. As expected from the *APP* mutations, we observed approximately fourfold increased total Aβ levels (Fig. 4a) and an ~50-fold increased Aβ_42__:40_ ratio (Fig. 4b) in knock-in cultures. Furthermore, we reproducibly found progressive Aβ accumulation in puncta and partly neurite-like structures in knock-in cultures (Fig. 4c,d). This could be prevented by application of BACE inhibitor IV (Fig. 4e and Extended Data Fig. 6j), confirming phenotype specificity and demonstrating suitability to test small-molecule drugs. Interestingly, presence of iAS and iMGs increased Aβ deposition in 3BTMs (Extended Data Fig. 6k), despite unaltered total Aβ levels and Aβ_42:40_ ratio (Extended Data Fig. 6l). As these Aβ accumulations may represent the early stages of plaque formation, we analyzed the insoluble protein fraction of 3BTMs and indeed found insoluble Aβ_42_ already in 1-month-old knock-in cultures, and increased amounts at 6 months (Fig. 4f), suggesting progressive aggregation. When analyzing p-tau levels as a downstream pathology marker of amyloid accumulation, to check if the Aβ–tau axis could be studied in 3BTMs, we found increased levels of p-tau181 as an early tauopathy marker in 3-month-old knock-in cultures (Fig. 4g and Extended Data Fig. 6m). This was most pronounced when iAS and iMGs were present, weaker if iAS were left out and not visible without iMGs (Fig. 4h and Extended Data Fig. 6m), suggesting that iMGs and iMG–iAS crosstalk may have a role in the Aβ–tau axis. Moreover, PHF1–p-tau was increased in knock-in 3BTMs, representing advanced stages of tau pathology (Fig. 4i and Extended Data Fig. 6n).

### iMGs in AD-modeling 3BTMs recapitulate disease-related changes

**Fig. 5:**
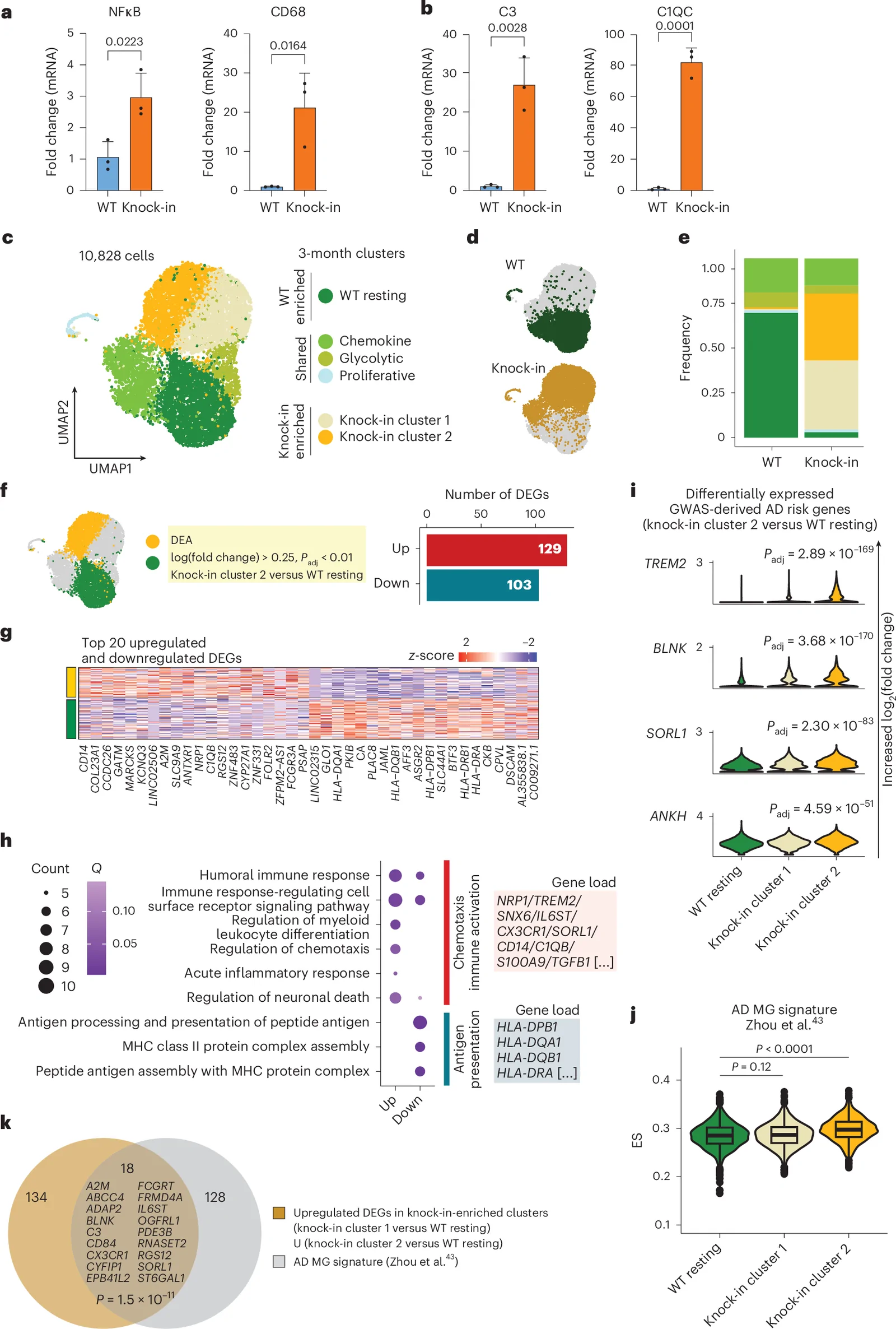
iMGsin AD-modeling 3BTMs recapitulate disease-related changes. **a**, qPCR analysis of WT and knock-in 3BTMs at 3 months for the inflammatory markers *NFκB* and *CD68* (*n* = 3 experiments (biological replicates) with 8–10 3BTMs pooled; unpaired *t*-test). **b**, qPCR analysis as in **a** for *C3* and *C1QC* (*n* = 3 experiments (biological replicates) with 8–10 3BTMs pooled; unpaired *t*-test). **c**, UMAP representation of the 3-month subset (subset 2; Extended Data Fig. 3c) of 10,828 cells and cluster annotation of WT and knock-in iMGs. **d**, UMAP representation split according to genotype. **e**, Cell state frequencies reported for knock-in and WT genotypes separately. **f**, Number of DEGs obtained by comparing knock-in cluster 2 and the WT resting clusters, including reference statistical thresholds (MAST test with Bonferroni correction for multiple comparisons). **g**, Heatmap showing normalized scaled expression of the top 20 upregulated and downregulated DEGs. **h**, ORA (GO-BP) of DEGs showing enrichment of upregulated genes in chemotaxis and immune activation, and of downregulated genes in antigen presentation. A subset of upregulated and downregulated genes contributing to the load of ORA functional terms is shown. **i**, Expression level of GWAS-defined AD risk genes^44^, which are upregulated in knock-in cluster 2 compared with WT resting, reported across WT resting, knock-in cluster 1 and knock-in cluster 2 cells (MAST test with Bonferroni correction for multiple comparisons). **j**, Violin plot reporting the AD MG signature (by Zhou et al.^43^) ES across WT resting, knock-in cluster 1 and knock-in cluster 2 cells (Wilcoxon rank-sum test). **k**, Venn diagram showing overlap of the knock-in iMG signature from this study with the human AD MG signature reported by Zhou et al.^43^ (*P* value calculated using a Fisher’s exact test). The data in the bar graphs (**a** and **b**) are presented as the mean + s.d.; the box plots show the median, 25th and 75th percentiles (box) ± 1.5 times the IQR (whiskers). Source data

**Extended Data Fig. 7:**
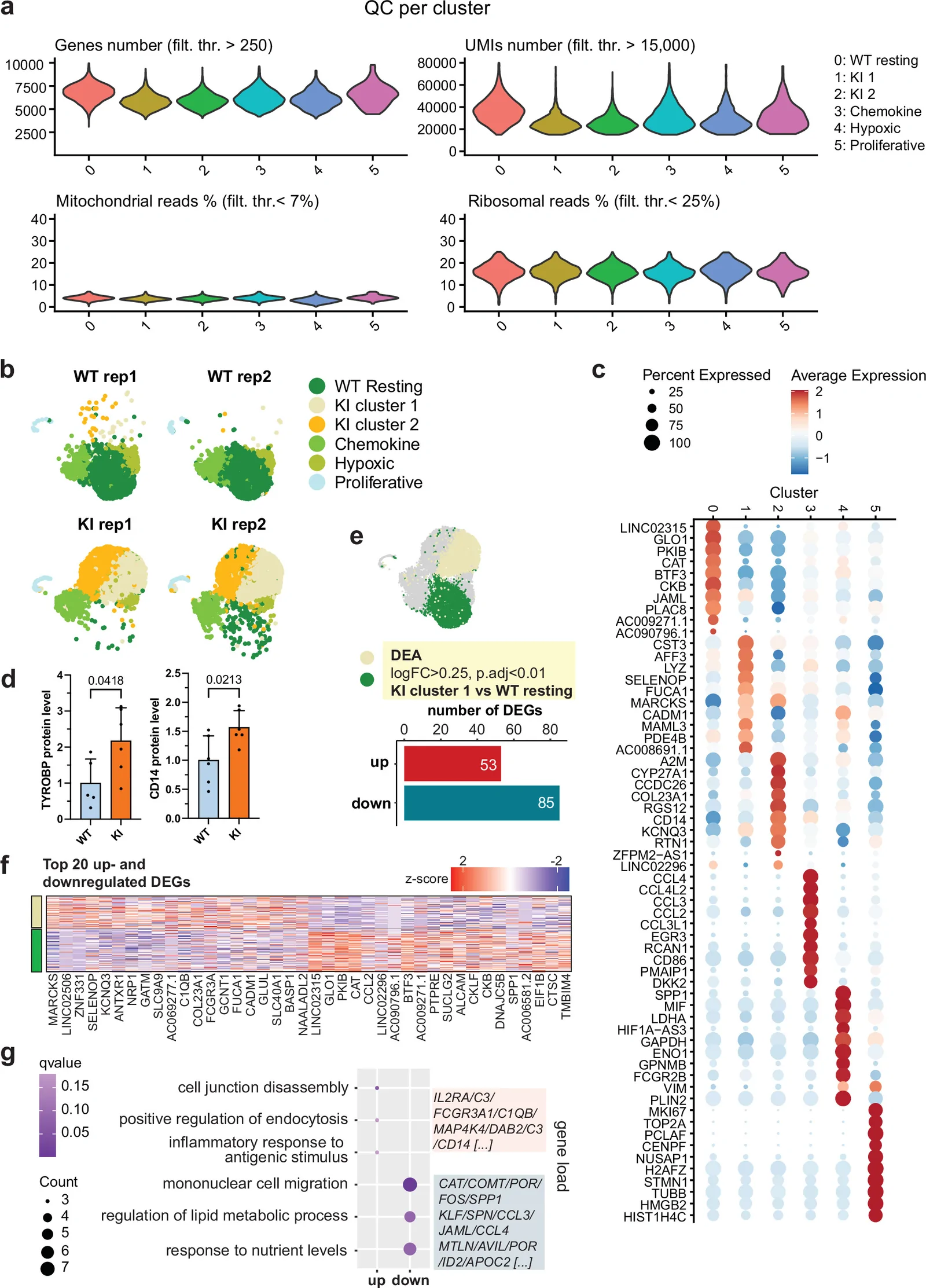
Single-cell RNA sequencing analysis of WT and KI iMG at 3 months. (**A**) Per cell quality controls across all WT and KI clusters at 3 months (Fig. 5c), including counts per cell (>250), UMIs per cell (>15,000), mitochondrial reads (<7% per cell), and ribosomal reads (<25% per cell). (**B**) UMAP reported in Fig. 5c split by biological replicates. (**c**) Top 10 marker genes (only positive, detected in >25% of cells with log_2_FC > 0.25, ordered by log_2_FC) for each cluster. (**D**) Quantification of CD14 (left) and TYROBP (right) protein levels in KI vs WT 3BTMs, data from proteomic dataset (n = 5 (A18 TYROBP) – 6 (remaining) experiments (biol. repl.) with three 3BTMs pooled for protein extraction, unpaired *t*-tests). Data are presented as mean + SD. (**E**) Number of differentially expressed (DE) genes obtained by comparing “*KI cluster 1*” and “*WT resting*” clusters including reference statistical thresholds (MAST test with Bonferroni correction for multiple comparisons). (**F**) Heatmap showing normalized scaled expression of the top 20 up- and downregulated DEGs. (**G**) Over-representation Analysis (ORA, Gene Ontology, biological process) terms enriched in the up- and down-regulated genes resulting from “*KI cluster 1*” vs “*WT resting*” clusters DE comparison. A list of interesting up- and downregulated genes contributing to the load of ORA functional terms is shown. Source data

Given the presence of Aβ and tau pathology in knock-in 3BTMs, we next studied neuroinflammatory phenotypes, facilitated by the long-term presence of mature iMGs and iAS. Using quantitative PCR (qPCR), we found increased levels of *NFκB* and *CD68* (Fig. 5a), as well as *C3* and *C1QC* (Fig. 5b), which encode proteins recently implicated in inflammatory crosstalk between iAS and iMGs in AD^41^. To gain a comprehensive understanding of AD-associated iMG alterations, we performed scRNA-seq on 3-month-old knock-in cultures in the same experiment as the WT cultures at 1 and 3 months, as described above (Extended Data Fig. 3a,b). After subsetting for iMGs from the 3-month-old WT and knock-in samples, we obtained 10,828 high-quality cells (Extended Data Fig. 7a) distributed into six clusters (Fig. 5c). Again, biological replicates showed high similarity (Extended Data Fig. 7b), confirming reproducibility. Strikingly, we found a major shift in iMG phenotypes comparing WT and knock-in samples, with knock-in iMGs forming two genotype-specific clusters (KI cluster 1 and KI cluster 2) distinct from the WT resting and the shared chemokine, glycolytic and proliferative clusters (Fig. 5d,e; top marker genes for each cluster in Extended Data Fig. 7c). We characterized the phenotype of the knock-in-specific clusters compared to the WT resting one. DEA on the knock-in cluster 2 versus WT resting identified 129 significantly upregulated genes, including *TREM2*, *CD14* and *PSAP*, all also upregulated in AD-patient-derived MGs^42,43^, and 103 downregulated genes (Fig. 5f,g). GO analysis of upregulated genes showed enrichment in immune-response-related pathways, inflammation and chemotaxis, whereas downregulated genes were enriched in antigen presentation and processing, suggesting a shift toward pro-inflammatory and dysfunctional phenotypes in knock-in iMGs (Fig. 5h). We confirmed upregulation of the key markers CD14 and TYROBP, a downstream mediator of TREM2, on protein level (Extended Data Fig. 7d). Comparing knock-in cluster 1 and WT resting yielded similar genes and terms (Extended Data Fig. 7e–g), although with lower effect sizes, suggesting an intermediate phenotype between WT resting and knock-in cluster 2. We further found a significant upregulation of different genome-wide association study (GWAS)-derived AD risk genes^44^, including *BLNK* and *SORL1*, specifically in knock-in cluster 2 (Fig. 5i), enabling studies of the AD-risk-associated effects on MG function. We next compared the knock-in iMG with published MG postmortem human AD brain expression signatures^43^. Intriguingly, we observed a significant enrichment of the patient-derived AD signature in iMG in knock-in cluster 2 (Fig. 5j), suggesting replication of the neuroinflammatory processes found in postmortem AD brains in our AD model. Upregulated genes in AD-3BTM iMG strongly overlapped with published MG signatures (Fig. 5k). Together, these findings demonstrate replication of AD-related microglial activation in 3BTMs, providing a tool for studying human-specific responses and early AD-related changes in iMGs.

### iMGs, iNEs and iAS show reproducible, AD-related changes across cell lines

**Extended Data Fig. 8:**
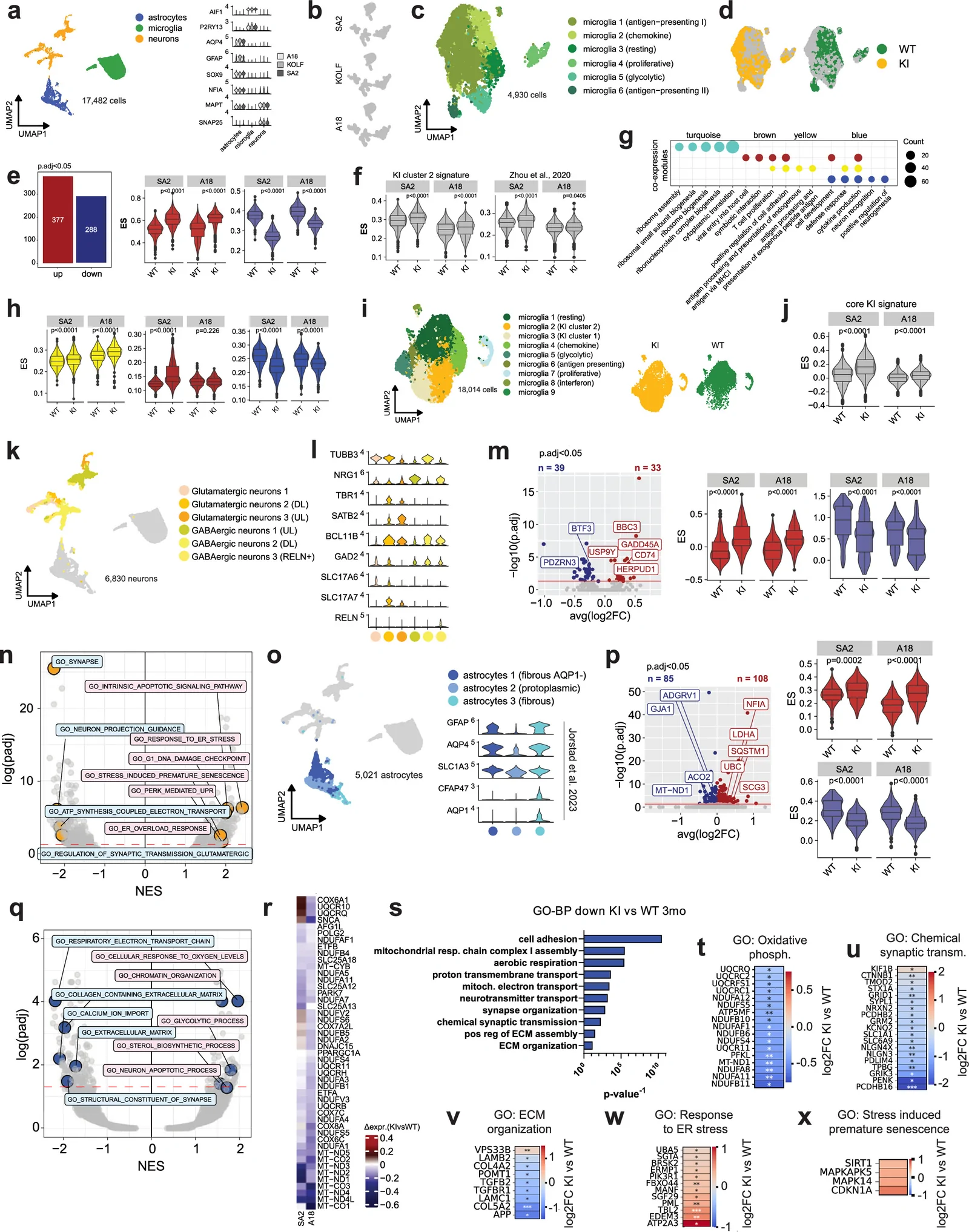
ScRNA sequencing analysis of iNE, iAS, and iMG in KI vs WT 3BTMs across independent iPSC lines. (**A**) UMAP representation of 17,482 cells and cluster annotation of iNE, iAS, and iMG extracted from all analyzed 3BTMs at 3mo. (**B**) UMAP representation as in (**A**) split by cell line. (**C**) UMAP representation of 4,930 cells in the 3mo iMG subset with cluster annotation. (**D**) UMAP as in (**C**) colored by genotype. (**E**) Left: Number of differentially expressed (DE) genes obtained by comparing WT and KI iMG within cluster 1 of the iMG subset shown in (**C**) (MAST test with Bonferroni correction for multiple comparisons). Right: Violin plots showing enrichment scores for signatures containing all upregulated (red) or downregulated (blue) genes in each line (Wilcoxon rank-sum test). (**F**) Left: Violin plots showing enrichment for the KI cluster iMG signature (derived from the dataset shown in Fig. 5) in this new dataset in iMG from lines SA2 and A18. Right: Violin plots showing enrichment of the human AD MG signature by Zhou et al.^43^ in iMG from lines SA2 and A18 (Wilcoxon rank-sum test). (**G**) High-dimensional Weighted Gene Co-expression Network Analysis (hdWGCNA) analysis of SA2 and A18 WT and KI iMG showing GO-terms associated with found co-expressed modules. (**H**) Enrichment scores for yellow, brown, and blue modules from hdWGCNA in SA2 and A18 WT and KI iMG (Wilcoxon rank-sum test). (**I**) Left: UMAP representation of the integrated dataset of SA2 and A18 WT and KI iMG in (**C**) with the SA2 WT and KI iMG in Fig. 5. Right: UMAP as on the left split by genotype. (**J**) Violin plots showing enrichment scores for the core KI signature derived from the integrated dataset in (**I**) in SA2 and A18 WT and KI iMG (Wilcoxon rank-sum test). (**K**) UMAP as in (**A**) highlighting neuronal clusters. (**L**) Violin plots showing typical marker expression across neuronal clusters. (**M**) Left: Volcano plot showing results of a DEG analysis of cluster „GABAergic neurons 2 (DL)“ between KI and WT iNE including reference statistical thresholds (MAST test with Bonferroni correction for multiple comparisons). Right: Violin plots showing enrichment scores for signatures containing all upregulated (red) or downregulated (blue) genes in each line and genotype (Wilcoxon rank-sum test). (**N**) GSEA of DEA in (**M**) showing normalized enrichment scores (NES) for different GO terms, specific relevant terms are highlighted (*fgsea* test implementation with Benjamini-Hochberg correction). (**O**) UMAP as in (**A**) highlighting iAS clusters, violin plots on the right showing expression of typical markers to confirm cluster annotations as proposed by Jorstad et al. in human brain^47^. (**P**) DEG analysis (MAST test with Bonferroni correction for multiple comparisons) and confirmation of reproducibility as in (**M**) for cluster astrocytes 2 (Wilcoxon rank-sum test). (**Q**) GSEA of DEA in (**P**) showing NES for different GO terms, specific relevant terms are highlighted (*fgsea* test implementation with Benjamini-Hochberg correction). (**R**) Heatmap showing average differential expression of genes involved in respiratory electron transport chain in KI vs WT iAS for line SA2 and A18 between KI and WT cells. (**S**) GO analysis of downregulated proteins in proteomic dataset comparing SA2 WT and KI 3BTMs at 3mo (Fisher’s Exact Test). (**T**) Heatmap showing log2FC of significantly altered proteins involved in oxidative phosphorylation in KI vs WT 3BTMs (n = 6 experiments (biol. repl.) with three 3BTMs pooled for protein extraction, unpaired *t*-tests). (**U**) Heatmap showing significantly altered proteins involved in chemical synaptic transmission in KI vs WT 3BTMs (n = 6 as in (**T**)). (**V**) Heatmap showing significantly altered proteins involved in ECM organization in KI vs WT 3BTMs (n = 6 as in (**T**)). (**W**) Heatmap showing significantly altered proteins involved in response to ER stress in KI vs WT 3BTMs (n = 6 as in (**T**)). (**X**) Heatmap showing changes in proteins involved in stress-induced premature senescence (n = 6 as in (**T**)). p-values for graphs U, V, W, X: * p < 0.05, ** p < 0.01, *** p < 0.001. Box plots show median, 25^th^ and 75^th^ percentiles (box) ± 1.5x IQR (whiskers). Source data

To confirm effects of NLGF mutations in an independent genetic background of the opposite sex, we inserted them into the A18 line. The resulting A18 knock-in iPSC line was quality-controlled for the presence of both alleles of the edited locus and absence of *BCL2L1* trisomy (Extended Data Fig. 6c), undifferentiated state (Extended Data Fig. 6d), derivation from the parental cell line using fingerprinting (Extended Data Fig. 6e), absence of chromosomal aberrations using molecular karyotyping (Extended Data Fig. 6f), and absence of most likely off-target effects (Extended Data Fig. 6g). We analyzed Aβ production and found, as in the SA2 knock-in line, a strong increase in total Aβ secretion (Extended Data Fig. 6h) as well as the Aβ_42:40_ ratio (Extended Data Fig. 6i). We then performed scRNA-seq analysis comparing A18 WT and knock-in, SA2 WT and knock-in, and KOLF WT 3BTMs in the same experiment. After quality controls (QCs), we obtained 17,482 iNEs, iAS and iMGs expressing typical markers (Extended Data Fig. 8a). Cells from all lines contributed to each cluster (Extended Data Fig. 8b), confirming similar composition of 3BTMs from the different lines also at the single-cell level.

Subclustering of iMGs from WT versus knock-in 3BTMs of the SA2 and A18 lines yielded 4,930 cells (Extended Data Fig. 8c). Within cluster 1 we found a separation according to genotype (Extended Data Fig. 8d), similar to the first dataset, and 377 upregulated and 288 downregulated genes with concordant regulation between A18 and SA2 knock-in cells (Extended Data Fig. 8e). Additionally, both knock-in lines showed higher enrichment for knock-in cluster 2 genes and the human AD MG signature by Zhou et al.^43^ (Extended Data Fig. 8f). Next, we performed high-dimensional weighted gene co-expression network analysis (hdWGCNA), which confirmed the upregulation of immune activation terms in both knock-in cell lines (yellow and brown modules; Extended Data Fig. 8g,h). Finally, we integrated the new dataset with the first dataset of SA2 iMGs and found a reproducible upregulation of the common core knock-in MG signature (Extended Data Fig. 8i,j). Overall, these data confirm the reproducibility of iMG states and responses to Aβ pathology in 3BTMs in both lines, as well as the robustness of our analysis even across scRNA-seq technologies.

Focusing on iNEs, we retrieved 6,830 cells in six clusters (Extended Data Fig. 8k) representing glutamatergic and GABAergic neurons as well as different cortical layer identities known from the human brain—mid-to-upper-layer cells with increased *SATB2* expression and deep-layer cells marked, for example, by increased *BCL11B* and *TBR1* expression. iNEs from all clusters expressed the typical markers *TUBB3* and *NRG1* (Extended Data Fig. 8l). Comparing iNEs from WT and knock-in 3BTMs with a focus on GABAergic cluster 2 as one of the most abundant clusters, we found consistent upregulation of genes related to endoplasmic reticulum (ER) stress, unfolded protein response and stress-induced premature senescence, all associated with AD^45,46^, whereas downregulated genes were enriched in synapse-associated GO terms (Extended Data Fig. 8m,n).

Lastly, we obtained 5,021 iAS in three clusters representing known subtypes, for example, protoplasmic and fibrous, as shown by marker gene comparisons (Extended Data Fig. 8o) with human brain astrocytes^47^. DEA in protoplasmic WT versus knock-in astrocytes yielded 108 upregulated and 85 downregulated genes concordant in both genetic backgrounds (Extended Data Fig. 8p). Gene set enrichment analysis (GSEA) showed a metabolic shift with upregulation of glycolysis-related genes and downregulation of oxidative-phosphorylation-related genes (Extended Data Fig. 8q,r), as described in patients with AD and models^48^, and downregulation of ECM-related terms as suggested in AD pathogenesis^49^. In addition, neuronal support functions may be impaired as indicated by downregulation of synapse-associated terms and upregulation of ‘neuron apoptotic process’ (Extended Data Fig. 8q).

To validate the scRNA-seq results, we performed proteomics of SA2 knock-in and WT 3BTMs at 3 months. GO analysis confirmed a metabolic shift with downregulation of proteins involved in oxidative phosphorylation and related terms (Extended Data Fig. 8s,t), synaptic deficits by downregulation of proteins important for chemical synaptic transmission and synapse organization (Extended Data Fig. 8s,u), and downregulation of ECM-related terms, including cell adhesion and ECM organization (Extended Data Fig. 8s,v). Additionally, we found an increase in ER stress response proteins (Extended Data Fig. 8w) together with an upward trend in proteins involved in stress-induced premature senescence (Extended Data Fig. 8x).

In summary, we could replicate the AD phenotypes observed in patients reproducibly in all three cell types of 3BTMs generated from two iPSC lines, corroborating suitability of the model to study AD-related changes and pathogenesis.

### 3BTMs enable studying AD-relevant perturbations and drug responses in human brain cells

**Fig. 6:**
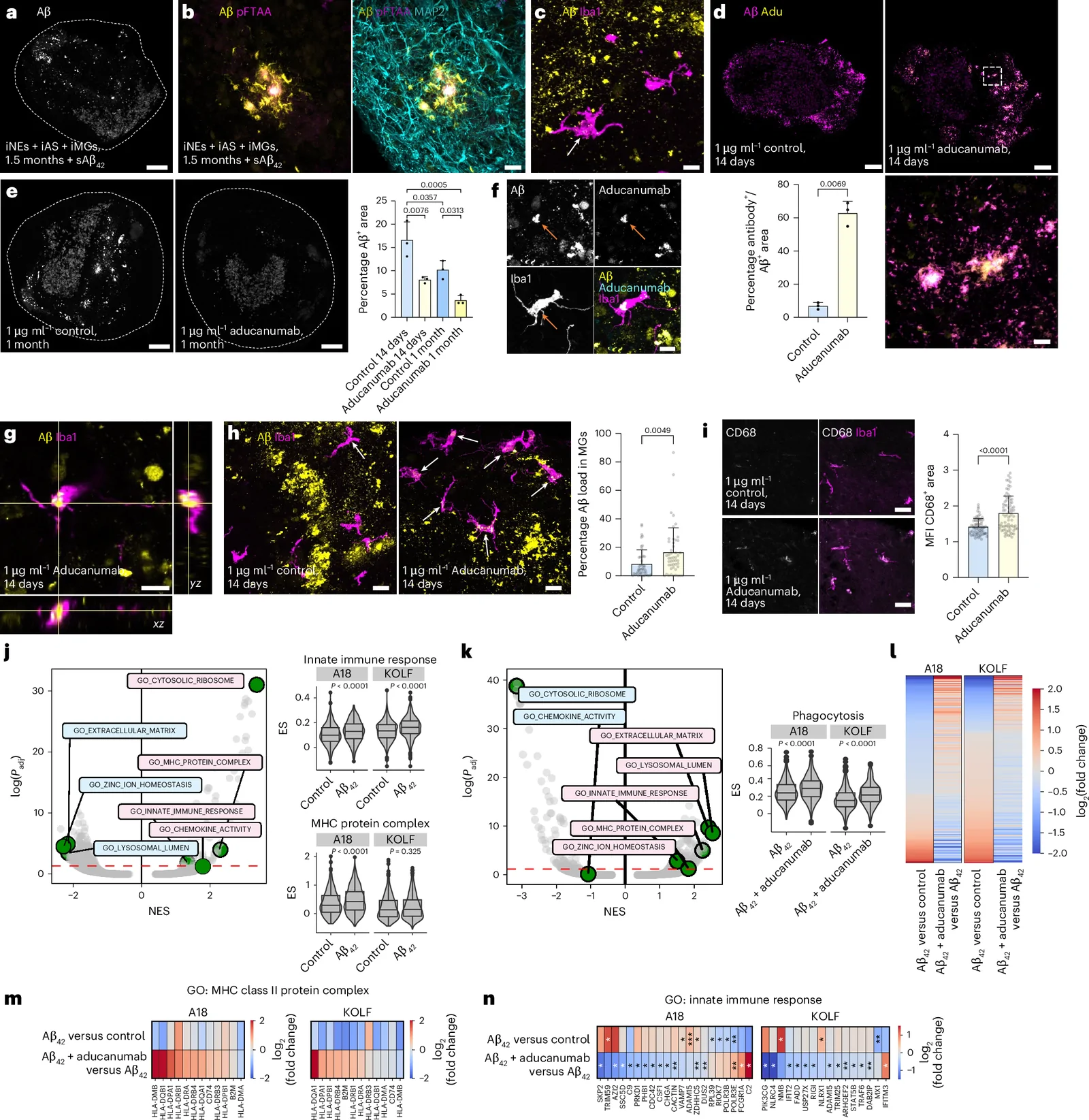
Addition of exogenous Aβ_42_ elicits plaque-like pathology and enables anti-amyloid antibody testing. **a**, Aβ IF staining of iNE + iAS + iMG 3BTMs at 1.5 months after addition of 2× 500 nM synthetic Aβ_42_ on days 8 and 10. **b** Close-up of IF staining as in **a** showing formation of a plaque-like structure with aggregated core (stained using pFTAA) and fibrillar halo (positive for Aβ). **c**, IF staining as in **a** showing colocalization of Aβ and Iba1 (arrow). **d**, IF staining of iNE + iAS + iMG 3BTMs 1 month after the addition of 2× 500 nM synthetic Aβ_42_ on days 8 and 10, and treatment with 1 µg ml^−1^ control (top left) or aducanumab (top right) antibody on days 17, 21, 24 and 28. Bottom right: Close-up showing boxed region. Bottom left: Quantification showing the percentage of Aβ^+^ area that is also antibody-positive (*n* = 3 experiments (biological replicates) with two 3BTMs each; paired *t*-test). **e**, IF staining as in **d** showing Aβ accumulation in 3BTMs treated for 1 month with control antibody (left) or aducanumab (middle). Right: Quantification of Aβ^+^ area after 14 days and 1 month (*n* = 3 experiments (biological replicates) with two 3BTMs each; one-way ANOVA with Tukey’s multiple comparisons test). **f**, IF staining showing colocalization of Aβ and aducanumab inside Iba1^+^ iMGs; the arrows show the same position in each image. **g**, Orthogonal views of the IF staining shown in **f**. **h**, IF staining for Aβ and Iba1 in 3BTMs treated as described in **d**; the arrows point to the colocalized signal. Right: Quantification showing Aβ load (Aβ^+^ area inside iMGs) (*n* = 46 (control) or 48 (aducanumab) cells (biological replicates); Mann–Whitney *U*-test). **i**, IF staining for CD68 and Iba1 in 3BTMs treated as described in **d**. Right: Quantification showing the MFI of the CD68^+^ area inside iMGs (*n* = 71 (control) or 78 (aducanumab) cells (biological replicates); Mann–Whitney *U*-test). **j**, GSEA comparing iMGs from 3BTMs treated with sAβ_42_ versus control. Left: Volcano plot showing the normalized ES (NES) and adjusted *P* values. Right: ES for signatures of the GO terms ‘innate immune response’ and ‘MHC protein complex’ across cell lines and treatments (Wilcoxon rank-sum test). **k**, GSEA comparing iMGs from 3BTMs treated with sAβ_42_ + aducanumab versus sAβ_42_ only. Left: Volcano plot showing NES and adjusted *P* values. Right: ES for the signature of the GO term ‘phagocytosis’ across cell lines and treatments (Wilcoxon rank-sum test). **l**, Heatmap showing log_2_(fold change) of proteins significantly altered after sAβ_42_ treatment (sAβ_42_ versus control) and their respective changes after aducanumab treatment (sAβ_42_ + aducanumab versus sAβ_42_) in the A18 and KOLF lines (*n* = 3 (A18) or *n* = 4 (KOLF) experiments with three 3BTMs pooled for protein extraction; unpaired *t*-test). **m**, Heatmap showing log_2_(fold change) of proteins in the GO term ‘MHC class II protein complex’ comparing sAβ_42_ versus control and sAβ42 + aducanumab versus sAβ_42_ in the A18 and KOLF lines (*n* = 3 (A18) or *n* = 4 (KOLF) experiments with three 3BTMs pooled for protein extraction; unpaired *t*-test). **n**, Heatmap showing log_2_(fold change) of proteins that are significantly changed comparing sAβ_42_ versus control or sAβ42 + aducanumab versus sAβ_42_ in the A18 and KOLF lines in the GO term ‘innate immune response’ (*n* = 3–4 as in **f**; unpaired *t*-test). *P < 0.05, **P < 0.01, ***P < 0.001. Data in the bar graphs are presented as the mean + s.d.; the box plots show the median, 25th and 75^th^ percentiles (box) ± 1.5 times the IQR (whiskers). **a**,**d**,**e**, Scale bar: 100 µm. **b**,**c**,**f**, Scale bar: 10 µm. **d**, Close-up showing boxed region, Scale bar: 10 µm. **h**,**i**, Scale bar: 20 µm. Source data

Given that 3BTMs replicated features of human brain tissue and allowed modulation of AD phenotypes, we applied the model to investigate brain cell responses to an AD-related perturbation and subsequent drug treatment. Anti-Aβ immunotherapy has been approved as the first disease-modifying AD treatment; however, its effects on brain function and cellular responses are still under investigation. Aducanumab, an approved antibody, mostly binds fibrillar Aβ^50^. Therefore, we reasoned that induction of AD-like pathology with synthetic Aβ_42_ (sAβ_42_), shown to elicit formation of fibrillar, dense-core plaques in 2D^51^, may be better suited for such a translational assay in 3BTMs than our knock-in model showing milder phenotypes representing earlier AD stages. Dense-core plaques are a key feature of later-stage AD and have a core of highly compacted Aβ fibrils with a characteristic β-sheet structure, and a surrounding halo of more soluble fibrils and oligomers. We analyzed 3BTMs 1 month after sAβ_42_ treatment (at 1.5 months of age) and indeed found even distribution of the sAβ_42_ in the cultures (Fig. 6a), its aggregation into plaque-like structures with a core positive for pFTAA, staining β-sheet structures, surrounded by fibrillar Aβ (Fig. 6b) and iMG positive for Aβ (Fig. 6c), suggesting clearance. We next treated 3BTMs with aducanumab after the addition of exogenous sAβ_42_ and analyzed Aβ accumulation and phagocytosis by iMGs, compared to cultures treated with a nontargeting control antibody. After 2 weeks of treatment, we found strong colocalization of aducanumab but not control antibody with Aβ (Fig. 6d), indicating successful penetration and target engagement. Aβ load was mildly reduced over time in control-antibody-treated cultures, suggesting clearance by iMGs. Confirming the efficacy of immunotherapy, aducanumab treatment reduced Aβ load more effectively after 2 weeks and even stronger after 1 month of treatment (Fig. 6e). Aducanumab and Aβ colocalized inside iMGs (Fig. 6f,g), which phagocytosed more Aβ in aducanumab-treated compared with control-antibody-treated cultures (Fig. 6h), confirming the importance of iMG for clearance. CD68 was increased in iMG from aducanumab-treated cultures, suggesting improved phagocytosis and activation of the cells after treatment (Fig. 6i).

**Extended Data Fig. 9:**
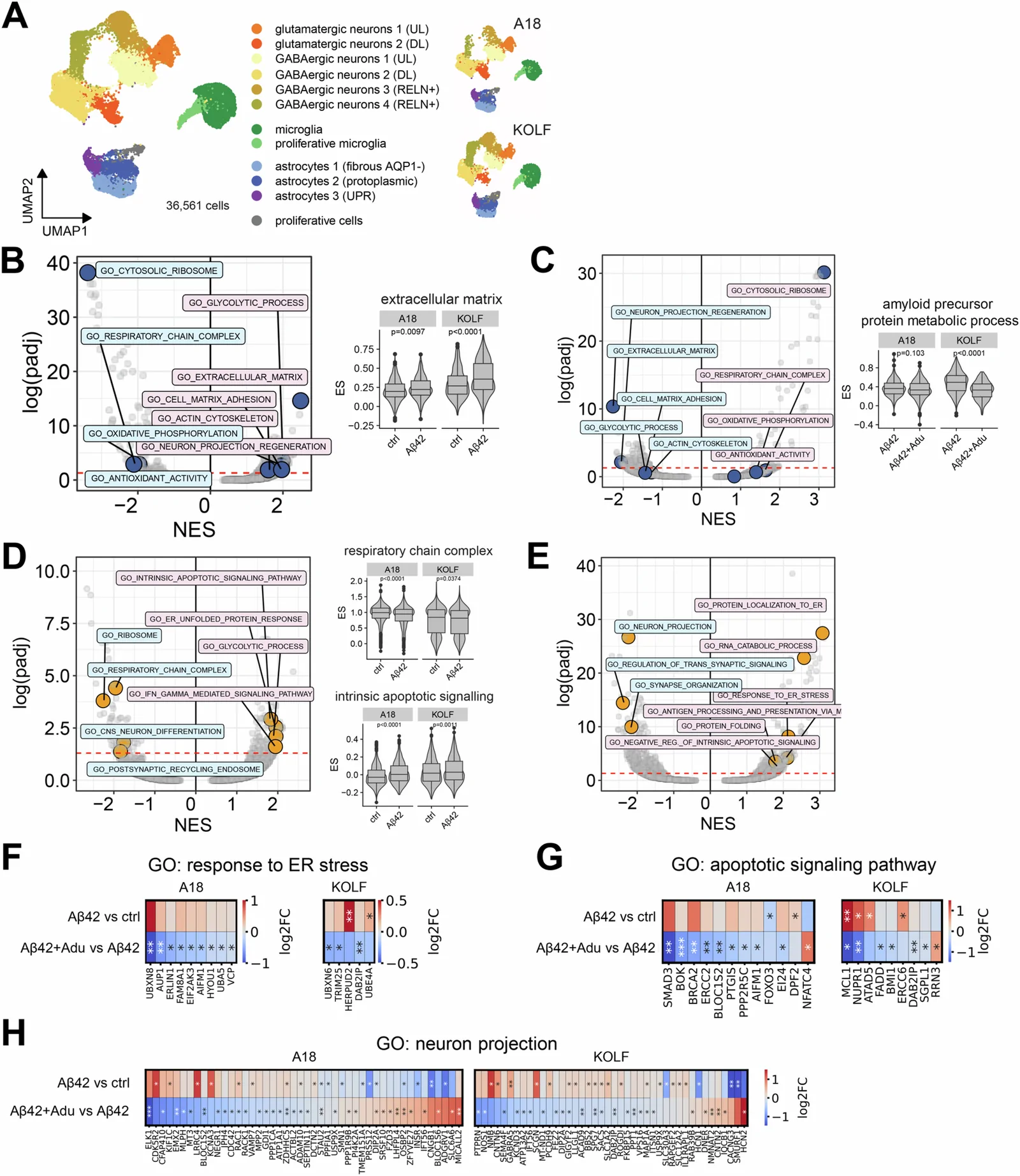
ScRNA sequencing and proteomic analysis of 3BTMs after treatment with synthetic Aβ42 and Aducanumab antibody across two independent iPSC lines. (**A**) UMAP representation of 36,561 cells and cluster annotation of iNE, iAS, and iMG extracted from 3BTMs at ~1mo after Aβ and Aducanumab treatment or controls, UMAP on the right split by cell line. (**B**) GSEA comparing iAS from 3BTMs treated with sAβ42 vs. ctrl. Left: Volcano plot showing NES and adjusted p-values (*fgsea* test implementation with Benjamini-Hochberg correction). Right: ES for GO term „extracellular matrix“across cell line and treatments (Wilcoxon rank-sum test). (**C**) GSEA comparing iAS from 3BTMs treated with sAβ42+Aducanumab vs. sAβ42 only. Left: Volcano plot showing NES and adjusted p-values (*fgsea* test implementation with Benjamini-Hochberg correction). Right: ES for signature of GO term „amyloid precursor protein metabolic process“ across cell lines and treatments (Wilcoxon rank-sum test). (**D**) GSEA comparing iNE from 3BTMs treated with sAβ42 vs. ctrl. Left: Volcano plots showing NES and adjusted p-values (*fgsea* test implementation with Benjamini-Hochberg correction). Right: ES for GO terms „respiratory chain complex“ (top) and „intrinsic apoptotic signaling pathway“ (bottom) across cell line and treatments (Wilcoxon rank-sum test). (**E**) GSEA comparing iNE from 3BTMs treated with sAβ42+Aducanumab vs. sAβ42 only (*fgsea* test implementation with Benjamini-Hochberg correction). (**F**) Heatmap showing log2FC of proteins that are significantly changed comparing sAβ42 vs ctrl and/or sAβ42+Adu vs sAβ42 in lines A18 and KOLF in GO term „response to ER stress“ (n = 3 (A18) or 4 (KOLF) experiments with three 3BTMs pooled for protein extraction, unpaired *t*-tests). (**G**) Heatmap as in (**F**) for proteins in GO term „apoptotic signaling pathway“ (n = 3-4 as in (**F**)). (**H**) Heatmap as in (**F**) for proteins in GO term „neuron projection“ (n = 3-4 as in (**F**)). p-values for graphs F, G, H: * p < 0.05, ** p < 0.01, *** p < 0.001. Box plots show median, 25^th^ and 75^th^ percentiles (box) ± 1.5x IQR (whiskers). Source data

To further investigate cell-type responses to sAβ_42_ treatment and anti-Aβ immunotherapy, we performed scRNA-seq and proteomics of 3BTMs treated with sAβ_42_ or sAβ_42_ + aducanumab, compared to untreated cultures. Experiments were performed on the A18 and KOLF lines to ensure reproducibility. scRNA-seq yielded 36,561 cells with similar representation of all cell types in both lines (Extended Data Fig. 9a). We then used functional GSEA to investigate the effects of sAβ_42_ and sAβ_42_ + aducanumab treatment in each cell type, with a focus on iMGs. Upon sAβ_42_ treatment, iMGs showed upregulation of the pathways involved in the immune response and major histocompatibility complex (MHC) protein complex formation, whereas metal ion homeostasis, ECM-related and lysosome-related terms were downregulated (Fig. 6j). Interestingly, we found a reversal of ECM, lysosomal and metal ion dysregulation by aducanumab treatment. As expected, aducanumab additionally induced further upregulation of immune-activation-related terms, including phagocytosis and MHC protein complex (Fig. 6j,k). In iAS, sAβ_42_ treatment again induced a metabolic switch from oxidative phosphorylation to glycolysis, similar to the knock-in model, and upregulation of ECM-related and cell-adhesion-related terms (Extended Data Fig. 9b). Aducanumab treatment again largely reversed these changes (Extended Data Fig. 9c). In iNEs, sAβ_42_ treatment induced changes similar to the knock-in model. These included a metabolic shift from oxidative-phosphorylation-related terms to glycolysis, and upregulation of ER-stress-related pathways and unfolded protein response, as well as apoptotic-signaling-related terms, indicating general deterioration in cell health leading to induction of cell death pathways (Extended Data Fig. 9d). Aducanumab treatment induced modulation of protein folding and ER stress response, as well as negative regulation of genes involved in apoptotic signaling pathways, suggesting induction of coping mechanisms. Synapse-associated GO terms were downregulated, potentially suggesting deterioration of cell functionality on short-term antibody treatment (Extended Data Fig. 9e). In addition, we found upregulation of MHC-related terms, as described under inflammatory conditions and in neurodegenerative diseases^52^. Proteomic analysis showed reversal of sAβ_42_-induced changes upon aducanumab treatment, suggesting an overall rescue of dysregulated pathways (Fig. 6l and Supplementary Table 1). Aducanumab treatment led to an upward trend in MHC class II complex proteins (Fig. 6m) together with overall downregulation of the innate immune response (Fig. 6n), ER stress response and apoptotic signaling (Extended Data Fig. 9f,g), suggesting positive therapeutic responses improving cell health. Like the transcriptomic analysis, we observed general downregulation of proteins in the GO term ‘neuron projection’ (Extended Data Fig. 9h). However, this downregulation was driven by proteins involved in the maintenance of mature projections and signal transduction, including ELK1, BLOC1S2 (in A18), PTPRN and KCND3 (in KOLF), whereas proteins involved in remodeling and regeneration or repair, including MICALL2, SLC6A6 (in A18), HCN2 and SMURF1 (in KOLF), were upregulated, again hinting toward induction of coping or repair mechanisms in iNEs upon aducanumab treatment.

Together, our analyses confirm that 3BTMs, upon treatment with sAβ_42_, form a dense-core Aβ plaque-like pathology. This allows applying the model to investigate therapeutic modulation of such a perturbation and enables studying drug responses in the different brain cell types, for example, in the context of anti-Aβ immunotherapy.

## Discussion

3D in vitro models offer great potential to study human cell biology and disease mechanisms. However, current brain tissue models lack features that could enhance basic and translational research applications, including high reproducibility, extended survival without proliferation and necrotic core formation, and long-term incorporation of homeostatic iMGs. In this study, we developed 3BTMs, a human in vitro model system using self-aggregation of iNE, iAS and iMG. Differentiating the cells in a defined 2D format eliminates variability arising from differential access to growth and patterning factors unavoidable in 3D, leading to exceptional reproducibility of culture formation and properties across multiple iPSC lines. 3BTMs maintain dense, mature and functional iNE and iAS networks long term, while iMGs autonomously migrate inside and develop ramifications to surveil the environment, allowing quick reaction to tissue damage.

Generating 3D models with lasting incorporation of ‘resting’, functional and mature iMGs is a longstanding challenge. Although previous studies showed incorporation of iMGs into NOs^12,13,53^, these cells commonly did not show membrane expression of the homeostatic marker P2RY12, were not ramified or declined in number within weeks^12,14–16^. 3BTMs support iMG maintenance for over 6 months, membrane expression of functional P2RY12 and homeostatic cell ramification, previously shown in 3D models only after transplantation into mouse brains or brain slices^12,54^. The improved survival and homeostasis of iMG in 3BTMs may reflect absence of a necrotic core and, therefore, more physiological environment compared to NOs, where millimeter-scale growth causes a hypoxic and necrotic core that likely also affects ‘healthy’ tissue on the culture edge. As MGs are highly sensitive to environmental changes, this may prevent adoption of a ‘resting’ state. Surrounding necrosis may also contribute to iMG death or senescence by hyperactivation, possibly explaining the rapid decline in iMG numbers in NOs. Sustained presence of mature iAS in 3BTMs may further support iMG maturation, as astrocytes promote MG ramification and maturation^55^, potentially explaining improved iMG phenotypes in 3BTMs compared to NOs, where immature iAS only arise after 2–3 months in limited numbers^17,56^. Finally, our proteomics data indicate that 3BTMs offer a more mature environment during iMG incorporation compared to NOs, possibly contributing to more mature iMG phenotypes.

Another distinctive feature of 3BTMs is absence of an artificial and endogenous development of a brain-like ECM. Many current in vitro models use Matrigel to promote long-term stability and tissue architecture^57^. However, Matrigel mainly contains laminins and collagens^58^, whereas brain ECM contains hyaluronan, proteoglycans and linker proteins^59^. As the ECM influences cell functionality^5^, these differences probably impair physiological cell maturation and function. Furthermore, Matrigel contains several lot-dependent growth factors, representing an unpredictable variable.

Another property of 3BTMs is modularity, allowing different cell and genotype combinations, unlike NOs and spheroids, where cell types inherently arise and share the genetic background as progeny of the same stem cells. This enables cell-type-specific analysis of disease risk alleles in 3BTMs, for example, ApoE4, inclusion or omission of cell types to dissect crosstalk and adaptation to different brain regions by using appropriate differentiation protocols, for example, for the midbrain to study Parkinson’s disease.

Using the mature environment provided by 3BTMs, we induced AD pathology, including Aβ accumulation and aggregation, by knocking in *APP* mutations with a synergistic effect on Aβ processing. Intriguingly, cells displayed increased tau phosphorylation using AT270 (p-tau181) and PHF-1 (p-tau396/404) antibodies, showcasing the suitability to study the Aβ–tau axis, a crucial gap in AD research. The first modular experiments suggested iMGs and crosstalk with iAS at the interface between both pathologies; as modular and reproducible systems, 3BTMs allow further investigating this crosstalk and elucidating underlying cell-type-specific mechanisms. Additionally, we observed a neuroinflammatory shift in iMG states with upregulation of AD-associated genes, including *TREM2*, *CD14*, *PSAP* and *C1QB*, as seen in postmortem AD brain MGs^42,43^. GO analysis of DEGs in all cell types revealed a metabolic switch from oxidative phosphorylation to glycolysis, altered unfolded protein response and ER stress, and stress-induced premature senescence, pathways also dysregulated in patients with AD^45,46,48,49^, suggesting that these are very early changes upon Aβ accumulation, before tau tangle formation. Overall, these data showcase opportunities to study early AD-relevant mechanisms, including cellular crosstalk in knock-in 3BTMs, especially early processes happening years before diagnosis. In line with our findings, recent studies showed AD-related crosstalk of human iNEs, iAS and iMGs in 2D^60^ and 3D^61,62^ co-culture models, highlighting the importance of studying co-cultures of disease-related cell types to understand the underlying pathomechanisms.

Because of the high reproducibility and simplicity of culture generation, 3BTMs can be efficiently upscaled and applied to drug testing, providing opportunities to overcome the scarcity of AD models replicating central disease features and being amenable for drug screening^63^. To show suitability for testing AD-relevant drugs, we confirmed the efficacy of β-secretase inhibitor IV in preventing Aβ accumulation in the knock-in model, and aducanumab-mediated clearance of aggregated Aβ in sAβ_42_-treated cultures. Aducanumab treatment induced a cellular response highly similar to findings in human iMG xenograft mice treated with lecanemab^64^ and mice treated with aducanumab^65^, showcasing the potential to investigate the effects of immunotherapy. The differential reaction to aducanumab treatment, with reversal of sAβ_42_-induced dysregulation in iMGs and iAS but not iNEs, may be explained by direct interaction of the former with the antibody, whereas the latter may be indirectly affected by removal of sAβ_42_ by glial cells, explaining the induction of coping mechanisms, including neurite regeneration and repair.

3BTMs still have several limitations: although showing several AD phenotypes, certain pathomechanisms are not yet recapitulated. Formation of dense-core plaques was achieved upon sAβ_42_ treatment, whereas aggregation in knock-in 3BTMs was only detected biochemically, indicating a milder phenotype resembling early AD. This may be because of slower generation and buildup of Aβ in knock-in 3BTMs, and its removal by degradation and media changes. Furthermore, although we observed preferential localization of iMGs in Aβ^+^ areas, we saw limited clustering around plaque-like structures in sAβ_42_-treated cultures, potentially because of the lack of neuritic plaques and coinciding neurofibrillary tangle pathology, which induce dense clustering in the brains of patients with AD^66^. In addition, Aβ clearance through the blood–brain barrier or other vascular contributions, including amyloid-related imaging abnormalities and cerebral amyloid angiopathy, also in relation to anti-amyloid immunotherapy, cannot be investigated because of the absence of blood vessels. Future vascularization studies may enable analysis of neurovascular pathomechanisms.

Altogether, 3BTMs constitute a fully stem-cell-based human cortical tissue model enabling the analysis of neurons and glia, including homeostatic MGs, investigating their interactions under physiological and pathological conditions, and applications for drug development.

## Methods

### Human participants

We analyzed brain tissue samples from two fetal donors (GW 15, male, postmortem interval ~3 days; and GW 17, male, postmortem interval ~5 days) and cortical tissue from the frontal pole of one juvenile donor (6 years, female, postmortem interval 11 h). Experiments were approved by the ethics committee of the University Hospital, LMU Munich, under project no. 23-0079 after written informed consent for collection of tissue samples and use for research purposes was given by the legal guardians in accordance with all relevant guidelines and regulations. Tissue samples were completely used and destroyed for analysis.

### Maintenance of iPSC lines

The iPSC experiments were performed in accordance with all relevant guidelines and regulations. The WT iPSC lines used in this study were: (1) 7889SA2, a single-cell clone derived from the male 7889SA line described by Paquet et al.^40^ (research resource identifier (RRID): CVCL_AD15, use was approved by the Rockefeller University Institutional Review Board after informed consent was obtained from individuals by Coriell Institute); (2) the commercially available, female iPSC line A18944 (cat. no. A18945, Thermo Fisher Scientific, RRID: CVCL_RM92); (3) KOLF2.1J cell line (cat. no. JIPSC001000, The Jackson Laboratory, RRID: CVCL_B5P3); (4) commercially available, male iPSC line GM05399 (Coriell, RRID: CVCL_7425); and (5) HPS0076: 409B2 line (RIKEN BRC Cell Bank, RRID: CVCL_K092). iPSCs were maintained as described previously^19^ on vitronectin-coated (cat. no. A14700, Thermo Fisher Scientific) plates in Essential 8 Flex Medium (E8F, cat. no. A2858501; Thermo Fisher Scientific) in a humidified incubator at 37 °C, 80–90% humidity, 5% CO_2_ and ambient O_2_ levels. Cells were kept as colonies and split every 3–4 days using PBS (cat. no. 14190094, Thermo Fisher Scientific) with 0.5 mM UltraPure EDTA (cat. no. 15575020, Thermo Fisher Scientific). iPSC lines are available from the corresponding author after filling out a material transfer agreement.

### Cortical neuron differentiation from iPSCs

Cortical neuron differentiation was performed as described by us recently^19^. Briefly, single iPSCs were split confluently into the wells of a 12-well plate coated with Geltrex (cat. no A1413302, Thermo Fisher Scientific); neural lineage was induced using dual-SMAD inhibition by adding neural differentiation medium (cat. no. 21103-049, Neurobasal) and DMEM/F12 (cat. no. 11320-074) in a 1:1 ratio with 1× B27 supplement (cat. no. 17504001), 1× NEAA (cat. no. 11140-050), 0.5× N-2 supplement (cat. no. 17502048), 1× GlutaMAX (cat. no. 35050), 1× penicillin–streptomycin (cat. no. 15140-122), 50 µM β-mercaptoethanol (cat. no. 21985-023), all Thermo Fisher Scientific, and 2.5 µg ml^−1^ insulin (cat. no. I0516, Sigma-Aldrich)) supplemented with 10 µM SB431542 (cat. no. S1067, Selleck Chemicals) and 0.25 µM LDN-193189 (cat. no. S2618, Selleck Chemical) for 10 days, WNT inhibitor XAV939 (5 µM, cat. no. S1180, Selleck Chemicals) for the first 2 days, and ROCK inhibitor (10 µM, Y27632, cat. no. S1049, Selleck Chemicals) for the first day. Neural rosettes were expanded on plates coated with poly-L-ornithin (cat. no. P4957, Sigma-Aldrich) and laminin (cat. no. 23017015, Thermo Fisher Scientific) by adding 20 ng µl^−1^ basic fibroblast growth factor (bFGF) (cat. no. 78003.2, STEMCELL Technologies) upon formation, and isolated manually using the Neural Rosette Selection Reagent (cat. no. 05832, STEMCELL Technologies) around day 20–23. Rosettes were further expanded in neural differentiation medium for 7 days, then split using Accutase (cat. no. A6964, Sigma-Aldrich) and grown for another 7 days before being frozen in neural induction medium supplemented with 10% DMSO (cat. no. D2650, Sigma-Aldrich) and 20 ng µl bFGF. After thawing, rosettes were further differentiated for 5–7 days and then used for 3BTMs on day 38–43 of differentiation.

### Astrocyte differentiation from iPSCs

Differentiation of iPSCs to astrocytes was performed according to the protocol described by Perriot et al.^20,67^, with modifications. Briefly, iPSCs were differentiated as in the cortical neuron differentiation protocol for 13–15 days, when rosettes became visible. The medium was then changed to glial precursor expansion medium (DMEM/F12 with 1× B27 supplement minus vitamin A (cat. no. 12587010; Thermo Fisher Scientific), 1× N-2 supplement, 1× GlutaMAX, 10 ng ml^−1^ bFGF, 10 ng ml epidermal growth factor (EGF) (cat. no. 100-15, Peprotech)); rosettes were manually isolated after 3 days as described in the neuron differentiation protocol. After growing confluent, rosettes were split to single cells using Accutase after which the protocol by Perriot et al.^20,67^ was followed. Glial expansion medium was applied for a total of 6–9 days, astrocyte induction medium (DMEM/F12 with 1× B27 minus vitamin A, 1× N2, 1× GlutaMAX, 10 ng ml^−1^ EGF, 10 ng ml^−1^ LIF (cat. no. 300-05, Peprotech)) for 14 days, and astrocyte medium (DMEM/F12 with 1× B27 minus vitamin A, 1× GlutaMAX, 20 ng ml^−1^ CNTF (cat. no. 450-13, Peprotech)) for 28 days, after which cells were frozen in astrocyte medium supplemented with 10% DMSO. After thawing, astrocytes were grown for 5–7 more days and then used for 3BTMs on day 64–69 of differentiation.

### MG differentiation from iPSCs

Differentiation of iPSCs to MGs was performed according to the protocol described by us^21^ and others^68^ recently. Briefly, 80–90% confluent iPSC colonies were split 1:150 to 1:200 using PBS with EDTA; hematopoiesis was induced for 12 days using the STEMDiff Hematopoietic Kit (cat. no. 05310, STEMCELL Technologies) according to the manufacturer’s instructions. Floating hematopoietic precursor cells were collected and frozen in Bambanker (cat. no. 302-14681, FujiFilm Wako). After thawing, hematopoietic precursor cells were differentiated into MGs for 12 days in DMEM/F12 supplemented with 2× insulin-transferrin-selen (cat. no. 41400-045, Thermo Fisher Scientific), 2× B27 supplement, 0.5× N-2 supplement, 1× GlutaMAX, 1× NEAA, 5 µg ml^−1^ insulin, 1× penicillin–streptomycin and 400 µM monothioglycerol (cat. no. M1753, Sigma-Aldrich), 100 ng ml^−1^ interleukin-34 (cat. no. 200-34, Peprotech), 50 ng ml^−1^ transforming growth factor beta 1 (TGF-β1) (cat. no. 100-21, Peprotech) and 25 ng ml^−1^ macrophage colony-stimulating factor (M-CSF) (cat. no. 300-25, Peprotech). MGs were used for 3BTMs on day 24 of differentiation.

### Generation of 3BTMs

To prepare 3BTMs only containing neurons, rosettes/early neurons were split into single cells using Accutase and plated into ultra-low-attachment, 96-well round-bottom plates (cat. no. CLS7007, Sigma-Aldrich) at 250,000 cells per well in 3D culture maintenance medium (Neurobasal Plus, cat. no. A3582901, Thermo Fisher Scientific, with 1× B27 Plus supplement, cat. no. A3582801, Thermo Fisher Scientific), 0.25× GlutaMAX and 1× penicillin–streptomycin). Cells were spun down for 2 min at 200*g* and cultured at 37 °C, 5% CO_2_, 80–90% humidity and ambient oxygen levels. 3D culture maintenance medium was supplemented with 10 μM DAPT (cat. no. S2215, Selleck Chemicals) for 7 days after culture generation, and with 5 μM 5-FU, cat. no. F6627, Sigma-Aldrich) and 5 μM uridine (cat. no. U3750, Sigma-Aldrich) from day 3 to day 15 after culture generation; 7–10 days after generation, aggregated 3D cultures were transferred to the wells of a 12-well plate coated with anti-adherence rinsing solution (cat. no. 07010, STEMCELL Technologies) for long-term culture with half of the medium being replaced every 3–4 days. To prepare 3BTMs containing neurons and astrocytes, cells were split as described above and plated at a neurons:astrocytes ratio of 3:1, with a total of 250,000 cells, that is, 187,500 neurons and 62,500 astrocytes. To prepare 3BTMs containing neurons, astrocytes and MGs, neurons and astrocytes were mixed and aggregated as described above. On day 15 after culture generation, when treatment with 5-FU and uridine ended, cultures were transferred to ultra-low attachment, 96-well V-bottom plates (cat. no. 651970, Greiner Bio-One) in fresh 3D culture maintenance medium supplemented with 100 ng ml^−1^ interleukin-43, 50 ng ml^−1^ TGF-β1 and 25 ng ml^−1^ M-CSF. MGs were collected after differentiation and added to the wells at 37,500 cells per 3D culture, resulting in an initial neuron:MG ratio of 5:1. Once microglia had migrated into the cultures (7–10 days), cultures were transferred back to 12-well plates coated with anti-adherence rinsing solution for long-term culture as described above, with continuous addition of interleukin-34, TGF-β1 and M-CSF. 3BTMs were generated from the 7889SA2 and A18944 lines, and from the isogenic *APP* knock-in line corresponding to 7889SA2.

### Generation of NOs

NOs were generated according to Lancaster et al.^8,69^ with minor modifications. Briefly, iPSCs were dissociated into single cells using Accutase; 9,000 cells per well were seeded to an ultra-low attachment, round-bottom 96-well plate in human stem cell medium (75% DMEM-F12, 20% knock-out serum replacement (cat. no. 10828028, Thermo Fisher Scientific), 3% FCS (cat. no. 10270106, Thermo Fisher Scientific), 1% GlutaMAX, 1% MEM-NEAA, 50 µM β-mercaptoethanol) with 4 ng ml^−1^ bFGF and 50 μM ROCK inhibitor. After 6 days, the medium was replaced for neural induction medium (DMEM-F12 with 1× N-2 supplement, 1× GlutaMAX supplement, 1× NEAA, 1 µg ml^−1^ heparin, cat. no. H3149, Sigma-Aldrich) and neuroepithelial tissues were fed every other day for 6 days. On day 12, embryoid bodies were transferred to droplets of Matrigel (cat. no. 356230, Corning) in differentiation medium (Neurobasal and DMEM/F12 in a 1:1 ratio with 1× B27 supplement minus vitamin A, 0.5× NEAA, 0.5× N-2 supplement, 1× GlutaMAX, 2.5 µg ml^−1^ insulin, 1× penicillin–streptomycin and 50 µM β-mercaptoethanol). After 4 days of stationary growth, NOs were moved to an orbital shaker at 57 r.p.m., 37 °C, 5% CO_2_ and ambient oxygen levels in differentiation medium with vitamin A. Medium was then replaced every 3–4 days. On day 90, NOs were collected and washed with PBS. Once all the liquid was removed, NOs were snap-frozen on dry ice and kept at −80 °C. NOs were generated from the GM05399 and HPS0076: 409B2 lines.

### Generation of 2D co-cultures

All cell types were differentiated from iPSCs as shown above. To generate 2D co-cultures of iNEs, iAS and iMGs, first, iNEs and iAS were split and combined exactly as described for 3BTM generation, but plated on tissue culture plates coated with poly-L-ornithin and laminin. For protein and RNA extraction, cells were plated on 6-well (900 K iNEs + 300 K iAS) and 12-well (375 K iNEs + 125 K iAS) plates, respectively. For the imaging experiments, cells were plated on acid-etched coverslips (cat. no. 630-2190, Marienfeld) coated with poly-L-ornithin and laminin (187.5 K iNEs, 62.5 K iAS). Cells were kept in the same medium as detailed for 3BTMs and MGs were added in the same way and on the same day as described.

### IF staining

For 2D IF staining, cells were plated on acid-etched coverslips as described above^19^. At the day of fixation, cells were washed once with PBS and fixed with 4% paraformaldehyde (PFA) (cat. no. 1.030.300.250, Morphisto) for 10 min at room temperature. Cells were then washed with PBS 3× for 5 min and blocked for 1 h at room temperature in blocking solution (PBS with 3% donkey serum, cat. no. D9663, Sigma-Aldrich), 0.1% Triton X-100 (cat. no. T8787, Sigma-Aldrich) and 0.02% NaN_3_ (cat. no. S2002, Sigma-Aldrich) in PBS. Primary antibodies were diluted in blocking solution (for antibody information, see Supplementary Table 3) and incubation was performed overnight at 4 °C. Coverslips were washed with PBS 3× for 5 min and then incubated with secondary antibodies (1:500 dilution) and 4′,6-diamidino-2-phenylindole (DAPI) if required (cat. no. D1306, Thermo Fisher Scientific, stored as a 5 mg ml^−1^ stock solution, 1:50,000 dilution), diluted in blocking solution for 2 h at room temperature in the dark. Cells were washed again 3× for 5 min with PBS and mounted on glass slides (cat. no. J1800AMNZ, Epredia) in Fluoromount-G (cat. no. 00-4958-02, Thermo Fisher Scientific). Coverslips were imaged with an inverted fluorescent microscope (Axio Observer Z.1, ZEISS) and images were processed using Fiji^70^.

For the 3D culture staining, cultures were washed once with PBS, fixed with cold 4% PFA for 30 min at room temperature and washed again with PBS 3× for 30 min. Cultures were then transferred into 30% sucrose (cat. no. S0389, Sigma-Aldrich) in PBS overnight at 4 °C, then transferred into optimal cutting temperature (O.C.T.) compound (Sakura Finetek Tissue-Tek O.C.T. Compound, cat. no. 1235175, Thermo Fisher Scientific) and cryosectioned into 30-µm slices. Slices were mounted onto glass slides, dried on a heat plate at 45 °C, rehydrated with H_2_O for 3 min and transferred into a humidified box. All the steps starting with blocking were performed as described above, with 3× 10 min washes in between incubations. For antibody information, see Supplementary Table 3. Terminal deoxynucleotidyl transferase dUTP nick end labeling (TUNEL) staining was performed on slides as described above using the In Situ Cell Death Detection Kit, TMR red (cat. no. 12156792910, Sigma-Aldrich) according to the manufacturer’s instructions. Hyaluronan (hyaluronic acid) was stained using biotin-coupled hyaluronic-acid-binding protein (cat.no. HKD-BC41, Hölzel Biotech) followed by avidin-fluorescein isothiocyanate-based detection (cat. no. 434411, Thermo Fisher Scientific) following the protocol outlined above. For hypoxia staining, 3BTMs were incubated with 200 µM Pimonidazole HCl (Hypoxyprobe Omni Kit, cat. no. HP3-100Kit, Hypoxyprobe), to label proteins in hypoxic cells^71^, for 2 h at standard culture conditions (ambient O_2_ levels) or at 5% O_2_ for positive controls. Staining was performed according to the manufacturer’s instructions, with primary antibody dilution of 1:50. Samples were imaged using an LSM 880 (ZEISS) inverted confocal microscope using ×10 (for overview images, EC Plan-Neofluar/0.3 Air), ×40 (for close-ups, EC Plan-Neofluar/1.30 Oil) and ×100 objectives (for synapses, alpha Plan-Apochromat/1.46 Oil) on the ZEN black software (v.10.0.4.910); images were processed and analyzed with Fiji. The images shown are representative maximum-intensity projections of *z*-stacks acquired of the 30-µm slices. All quantifications were done on unedited, raw images. TUNEL^+^ cells, Ki-67^+^ cells, NeuN/Sox9^+^ cells, DAPI^+^ cells, Aβ puncta, Aβ area after seeding and antibody treatment, and CD68 signal inside microglia were quantified by thresholding the relevant channel in the raw images and subsequent use of the ‘Analyze Particles’ function or measuring the intensity in the positive area (for the seeding and antibody treatment experiments). MG morphology was assessed using the MotiQ plug-in^72^ by cropping each MG cell manually, thresholding with the ‘Li’ algorithm and using the 3D analyzer with default settings. The ramification index was defined as the ratio of the cell surface area to the surface area of a sphere with the same volume as the respective cell, whereas the number of branches is quantified based on a skeleton of the cell created by the plug-in. Synapses were quantified using the SynQuant plugin^73^ with default settings. 3D surface renderings of synapses on/in MGs were done using Imaris v.9.4 (Oxford Instruments). First, a surface was created based on the Iba1 staining to have a representation of the MGs, then only Syn1^+^ puncta touching this surface were displayed to remove all synapses not in contact with or not inside the MGs. For IF staining, if not indicated otherwise, 1–2 cultures per experiment with 2–3 slices per 3BTM were analyzed as technical replicates and pooled for analysis.

### Mass spectrometry

#### Sample preparation

3BTMs, NOs or human FB and JB samples were washed once with PBS and dissociated in radioimmunoprecipitation assay (RIPA) buffer (50 mM HEPES, pH 7.5, cat. no. SRE0065, Sigma-Aldrich), 500 mM LiCl (cat. no. L9650, Sigma-Aldrich), 1 mM EDTA, pH 8, 1% NP-40 (cat. no. 56741, Sigma-Aldrich), 0.7% sodium deoxycholate (cat. no. 30970-25G, Sigma-Aldrich) supplemented with protease inhibitors (cat. no. 4693159001, Sigma-Aldrich) and phosphatase inhibitors (cat. no. 4906845001, Sigma-Aldrich) using ceramic beads (Precellys Ceramic kit, cat. no. 432-0293, VWR) on a Precellys Evolution homogenizer at 6,500 r.p.m. for 30 s at 4 °C. Three 3BTMs were pooled for each sample (that is, from each experiment) to increase the total protein yield; from FB sample 1 (GW 17), two pieces of tissue were extracted. 2D cultures were washed once with PBS and dissociated by direct addition of the same RIPA buffer as above, incubated for 5 min at room temperature and triturated with P1000 and P200 pipettes. Lysates were transferred to protein low-binding tubes and spun down for 20 min at 18,000*g* and 4 °C. Protein concentrations of supernatants were measured and samples were frozen at −80 °C until analysis. Then, 15 µg per sample were subjected to tryptic digestion. To this end, MgCl_2_ (10 mM) was added and DNA was digested with 25 units of benzonase (cat. no. E1014, Sigma-Aldrich) for 30 min at 37 °C. Proteins were reduced using 15 mM dithiothreitol for 30 min at 37 °C, followed by cysteine alkylation with 60 mM iodoacetamide for 30 min at 20 °C. Excess iodoacetamide was removed by adding dithiothreitol. Detergent removal and subsequent digestion with 0.2 µg LysC and 0.2 µg trypsin (Promega Corporation) was performed using the single-pot, solid-phase-enhanced sample preparation as described previously ^74^. After vacuum centrifugation, peptides were dissolved in 20 µl 0.1% formic acid (Sigma-Aldrich); peptide concentrations were estimated using the Qubit Protein Assay (Thermo Fisher Scientific).

#### Liquid chromatography–tandem MS analysis

A total of 350 ng of peptides were separated on a nanoElute nanoHPLC system (Bruker) in an in-house-packed C18 analytical column (15 cm × 75 µm ID, ReproSil-Pur 120 C18-AQ, 1.9 µm, Dr. Maisch). Peptides were separated with a binary gradient of water and acetonitrile (B) containing 0.1% formic acid at a flow rate of 300 nl min^−1^ (dataset 1 + 2: 0 min, 2% B; 2 min, 5% B; 94 min, 24% B; 112 min, 35% B; 120 min, 60% B; dataset 3: 0 min, 2% B; 2 min, 5% B; 62 min, 24% B; 72 min, 35% B; and 75 min, 60% B) and a column temperature of 50 °C.

The nanoHPLC was coupled online to a trapped ion mobility spectrometry (TIMS) with Quadrupole Time-of-Flight (TOF) pro mass spectrometer (Bruker) with a CaptiveSpray ion source (Bruker). For relative protein quantification, a data independent acquisition (DIA)–parallel accumulation serial fragmentation (PASEF) method was used^75^. Each scan cycle included one full MS scan followed by 34 windows of 26 m/z width (1 m/z overlap) covering an m/z range of 350–1,200 using two windows per PASEF ramp time of 100 ms. This resulted in a cycle time of 1.9 s. For dataset 2, a DIA–PASEF method with one TIMS full MS scan followed by 13 DIA–PASEF scans applying two isolation windows with a width of 27 m/z, covering a range of 350–1001 m/z, was used. JB samples were run in three technical replicates for datasets 1 + 2. NO samples were run in three technical replicates for dataset 1 and two technical replicates for dataset 2.

#### Data analysis

The MS raw data were analyzed with the DIA-NN software (v.1.8 for datasets 1 + 3 or v.2.0.2 for dataset 2)^76^ using a library-free search. Trypsin was defined as protease and two missed cleavages were allowed. The data were searched against one canonical protein per gene human protein database from UniProt (download: 1 March 2023, 20,603 entries for datasets 1 + 3; 9 December 2024, 20,662 entries for dataset 2), including a FASTA database with common contaminants (246 entries) from MaxQuant^77^. Oxidation of methionines and acetylation of protein N termini were defined as variable modifications, whereas carbamidomethylation of cysteines was defined as fixed modification. The precursor and fragment ion m/z ranges were limited from 350 to 1,200 (datasets 1 + 3) or 300 to 1,001 (dataset 2) and 200 to 1,700, respectively. Precursor charge states were set to a range of 2–4. Peptide and peptide fragment tolerances were optimized using DIA-NN. The match between runs option was enabled. A false discovery rate (FDR) threshold of 1% was applied for peptide and protein identifications. The generated spectral libraries included 13,552 protein isoforms, 15,198 protein groups and 222,055 precursors in 180,693 elution groups (datasets 1 + 3), and 171,283 precursors in 144,812 elution groups (dataset 2). Afterwards, all samples were searched against this spectral library for protein LFQ. Identified proteins of the contaminants database and keratins were deleted from the results tables. One sample in dataset 1 (sample 24) was excluded because it did not meet the QC standards. For the statistical analysis, only proteins that were quantified in all replicates of at least one experimental group were considered. After log_2_-transformation of LFQ intensities, missing log_2_ LFQ intensities were imputed from a normal distribution using a width of 0.3 s.d. of the data with a downshift of 1.8 using Perseus (v.1.6.14.00)^78^. PCA analysis was performed on imputed log_2_ LFQ values without scaling or centering. GO analysis (GO_BP_direct) was performed with DAVID^79,80^ using the top and bottom 400 proteins from the PCA loadings sorted according to the relevant principal component; only peptides with one unique UniProt accession number were used. Heatmaps were generated from imputed log_2_(fold changes) of LFQ values of the different samples compared with the average of the log_2_ LFQ values of 3-day-old iNE + iAS samples as the baseline. Hierarchical clustering was performed using Euclidean distance and average clustering. Synapse-associated proteins are all proteins within the GO terms ‘synapse’ (GO:0045202), ‘transmission of nerve signals’ (GO:0019226) or ‘neurotransmitter transport’ (GO:0006836) downloaded from www.uniprot.org in December 2023, which were detected in any of the samples. ECM proteins are a manually curated list of known human brain ECM proteins detected in any of the samples. Astrocyte maturation markers were curated from a list of mature alternative splicing marker genes published by Zhang et al.^27^. Corresponding proteins that were found in any of the samples were then filtered for alternative-splicing-specific expression to avoid confounding effects of expression in neurons or MG. For filtering, human expression data across brain cell types were downloaded from www.brainrnaseq.org; only proteins whose gene expression in iAS was more than 10× higher than in iNEs and more than 3× higher than in iMGs were included in the final list. Cell cycle markers are a manually curated list of known cell-cycle-associated proteins found in the dataset.

### Transmission electron microscopy

3BTMs were immersion-fixed (4% PFA and 2.5% glutaraldehyde in 0.1 M sodium cacodylate buffer, pH 7.4, Science Services) for 24 h. A standard reduced osmium tetroxide en bloc staining protocol^81^ was applied, including postfixation in 2% osmium tetroxide (EMS), 1.5% potassium ferricyanide (Sigma-Aldrich) in 0.1 M sodium cacodylate (Science Services) buffer (pH 7.4). Staining was enhanced by reaction with 1% thiocarbohydrazide (Sigma-Aldrich) for 45 min at 40 °C. 3BTMs were washed in water and incubated in 2% aqueous osmium tetroxide, washed and then further contrasted using overnight incubation in 1% aqueous uranyl acetate at 4 °C and 2 h at 50 °C. Samples were dehydrated in an ascending ethanol series and infiltrated with LX112 (LADD). Cured blocks were trimmed on an EM TRIM2 (Leica Microsystems) instrument to expose the center of the spheroid. Ultrathin sections were generated using a diamond knife (Diatome) on an UC7 microtome (Leica Microsystems) and deposited onto formvar-coated copper grids (Science Services). Sections were imaged using a JEM-1400+ (JEOL) equipped with an XF416 (TVIPS) and the EM-Menu software (TVIPS). Images analysis was performed using Fiji.

### scRNA-seq

#### Sample preparation for the 10X dataset

To obtain a single-cell suspension for scRNA-seq with a focus on iMGs (Figs. 3 and 5), 4–5 3BTMs per experiment from two independent experiments at 1 and 3 months were dissociated using the Neural Tissue Dissociation Kit P (cat. no. 130-092-628, Miltenyi Biotec) in a mix of 2.375 ml buffer X, 62.5 µl enzyme P, 10 µl enzyme A, 20 µl buffer Y and 11.25 µM actinomycin D (cat. no. A1410, Sigma-Aldrich) on a gentleMACS Octo Dissociator (Miltenyi Biotec). A custom dissociation protocol was used (+means counter-clockwise rotation, −means clockwise rotation): 4.5 min +20 r.p.m., 0.5 min −100r.p.m., 4.5 min +20 r.p.m., 0.5 min −250 r.p.m., 4.5 min +20 r.p.m., 0.5 min −300 r.p.m., 4.5 min +20 r.p.m., 0.5 min −300 r.p.m., 2 min +20 r.p.m. The dissociated cell suspension was continuously kept on ice or at 4 °C; plasticware was pretreated with 1% BSA solution (cat. no. 15260-037, Thermo Fisher Scientific) in PBS to prevent cell adhesion. Cells were filtered through a 70-µm cell strainer to remove residual clumps, spun down at 300*g* for 5 min and resuspended in 50 µl fluorescence-activated cell sorting (FACS) buffer (cat. no. 00-4222-57, Thermo Fisher Scientific). For multiplexing of biological replicates, cell suspensions were incubated for 20 min with hashtag-coupled antibodies (BioLegend), washed once with FACS buffer, pelleted for 5 min at 300*g* and resuspended in 300 µl FACS buffer. Single cells were sorted on a FACSAria Fusion (BD Biosciences) into cooled DNA low-binding tubes (Eppendorf); DAPI (250 ng ml^−1^) was added 5 min before sorting to exclude dead cells. Identical cell numbers of biological replicates (labeled with hashtag antibodies) were pooled into one collection tube and washed once in 0.04% BSA/PBS; ~16,500 cells per sample were loaded onto a 10× Chip G (10× Genomics). Single-cell gene expression and cell hashing libraries were generated on the 10X Genomics Chromium platform using the Chromium Next GEM Single Cell 3′ Reagent Kits v.3.1 according to the manufacturer’s protocol (CG000317 Rev C). Gene expression and hashtag libraries were sequenced on a NovaSeq 6000 S4 v.1.5 flow cell (Illumina).

#### Sample preparation for BD Rhapsody datasets

For the analysis of iNEs, iAS and iMGs across cell lines and after sAβ_42_/aducanumab treatment, either two (2D versus 3D comparison dataset) or three 3BTMs each from two biological replicates at the indicated age were dissociated using the Neural Tissue Dissociation Kit T (cat. no. 130-093-231, Miltenyi Biotec) in a mix of 1.9 ml buffer X, 60 µl Enzyme T, 26.6 µl buffer Y, 13.3 µl enzyme A, 5 µM actinomycin D, 10 µM triptolide (cat. no. T3652, Sigma-Aldrich) and 100 µM anisomycin (cat. no. A9789, Sigma-Aldrich) on a gentleMACS Octo Dissociator with the same custom dissociation protocol as above. Remaining clumps were then dissociated manually using a 1-ml Potter-Elvehjem PTFE pestle and glass tube. For the experiments on 2D and 3D cultures, 2D cultures were directly dissociated in dissociation buffer as above after incubation for 20 min at 37 °C; the same protocol as for 3D cultures was followed. The dissociated cell suspension was continuously kept on ice or at 4 °C; plasticware was pretreated as above. Cells were filtered and spun down as above and resuspended in 200 µl BD stain buffer (cat. no. 554656, BD Biosciences). For multiplexing of biological replicates and conditions, we used sample-tag-coupled antibodies (Human Single-Cell Multiplexing Kit, cat. no. 633781, BD Biosciences) according to the manufacturer’s instructions. Identical cell numbers of all samples were pooled into one tube and loaded onto a BD Rhapsody Express single-lane cartridge (BD Biosciences). Single-cell gene expression and cell hashing libraries were generated according to the BD Rhapsody system whole-transcriptome analysis and sample tag library preparation protocol for enhanced cell capture beads (23-24119(02) 2022-11). Gene expression and hashtag libraries were sequenced on NovaSeq 6000 and NovaSeq X instruments, using the NovaSeq 6000 S4 v.1.5 (200 bp) and NovaSeq X Series 10B (200 bp) reagent chemistries, respectively, with the 2D versus 3D comparison samples run on a NovaSeq 6000 S4 v.1.5 flow cell.

All analyses were performed in a containerized environment created by expanding the docker image available via GitHub at https://github.com/jsschrepping/r_docker to ensure reproducibility.

#### Preprocessing

For the preprocessing of the Genomics GEX and TotalSeq data (10X dataset), 10X Genomics Cell Ranger v.7.1.0 (ref. ^82^) was used with default parameters. The raw sequencing reads were aligned against the default genome reference ‘refdata-gex-GRCh38-2020-A’ using the function ‘cellranger multi’; the data were demultiplexed according to sample origin using the antibody tag sequences for the TotalSeq antibodies (BioLegend). The BD Rhapsody datasets were aligned against the same reference genome. The preprocessing of the 2D versus 3D experiment was performed using a custom pipeline based on gold standard quality checkpoints; demultiplexing was run implementing the R package deMULTIplex (v.1.0.2)^83^. For the Rhapsody dataset of iNEs, iAS and iMGs across cell lines and after sAβ/aducanumab treatment the official BD Rhapsody pipeline (v.2.1) was used for preprocessing.

#### Downstream analysis of the 10X dataset

Further downstream analysis was carried out with the Seurat pipeline (v.4.3.0.1)^84^. A starting Seurat object was created by incorporating all data and metadata available for both 1-month WT, 3-month WT and 3-month knock-in samples. A total of 16,233 cells were included in the analysis after an initial filtering to exclude lower-quality cells (more than 250 genes per cell; more than 15,000 unique molecular identifiers (UMIs) per cell); mitochondrial reads were less than 7%, with less than 25% ribosomal reads per cell (see Extended Data Fig. 3a). PCA was performed after normalization and scaling using the standard Seurat workflow. The first ten principal components were chosen to calculate UMAP embedding, adopting a clustering resolution of 0.5. Cell-type identity was attributed to each cluster based on the expression of canonical markers of MGs (TYROBP), neurons (SOX11) and astrocytes (ALDH1A1, AQP4). Only MG cells (15,479 cells) were selected for downstream analysis, as reported in Extended Data Fig. 3b. Then, two distinct subsets were analyzed (Extended Data Fig. 3b): subset 1 containing WT cells (including 1-month and 3-month cells) and subset 2 containing cells from 3-month-old 3BTMs (including knock-in and WT cells). For each subset, PCA and UMAP were calculated taking into consideration the first ten principal components and a clustering resolution of 0.3 (WT subset, 8,827 cells; Fig. 3b) and 0.4 (3-month subset, 10,828 cells; Fig. 5c), respectively. QC was performed across the obtained clusters, ensuring no QC influence on the final clustering. In most cases, cluster annotation was assigned based on the top ten markers per cluster inferred using the Seurat FindAllMarkers function (only.pos = TRUE, min.pct = 0.25, logfc.threshold = 0.25), as described in the Results section. Relative cell abundances were calculated with the propeller function from the speckle (v.1.0.0) R package^85^.

The expression of selected genes was carried out using the FeaturePlot or VlnPlot Seurat functions, reporting the normalized counts per cell for genes of interest. Further visualizations were obtained with Stacked_VlnPlot (scCustomize v.1.1.1)^86^. All DEAs were run using the Seurat FindMarkers (min.pct = 0.25, test.use = MAST with Bonferroni correction) function. Genes with *P*adj < 0.01 and log(fold change) > 0.25 (upregulated) or < −0.25 (downregulated) were defined as DEGs (Supplementary Table 2). The enrichGO function was used for the overrepresentation analyses based on the GO database (BP terms, *Q* < 0.2)^87^. Heatmaps of the top 20 upregulated and downregulated DEGs were realized with the DoHeatmap function from Seurat. Multiple signatures were used to evaluate the overrepresentation of genes related to different MG maturation stages, different in vitro models and postmortem human brain affected by AD. The gene lists used as signatures were derived from the supplementary tables of the original publication or derived after dataset reanalysis (for the Popova et al. data^13^) as mentioned in Supplementary Table 2. All reported signatures were scored using the AddModuleScore_UCell function from the R package UCell (v.2.6.2)^88^. The statistical significance in the overlap between upregulated genes in the knock-in clusters and the Zhou et al. dataset^43^ was determined using a Fisher’s exact test by implementing the testGeneOverlap^89^ and supertest^90^ functions. The expression of a list containing risk genes for AD from GWAS studies curated by Mancuso et al.^91^ was evaluated in this dataset and displayed using the Seurat VlnPlot function to visualize the expression of the AD risk genes also found among the DEGs when comparing the clusters WT Resting 3 months and knock-in cluster 2.

Monocle 3 (v.1.3.1)^92–94^ was used to determine the trajectories and pseudotime in Fig. 3i using the functions learn_graph (use_partition = FALSE) and order_cells (root_pr_nodes = ‘Y_24’). Integration between our 1-month and 3-month WT MGs and the Han et al. scRNA-seq dataset^31^ was performed using the Seurat RPCA approach (function IntegrateData, k.weight = 45) following random subsampling (*n* = 8,827) to match cell numbers in the two datasets. For the Popova et al. in vitro models dataset^13^, we first obtained separate Seurat objects and integrated them using the Seurat CCA approach (IntegrateData function with default parameters) after single-cell transform normalization. The datasets contained fetal-derived human primary MGs 2D-cultured in vitro (referred as 2D in the original dataset) or MGs incorporated into NOs (oMGs), as well as human iPSCs-derived MGs either cultured in 2D (called iMG in the original dataset) or mouse xenotransplanted (xMG). In vitro model-specific marker genes from the comparison of multiple in vitro datasets were determined with the function FindAllMarkers (only.pos = TRUE, min.pct = 0.25) by contrasting cells from each model against the others. These genes were further selected by *P*_adj _< 0.01 and fold change greater than 1 and the top 400 fold change features were used to generate each model-specific signatures (Supplementary Table 2). In all scRNA-seq analyses, *n* for DEA and signature enrichment comparisons always refers to the total number of cells per tested group, in line with common practice in the field. Specific information on the used statistical tests and multiple correction method, if applicable, is reported in the figure legends.

#### Downstream analysis of the BD Rhapsody datasets

The downstream analysis of the BD Rhapsody data was carried out similarly to the 10X dataset by implementing Seurat (v.4.3.0.1) in a containerized environment. One Seurat object per cartridge was generated and demultiplexed as described above. For each Rhapsody experiment, the Seurat objects of all cartridges were merged for QC and downstream analysis. For the 2D versus 3D dataset, two filtering steps were executed. Cells were initially filtered according to the number of UMIs and the number of genes. The thresholds were defined empirically in cartridge-specific manner, with thresholds set to 5,000 and 2,000 for the 2D cartridges and 5,056 and 8,817 for the UMIs, and 1,722 and 1,450 for genes in the 3D-specific cartridges. A second filtering step has been performed after merging each cartridge-specific object by retaining cells with mitochondrial reads less than 30%; less than 25% ribosomal reads per cell were kept for further analysis. WT 2D and 3D samples were then subset; the R package decontX (v.1.4.0)^95^ was used for ambient RNA estimation and correction. The R package harmony (v.0.1.1)^96^ was implemented for batch correction; the first 15 principal components were used to calculate UMAP, for the visualization of 12,720 high-quality cells. A clustering resolution of 0.6 was chosen and the object was then annotated based on FindAllMarkers output and expression of the canonical markers AIF1, PTPRC, AQP4, GFAP, SOX9, NFIA, MAPT and SNAP25, allowing the definition of neuronal, microglial and astrocytic compartments in both 2D and 3D samples. Seurat FindMarkers was then applied with the following arguments (ident.1 = “3D”, ident.2 = ‘2D’, test.use = ‘MAST’ with Bonferroni correction, logfc.threshold = 0) for differential expression analysis between the 3D and 2D cells of each compartment. DEGs (*P*_adj_ < 0.05 and avg_log2(fold change) < −0.25 and avg_log2(fold change) > 0.25) were then used for pathway enrichment analysis performed with the clusterProlifer (v.4.8.1) function enrichr and annotations from the GO database. The msigDBR package (v.7.5.1)^97,98^ was used to retrieve relevant gene sets for enriched GO pathways to be scored as signatures with UCell AddModuleScore_UCell function. Expression patterns and relevant enrichments were generated with the ggplot2 (v.3.5.1) R package and Seurat VlnPlot function.

For the dataset of iNEs, iAS and iMGs across cell lines and sAβ_42_/aducanumab treatments, the already QC-filtered Seurat objects output of the BD Rhapsody pipeline were also merged and underwent additional filtering for cells with less than 30% of mitochondrial reads. Three main subsets were then generated for additional investigation, including 3-month-old WT/knock-in samples, 1.5-month-old-treated samples and finally 3-month-old WT/knock-in MGs that were analyzed separately in parallel. For each subset, manual doublet detection was carried out in iterative steps and harmony was implemented for batch correction before annotation. Main cell types and cell states were annotated based on the top five positive markers per clusters ordered according to the avg_log2(fold change) and expression of canonical markers visualized with the scCustomize (v.1.1.1) Stacked_VlnPlot function. Differential expression analysis between knock-in and WT cells in clusters of interest was carried out with the Seurat FindMarkers function selecting again MAST as the test algorithm with Bonferroni correction (*P*_adj_ < 0.05 filtering threshold, latent.vars = ‘line’). In the astrocyte subset, the variable ‘day’ was also regressed to exclude any experimental batch. Similarly, the differential expression analysis comparing control, Aβ and aducanumab-treated samples, was carried out with the Seurat FindMarkers function selecting again MAST as test algorithm with Bonferroni correction (*P*_adj _< 0.05 filtering threshold, latent.vars = ‘line’ and ‘sample_univoque’). Concordance across the differential expression results across lines was then visualizes through ES calculated with AddModuleScore from Seurat. For the 3-month and treatment subset fast GSEA was executed using the fgsea R package (v.1.26.0)^99^. Statistically significant pathways (*P*_adj_ < 0.05) were then visualized with functions from the ggplot2 and ComplexHeatmap (v.2.16.0)^100^ R packages. Relevant gene sets of interest were retrieved once again through the msigdbR package and from relevant publications and scored as signatures using the AddModuleScore and AddModuleScore_UCell functions. Weighted gene correlation analysis was also used to explore functional relevance of DEGs between knock-in and WT iMGs by applying the R packages hdWGCNA (v.0.3.03)^101^ and ClusterProfiler. The analysis follows the standard network construction steps reported in the package vignette. Lastly, different subsets of MG cells across all datasets analyzed were integrated to assess overlap between molecular phenotypes using the Seurat CCA workflow with 10X dataset as a reference. The core gene set MG signature encompassing all the shared homodirectional upregulated genes in knock-in compared with WT samples across 10X and Rhapsody datasets, were then scored with the UCell AddModuleScore_Ucell function. In all scRNA-seq analyses, *n* for DEA and signature enrichment comparisons always refers to the total number of cells per tested group, in line with common practice in the field. Specific information on the statistical tests and multiple correction method used, if applicable, is reported in the legends.

### Electrophysiological measurements

3D cultures were embedded into 4% low-melting-point agarose (Thermo Fisher Scientific). The agarose block was cooled to 4 °C immediately after to accelerate solidification. The block was then rapidly glued on a vibratome specimen holder and placed in 2–4 °C cutting solution containing NMDG, KCl (2.5 mM), MgCl_2_ (2 mM), CaCl_2_ (2 mM), NaH_2_PO_4_ (1.2 mM), HEPES (10 mM), NaHCO_3_ (21 mM) and glucose (5 mM), adjusted to pH 7.2 with HCl; 250-µm sections were recovered in 36 °C cutting solution for 15 min and 40–60 min at room temperature in artificial cerebrospinal fluid containing NaCl (125 mM), KCl (2.5 mM), MgCl_2_ (2 mM), CaCl_2_ (2 mM), NaH_2_PO_4_ (1.2 mM), NaHCO_3_ (21 mM), HEPES (10 mM) and glucose (5 mM) adjusted to pH 7.2 with NaOH. Sections were continuously superfused with carbogenated artificial cerebrospinal fluid while recording at a flow rate of ~2 ml min^−1^ at 30–32 °C.

Current clamp recordings were done in perforated patch-clamp configuration. Cells were visualized with a fixed-stage microscope (BX51WI, Olympus) using a ×60 water immersion objective (LUMplan FL/N ×60, Olympus). Electrodes were prepared from borosilicate glass (cat. no. GB150-8P, Science Products) with a vertical pipette puller (PC-10, Narishige) with tip resistances between 4 and 6 MΩ. Recordings were performed with an EPC10 patch-clamp amplifier (HEKA) controlled using PatchMaster (v.2.32, HEKA); data were acquired with a micro1410 data acquisition interface and Spike 2 (v.10) (both from CED) at 25 kHz and low-pass-filtered at 2 kHz with a four-pole Bessel filter. The calculated liquid junction potential of 14.6 mV was compensated or subtracted offline (calculated with the Patcher’s Power Tools plug-in from https://www.mpibpc.mpg.de/groups/neher/index.php?page=software for IGOR Pro 6 (Wavemetrics). The pipette solution used for the recordings contained K-gluconate (147 mM), KCl (10 mM), HEPES (10 mM), EGTA (0.1 mM) and MgCl_2_ (2 mM) adjusted to pH 7.2 with KOH. The patch pipette was tip-filled and back-filled with internal solution containing the ionophore amphotericin B (200 µg ml^−1^, Sigma-Aldrich) and 0.02% tetramethylrhodamine-dextran (3,000 MW, Thermo Fisher Scientific). Access resistance (Ra) was constantly monitored during the perforation process; experiments were started after Ra had reached a steady state (~5–20 min) or the action potential amplitude was stable. Intrinsic characteristics were determined using a set of depolarizing and hyperpolarizing current injections either in perforated mode with stable action potential amplitude or membrane potential or rapidly after the membrane ruptured spontaneously.

### RNA extraction and qPCR

RNA was extracted from 7–10 3BTMs pooled per experiment to increase the yield using the NucleoSpin RNA/Protein kit (cat. no. 740933.250, Macherey-Nagel) according to the manufacturer’s instructions for cultured cells and tissues. RNA was converted into complementary DNA using the High-Capacity cDNA Reverse Transcription Kit (cat. no. 4368814, Thermo Fisher Scientific) according to the manufacturer’s instructions. qPCR was performed using the PowerTrack SYBR Green Mastermix (cat. no. A46109, Thermo Fisher Scientific) as suggested by the manufacturer. Primers were ordered from Metabion. For a list of primers, see Supplementary Table 3. To test astrocyte reactivity^102^, cells were treated with tumor necrosis factor (30 ng ml^−1^) and interleukin-1a (6 ng ml^−1^) for 24 h before sample collection.

### 2-Photon live-cell imaging

Experiments were performed using a ZEISS LSM 7 MP multi-photon microscope equipped with a Ti:Sapphire Laser (Chameleon Vision S, Coherent) and a W Plan-Apochromat ×20/1.0 DIC D = 0.17 M27 75-mm objective (ZEISS). Cultures were immobilized in a 60-mm dish (VWR) in 5-µl droplets of 5% low-melting-point agarose; 3D culture maintenance medium was carefully added. Dishes were placed on a 37 °C heat pad under the microscope and imaging was done at an excitation wavelength of 950 nm. Time lapse experiments of *z*-stacks were done at 1-µm axial step size, 512 × 512 pixel frame size, ×3 zoom for 10–15 min with 40–50 s per *z*-stack and analyzed using Fiji. For the focal laser injuries, the laser was tuned to 750 nm; a 1-frame exposure with 30% total laser power at ×40 zoom was used to damage an area of ~9-µm diameter and ~1-µm axial extent. To quantify the percentage of reacting cells in 2D co-cultures versus 3BTMs, cells were counted as ‘reacting’ if at least one process moved toward the lesion site, even if not reaching it in the time frame imaged or retracting half-way.

### Calcium imaging

3BTMs at 3 months of age were loaded for 1 h with 2.5 µM Fluo-4 AM (1:2,000 dilution from a 5 mM stock in DMSO, cat. no. ABD-20552, Biomol) in 3D culture maintenance medium with 1:2,000 Pluronic F-127 (cat. no. P3000MP, Thermo Fisher Scientific) to improve uptake. After 1 h, cultures were washed 2× with 3D culture maintenance medium, transferred to 8-well imaging slides (µ-Slide 8 Well, Ibidi) in 3D culture maintenance medium and imaged on a ZEISS LSM880 inverted confocal microscope using a water immersion objective (LD LCI Plan-Apochromat ×25/0.8) with ×2 zoom and maximum speed (acquisition time ~400 ms per frame). Acquisitions were analyzed in Fiji using the multi-measure function; results were exported to Excel and analyzed there.

### CRISPR–Cas9 gene editing

The CRISPOR online tool (http://crispor.tefor.net/)^103^ was used to design single-guide RNAs (sgRNAs) and determine the most likely off-target loci. Gene editing was performed as described previously^104,105^ with minor modifications. Briefly, sgRNAs were cloned into the MLM3636 plasmid (a gift from K. Joung, strain no. 43860, Addgene), and the pSpCas9(BB)-2A-Puro (PX459) V2.0 plasmid (a gift from F. Zhang, strain no. 62988, Addgene) was used for Cas9 expression. Single-stranded oligodeoxynucleotides were used as repair oligos, designed manually and purchased from Integrated DNA Technologies. The sequences of sgRNAs and single-stranded oligodeoxynucleotides can be found in Supplementary Table 3. For gene editing, single cells were resuspended in BTXpress electroporation solution (cat. no. 732-1285, VWR), electroporated with two pulses of 65 mV for 20 ms in a 1-mm cuvette and transferred to 10-cm plates in StemFlex Medium (cat no. A3349301, Thermo Fisher Scientific) with Revitacell supplement (1:100 dilution, cat. no. A2644501, Thermo Fisher Scientific). Cells were selected with 350 ng ml^−1^ puromycin for 3 days, starting the day after electroporation. Edited single-cell clones were analyzed using an RFLP assay, using the enzymes HpyCH4IV (cat. no. R0619S, New England Biolabs) for *APP* Arctic, and DdeI (cat. no. R0175S, New England Biolabs) for *APP* Iberian, followed by Sanger sequencing as described elsewhere^104^. Successfully edited clones were expanded and subjected to QCs. The knock-in iPSC lines are available from the authors after filling out a material transfer agreement.

### QC of edited iPSCs

Genomic integrity at the edited locus and the *BCL2L1* gene was confirmed by genomic qPCR as described previously^105,106^. Briefly, genomic DNA was extracted using the NucleoSpin Tissue Kit (cat. no. 740952.250, Macherey-Nagel) and qPCR with reverse transcription was performed using the PrimeTime Gene Expression Master Mix (cat. no. 1055772, Integrated DNA Technologies) to determine the copy numbers of the locus of interest in relation to a ‘housekeeping’ locus (*TERT* gene). The undifferentiated state of iPSCs was confirmed using immunostaining as described above with the Oct4, Tra160, SSEA4 and Nanog antibodies. To verify the origin of each edited cell line, a microvariation (‘fingerprint’) at the human D1S80 locus was analyzed using PCR. Off-target effects at the most probable loci, as predicted using CRISPOR, were excluded by PCR followed by Sanger sequencing. Primers and probes were ordered from Integrated DNA Technologies. A list of all primers and probes can be found in Supplementary Table 3. General genomic integrity was confirmed by molecular karyotyping using HumanOmni2.5Exome-8 BeadChip v1.4 (Life & Brain).

### Aβ measurements in supernatant

For each sample, the media of 3–7 parallel cultures (100 µl per culture) from the same differentiation was conditioned for 5 days, collected and pooled on ice, spun down at 300*g* at 4 °C for 5 min, transferred into protein low-binding tubes (Eppendorf), flash-frozen in liquid nitrogen and stored at −80 °C. For the analysis, supernatants were thawed on ice, centrifuged at 11,000*g* for 10 min at 4 °C and used to measure Aβ_38_, Aβ_40_ and Aβ_42_ with the V-PLEX Plus Aβ Peptide Panel 1 Kit (6E10, cat. no. K15200G, Meso Scale Discovery) according to the manufacturer’s instructions. ‘Total Aβ’ levels were calculated as the sum of the three different Aβ isoforms. Measured Aβ values were normalized to total protein or RNA levels of the cultures used for media conditioning.

### Sequential protein extraction

Protein from 3BTMs was extracted in increasingly harsh buffers, that is, (1) DEA buffer (0.2% DEA in 50 mM NaCl, pH 10), (2) RIPA buffer (20 mM TRIS-HCl, pH 7.5, 15 mM NaCl, 1 mM Na_2_ EDTA, 1 mM EGTA, 1% NP-40, 1% sodium deoxycholate, 2.5 mM pyrophosphate) and (3) guanidine-based buffer (RP1, cat. no. 740934, Macherey-Nagel) as the ‘insoluble’ fraction. To minimize protein degradation during isolation, all buffers were supplemented with protease inhibitor and all steps up to the addition of the guanidine buffer were performed on ice or at 4 °C.

For the first step, 20–25 3BTMs per sample were pooled, washed once with PBS, transferred into 100 µl DEA buffer and dissociated with ceramic beads on a Precellys Evolution homogenizer as described above. The resulting cell suspension was spun down for 10 min at 2,500*g*, the supernatant transferred to an ultracentrifuge tube (Beckman Coulter) and centrifuged at 100,000*g* for 30 min at 4 °C. The supernatant (‘DEA fraction’) was neutralized by adding 0.5 M TRIS (pH 6.75, 1:10) and flash-frozen. The pellet was resuspended in 20 µl RIPA buffer, homogenized thoroughly together with the pellet formed after the initial centrifugation step, ultracentrifuged at 100,000*g* for 30 min and the supernatant (‘RIPA fraction’) flash-frozen. The pellet was resuspended in 20 µl guanidine buffer and spun down at 100,000*g* for 30 min; the resulting supernatant (‘Guanidine fraction’ = ‘insoluble fraction’) was flash-frozen. Protein concentrations of the guanidine fraction (1:2 diluted) were measured using the Pierce BCA Protein-Assay (cat. no. 23227, Thermo Fisher Scientific). Guanidine fractions were diluted 1:10 with Diluent 35 from the MSD Human Aβ V-PLEX kit (6E10); samples were analyzed using the same kit as above and results normalized to total protein levels in the fraction.

### SDS–polyacrylamide gel electrophoresis and immunoblotting

Total protein was extracted with RIPA buffer as described for the MS analysis. Proteins were separated on hand-casted 10% Tris-Glycine gels (TGX Stain-Free FastCast Acrylamide Starter Kit, 10%, cat. no. 1610182, Bio-Rad Laboratories), at 80 V for 25 min and at 120 V for 1–2 h, and transferred to nitrocellulose membranes (0.45 µm, cat. no. 10600002, VWR) at 100 V for 1 h on ice. Membranes were blocked in 3% BSA in TBS with 0.1% Tween-20 (TBS-T), incubated in primary antibody (AT270 1:400 dilution, PHF-1 1:200 dilution, K9JA 1:10,000 dilution) in 1% BSA in TBS-T overnight at 4 °C, and washed 3× for 10 min in TBS-T. Immunoblots were then incubated in horseradish peroxidase-labeled secondary antibodies (1:10,000 dilution in blocking solution) for 1 h at room temperature, washed 3× for 10 min in TBS-T and developed with Clarity Western ECL Substrate (cat. no. 1705060, Bio-Rad Laboratories) on a Fusion Fx7 imager (Vilber). Band intensities were measured using Fiji.

### Seeding of 3BTMs with Aβ_42_ and treatment with aducanumab or control antibody

For the seeding experiments, lyophilized synthetic human Aβ_42_ (AggreSure β-Amyloid (1-42), cat. no. 72216, Hölzel Biotech) was prepared as described previously^51^. Briefly, Aβ_42_ was resuspended at 5 mM in DMSO and further diluted in PBS to a 100 µM stock solution. Resuspended peptide was incubated at 4 °C for 24 h to allow oligomerization before being frozen at −80 °C. For the experiments, oligomerized synthetic Aβ_42_ was added to the 3D culture maintenance medium at 500 nM on days 8 and 10. Human aducanumab and nonbinding control antibodies were obtained from Denali Therapeutics and added to the 3D culture maintenance medium at 1 µg ml^−1^ every 2–3 days starting on day 20.

### Statistical analysis and reproducibility

No statistical methods were used to predetermine sample size, but we chose sample sizes similar to those reported in previous publications^12,19^. ‘*n*’ denotes the number of independent experiments, that is, cultures prepared from independent batches of cell differentiations were analyzed if not indicated otherwise. In the bar graphs, if not stated otherwise, each dot represents one experiment. Experimental data were analyzed for normality and significance using Prism v.10.1 (GraphPad Software) unless stated otherwise. Multiplicity-adjusted *P* < 0.05 was considered statistically significant. Significance was always analyzed either using a two-sided Student’s *t*-test (paired or unpaired, as indicated) or a two-sided Mann–Whitney *U*-test (for non-normal distribution of values as seen by the normality test in Prism), if only two groups were compared, a one-way ANOVA if more than two groups were compared with one independent variable, a two-way ANOVA if more than two groups were compared with two independent variables. Multiple-comparison post-testing with the Tukey’s, Šidák’s or Dunn’s method was performed as recommended by Prism and as indicated. Pearson’s correlation was calculated using Prism. Statistical details are described in the figure legends. All bar graphs are shown as the mean + s.d. Box plots show the median, 25th and 75th percentiles (box) and 1.5 times the IQR (whiskers). *P < 0.05, **P < 0.01, ***P < 0.001, ****P < 0.0001. All experiments were repeated at least three times, including imaging. Generally, samples were analyzed in random order and randomly assigned to the experimental groups regarding culture age at analysis. Blinding was applied to sample processing, microscopy and image analysis.

### Reporting summary

Further information on research design is available in the Nature Portfolio Reporting Summary linked to this article.

## Online content

Any methods, additional references, Nature Portfolio reporting summaries, source data, extended data, supplementary information, acknowledgements, peer review information; details of author contributions and competing interests; and statements of data and code availability are available at https://doi.org/10.1038/s41593-026-02367-0.

## Supplementary information


Reporting Summary
Supplementary Table 1Excel file containing supplementary tables for the proteomic datasets, related to Figs. 2, 6 and Extended Data Figs. 2, 5, 8, 9.
Supplementary Table 2Excel file containing supplementary tables and information for the scRNA-seq datasets, related to Figs. 3, 5, 6 and Extended Data Figs. 3, 5, 7, 8, 9.
Supplementary Table 3Antibodies and oligonucleotides used in the study.
Supplementary Video 1MGs react to tissue damage in 3BTMs.
Supplementary Video 23BTMs show spontaneous and synchronized activity in calcium imaging.
Supplementary Video 3iMGs have close contact to and engulf synaptic material in 3BTMs.
Supplementary Video 4iMGs actively surveil their environment in 3BTMs.
Supplementary Video 52D co-cultures show spontaneous activity in calcium imaging, but no synchronization as seen in 3BTMs.


## Source data


Source Data Fig. 1Statistical source data.
Source Data Fig. 2Statistical source data.
Source Data Fig. 3Statistical source data.
Source Data Fig. 4Statistical source data.
Source Data Fig. 5Statistical source data.
Source Data Fig. 6Statistical source data.
Source Data Extended Data Fig. 1Statistical source data.
Source Data Extended Data Fig. 2Statistical source data.
Source Data Extended Data Fig. 3Statistical source data.
Source Data Extended Data Fig. 4Statistical source data.
Source Data Extended Data Fig. 5Statistical source data.
Source Data Extended Data Fig. 6Statistical source data.
Source Data Extended Data Fig. 7Statistical source data.
Source Data Extended Data Fig. 7Unprocessed immunoblots.
Source Data Extended Data Fig. 8Statistical source data.
Source Data Extended Data Fig. 9Statistical source data.


## Data Availability

The scRNA-seq profiling datasets have been deposited with the European Genome-phenome Archive (EGA) (10X dataset: EGAS50000000469; Rhapsody dataset 2D versus 3D: EGAS50000001392; Rhapsody dataset SA2 + A18 WT versus knock-in and sAβ_42_ ± aducanumab treatment: EGAS50000001397). Access to these datasets will be granted upon request to the Data Access Committee to protect the rights of human donors. The MS proteomic datasets have been deposited with the ProteomeXchange Consortium via the PRIDE partner repository with dataset ID PXD055254 (dataset 1–3BTM maturation in SA2 line in comparison to NOs and human brain samples), PXD069984 (dataset 2–reproducibility of 3BTMs across iPSC lines and comparison to 2D co-cultures) and PXD071849 (dataset 3–comparison of WT and knock-in 3BTMs). The original microscopy data reported in this article will be shared by the lead contact upon request because of large file sizes. Source data are provided with this paper.

## Code availability

All original code to analyze the scRNA-seq data, and the tool and package information to fully reproduce the analysis is publicly available via GitLab at https://gitlab.dzne.de/ag-beyer/singlecell_analysis_3btm. Any additional information required to reanalyze the data reported in this article is available from the lead contact upon reasonable request.
