# Supplementary material for: A reproducible three-dimensional model of human brain tissue to investigate physiological and disease-associated microglia phenotypes
Source: Nat Neurosci. 2026 Aug 4;29(9):2324–40. doi: 10.1038/s41593-026-02367-0 (PMC13533838; doi:10.1038/s41593-026-02367-0)
Supplement: Supplementary file 1 [file 41593_2026_2367_MOESM1_ESM.pdf]

nature portfolio

Corresponding author(s): Dominik Paquet

Last updated by author(s): Dec 11, 2025

Reporting Summary

Nature Portfolio wishes to improve the reproducibility of the work that we publish. This form provides structure for consistency and transparency in reporting. For further information on Nature Portfolio policies, see our Editorial Policies and the Editorial Policy Checklist.

Statistics

For all statistical analyses, confirm that the following items are present in the figure legend, table legend, main text, or Methods section.

n/a	Confirmed	
☐	☒	The exact sample size (*n*) for each experimental group/condition, given as a discrete number and unit of measurement
☐	☒	A statement on whether measurements were taken from distinct samples or whether the same sample was measured repeatedly
☐	☒	The statistical test(s) used AND whether they are one- or two-sided<br>*Only common tests should be described solely by name; describe more complex techniques in the Methods section.*
☒	☐	A description of all covariates tested
☐	☒	A description of any assumptions or corrections, such as tests of normality and adjustment for multiple comparisons
☐	☒	A full description of the statistical parameters including central tendency (e.g. means) or other basic estimates (e.g. regression coefficient) AND variation (e.g. standard deviation) or associated estimates of uncertainty (e.g. confidence intervals)
☐	☒	For null hypothesis testing, the test statistic (e.g. *F*, *t*, *r*) with confidence intervals, effect sizes, degrees of freedom and *P* value noted<br>*Give P values as exact values whenever suitable.*
☒	☐	For Bayesian analysis, information on the choice of priors and Markov chain Monte Carlo settings
☐	☒	For hierarchical and complex designs, identification of the appropriate level for tests and full reporting of outcomes
☐	☒	Estimates of effect sizes (e.g. Cohen's *d*, Pearson's *r*), indicating how they were calculated

Our web collection on statistics for biologists contains articles on many of the points above.

Software and code

Policy information about availability of computer code

Data collection	ZEN black software (Version 10.0.4.910); EM-Menu software (TVIPS); PatchMaster (version 2.32; HEKA); Spike 2 (version 10); FUSION FX7 software; LightCycler Software (v4.1); StepOne Software (v2.3),
Data analysis	GenomeStudio V2 (Illumina); Imaris Version 9.4; Maxquant (version 1.6.17.), Perseus (Version 1.6.14.00) and DIA-NN (Version 1.8) for analysis of proteomic data and statistics; All scRNAseq analyses were performed in a containerized environment created expanding the docker image (available at: https://github.com/jsschrepping/r_docker); All original code to analyze the scRNAseq data as well as tools/packages information to fully reproduce the analysis is publicly available on GitLab (https://gitlab.dzne.de/ag-beyer/singlecell_analysis_3btm), Genomics GEX and TotalSeq data, 10x Genomics Cell Ranger (v7.1.0); GraphPad Prism (v10.1) and Excel (2016) for statistics and figure representation; ImageJ/Fiji (version 2.14.0/1.54f) for image processing and quantification; R packages: speckle (v1.0.0), Seurat (v4.3.0.1), “Stacked_VlnPlot” (scCustomize v1.1.1), UCell (v.2.6.2), Monocle 3 (v1.3.1), deMULTIplex (v1.0.2), decontX (v1.4.0), harmony (v0.1.1), clusterProlifer (v4.8.1), msigDBR (v7.5.1), ggplot2 (v3.5.1), fgsea (v1.26.0), ComplexHeatmap (v2.16.0), hdWGCNA (v0.3.03); BD Rhapsody pipeline (v2.1),

For manuscripts utilizing custom algorithms or software that are central to the research but not yet described in published literature, software must be made available to editors and reviewers. We strongly encourage code deposition in a community repository (e.g. GitHub). See the Nature Portfolio guidelines for submitting code & software for further information.

Data

Policy information about availability of data

All manuscripts must include a data availability statement. This statement should provide the following information, where applicable:

- Accession codes, unique identifiers, or web links for publicly available datasets
- A description of any restrictions on data availability
- For clinical datasets or third party data, please ensure that the statement adheres to our policy

The scRNA-seq profiling datasets have been deposited to the European Genome-phenome Archive (EGA) (10X dataset: EGAS50000000469, Rhapsody dataset 2D vs 3D: EGAS50000001392, Rhapsody dataset SA2+A18 WT vs KI and sAβ42±Aducanumab treatment: EGAS50000001397). Access to these datasets will be granted upon reasonable request to the Data Access Committee (DAC) to protect rights of human donors. The mass spectrometry proteomic datasets have been deposited to the ProteomeXchange Consortium via the PRIDE partner repository with the dataset identifier PXD055254 (Dataset 1 – 3BTM maturation in SA2 line in comparison to NO and human brain samples), PXD069984 (Dataset 2 – Reproducibility of 3BTMs across iPSC lines and comparison to 2D co-cultures), and PXD071849 (Dataset 3 – Comparison of WT and KI 3BTMs). Original western blot images and microscopy data reported in this paper will be shared by the lead contact upon request due to large file sizes. All other source data are provided with the paper.

Research involving human participants, their data, or biological material

Policy information about studies with human participants or human data. See also policy information about sex, gender (identity/presentation), and sexual orientation and race, ethnicity and racism.

Reporting on sex and gender	Gender of brain sample donors and iPSC line donors is described in methods.
Reporting on race, ethnicity, or other socially relevant groupings	not relevant for the study, therefore not determined and not reported.
Population characteristics	N/A
Recruitment	N/A
Ethics oversight	Experiments were approved by the ethics committee of the University Hospital, LMU Munich, and informed consent for collection of tissue samples and use for research purposes was given by the legal guardians

Note that full information on the approval of the study protocol must also be provided in the manuscript.

Field-specific reporting

Please select the one below that is the best fit for your research. If you are not sure, read the appropriate sections before making your selection.

☒ Life sciences ☐ Behavioural & social sciences ☐ Ecological, evolutionary & environmental sciences

For a reference copy of the document with all sections, see nature.com/documents/nr-reporting-summary-flat.pdf

Life sciences study design

All studies must disclose on these points even when the disclosure is negative.

Sample size	Sample sizes were determined based on results obtained in previous proteomic, transcriptomic and immunohistochemical studies by the authors or found in the literature.
Data exclusions	1 of 6 samples of the 6mo NE+AS+MG group of proteomic dataset 1 was excluded in the mass spec analysis as it did not meet quality control standards.
Replication	iPSC experiments were replicated with at least 3 biological replicates, model characterization was done in 3 independent iPSC lines, AD-related experiments were performed in 2 independent, isogenic pairs of iPSC lines (except proteomic analysis). All experiments were replicated at least 3 times, including imaging. All attempts at replication were successful.
Randomization	Samples were generally analyzed in random order and randomly assigned to experimental groups regarding culture age at analysis.
Blinding	Blinding was applied to sample processing, microscopy, and image analysis.

Reporting for specific materials, systems and methods

We require information from authors about some types of materials, experimental systems and methods used in many studies. Here, indicate whether each material, system or method listed is relevant to your study. If you are not sure if a list item applies to your research, read the appropriate section before selecting a response.

Materials & experimental systems

n/a	Involved in the study
☐	☒ Antibodies
☐	☒ Eukaryotic cell lines
☒	☐ Palaeontology and archaeology
☒	☐ Animals and other organisms
☒	☐ Clinical data
☒	☐ Dual use research of concern
☒	☐ Plants

Methods

n/a	Involved in the study
☒	☐ ChIP-seq
☒	☐ Flow cytometry
☒	☐ MRI-based neuroimaging

Antibodies

Antibodies used

82E1 (Aβ);Mouse;IBL/Tecan;Cat#JP10323;RRID: AB_1630806; 1:100
Aquaporin 4;Rabbit;Alomone Labs ;Cat#AQP-004;RRID: AB_2039734;1:200
AT270 (phospho-Tau Thr181);Mouse;Thermo Fisher ;Cat#MN1050;RRID: AB_223651;1:400
CD68;Mouse;Thermo Fisher;Cat#MA5-13324;RRID: AB_10987212;1:100
Cleaved Caspase 3;Rabbit;Cell Signaling;Cat#9664;RRID: AB_2070042;1:500
CTIP2;Rat;Abcam;Cat#ab18465;RRID: AB_2064130;1:300
GFAP;Guinea pig;Synaptic Systems;Cat#173004;RRID: AB_10641162;1:500
GLAST (ACSA-1);Mouse;Miltenyi Biotec;Cat#130-095-822;RRID: AB_10829302;1:200
GLT-1;Guinea pig;Sigma-Aldrich;Cat#AB1783;RRID: AB_90949; 1:500
Glypican-4 Rabbit; Proteintech Cat#13048-1-AP; RRID: AB_10640157,1:800
Iba1;Guinea pig;Synaptic Systems;Cat#234 308;RRID: AB_2924932; 1:500
Iba1;Rabbit ;Thermo-Fisher;Cat#PA5-27436;RRID: AB_2544912; 1:500
K9JA (total Tau) (1:1000 for stainings, 1:10.000 for western blot);Rabbit;Dako;Cat#A0024;RRID: N/A
Ki67;Rabbit;Abcam;Cat#ab15580;RRID: AB_443209; 1:500
MAP2;Chicken;Millipore;Cat#AB15452;RRID: AB_11211337; 1:1000
NANOG;Rabbit;Cell Signaling;Cat#4903;RRID: AB_10559205; 1:500
NeuN;Mouse;Sigma-Aldrich;Cat#MAB377;RRID: AB_2298772; 1:500
Oct4;Rabbit;Stemgent;Cat#S090023;RRID:N/A; 1:500
P2RY12;Rabbit;Sigma-Aldrich;Cat#HPA014518;RRID: AB_2669027; 1:1500
PHF1 (phospho-Tau Ser396/404);Mouse;Gift from Peter Davies' lab; 1:200
PSD95;Mouse;Thermo fisher;Cat#MA1-046;RRID: AB_2092361; 1:100
PU.1;Rabbit;Cell Signaling;Cat#2258;RRID: AB_2186909; 1:500
S100β;Mouse;Sigma-Aldrich;Cat#S2532;RRID: AB_477499; 1:500
SATB2;Mouse;Abcam;Cat#ab51502;RRID: AB_882455; 1:100
Sox9;Rabbit;Sigma-Aldrich;Cat#AB5535;RRID: AB_2239761; 1:500
SSEA4;Mouse;Abcam;Cat#ab16287;RRID: AB_778073; 1:500
Synapsin-1;Rabbit;Cell Signaling;Cat#5297;RRID: AB_2616578; 1:500
TotalSeq-B0251 anti-human Hashtag 1 Antibody ;Mouse;Biolegend;Cat#394631;RRID: AB_2814347; 1:33
TotalSeq-B0251 anti-human Hashtag 2 Antibody ;Mouse;Biolegend;Cat#394633;RRID: AB_2814348; 1:33
TotalSeq-B0251 anti-human Hashtag 3 Antibody;Mouse;Biolegend;Cat#394635;RRID: AB_2814349; 1:33
TRA-1-60;Mouse;Millipore;Cat#MAB4360;RRID: AB_21191831:500
TuJ1 (β3-Tubulin);Rabbit;Covance;Cat#MRB-435P;RRID: AB_663339; 1:500
Anti-chicken Alexa Fluor Plus 488;Goat;Thermo Fisher;Cat#A32931;RRID: AB_2762843;1:500
Anti-chicken Alexa Fluor Plus 647;Goat;Thermo Fisher;Cat#A32933;RRID: AB_2762845; 1:500
Anti-mouse Alexa Fluor Plus 488;Donkey;Thermo Fisher;Cat#A32766;RRID: AB_2762823; 1:500
Anti-mouse Alexa Fluor 647;Donkey;Thermo Fisher;Cat#A31571;RRID: AB_162542; 1:500
Anti-rabbit Alexa Fluor 568;Donkey;Thermo Fisher;Cat#A10042;RRID: AB_2534017; 1:500
Anti-rabbit Alexa Fluor 647;Goat;Thermo Fisher;Cat#A27040;RRID: AB_2536101; 1:500
Anti-rat Alexa Fluor 488;Donkey;Thermo Fisher;Cat#A21208;RRID: AB_2535794; 1:500
Anti-rat Alexa Fluor 647;Goat;Thermo Fisher;Cat#A21247;RRID: AB_141778; 1:500
Anti-guinea pig Alexa Fluor 488;Goat;Thermo Fisher;Cat#A11073;RRID: AB_2534117; 1:500
Anti-guinea pig Alexa Fluor 568;Goat;Thermo Fisher;Cat#A11075;RRID: AB_2534119; 1:500
Anti-guinea pig Alexa Fluor 647;Goat;Thermo Fisher;Cat#A21450;RRID: AB_2535867; 1:500
Peroxidase AffiniPure Anti-Rabbit IgG (H+L);Donkey;Jackson Immunoresearch;Cat#711-035-152;RRID: AB_10015282; 1:1000
Peroxidase AffiniPure Anti-Mouse IgG (H+L);Donkey;Jackson Immunoresearch;Cat#715-035-151;RRID: AB_2340771; 1:1000

Validation

All Antibodies are commercially available and were used according to the manufacturer's instructions.

Eukaryotic cell lines

Policy information about cell lines and Sex and Gender in Research

Cell line source(s)

A18944 iPSCs: purchased from Thermofisher (Cat. No. A18945), female
7899SA2 WT iPSCs: obtained from NYSCF repository, ID CO0002-01-SV-003, male

	7889SA2 Swe/Swe iPSCs: obtained from NYSCF repository, CO0002-01-CS-004 / BN0013, male<br>GM05399 iPSCs: obtained from Coriell Repository, RRID: CVCL_7425, male<br>KOLF2.1J iPSCs: obtained from The Jackson Laboratory, Cat# JIPSC001000, RRID:CVCL_B5P3, male<br>HPS0076 : 409B2 iPSCs: RIKEN BRC cell bank, RRID:CVCL_K092; female
Authentication	Authenticated by supplier and confirmed by fingerprinting and genotyping where appropriate.
Mycoplasma contamination	The lines were tested and confirmed negative for mycoplasma contamination.
Commonly misidentified lines<br>(See ICLAC register)	N/A

Plants

Seed stocks	*Report on the source of all seed stocks or other plant material used. If applicable, state the seed stock centre and catalogue number. If plant specimens were collected from the field, describe the collection location, date and sampling procedures.*
Novel plant genotypes	*Describe the methods by which all novel plant genotypes were produced. This includes those generated by transgenic approaches, gene editing, chemical/radiation-based mutagenesis and hybridization. For transgenic lines, describe the transformation method, the number of independent lines analyzed and the generation upon which experiments were performed. For gene-edited lines, describe the editor used, the endogenous sequence targeted for editing, the targeting guide RNA sequence (if applicable) and how the editor was applied.*
Authentication	*Describe any authentication procedures for each seed stock used or novel genotype generated. Describe any experiments used to assess the effect of a mutation and, where applicable, how potential secondary effects (e.g. second site T-DNA insertions, mosiacism, off-target gene editing) were examined.*
