# Supplementary material for: A reproducible three-dimensional model of human brain tissue to investigate physiological and disease-associated microglia phenotypes
Source: Nat Neurosci. 2026 Aug 4;29(9):2324–40. doi: 10.1038/s41593-026-02367-0 (PMC13533838; doi:10.1038/s41593-026-02367-0)

Extended Data Fig. 7N

3mo iNE+iAS, pTau (AT270)

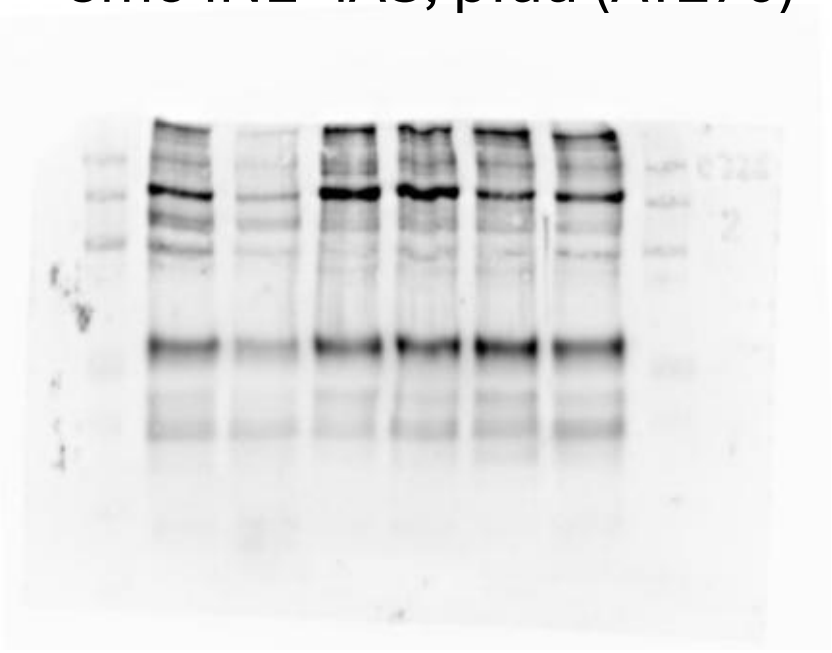

3mo iNE+iAS, total Tau

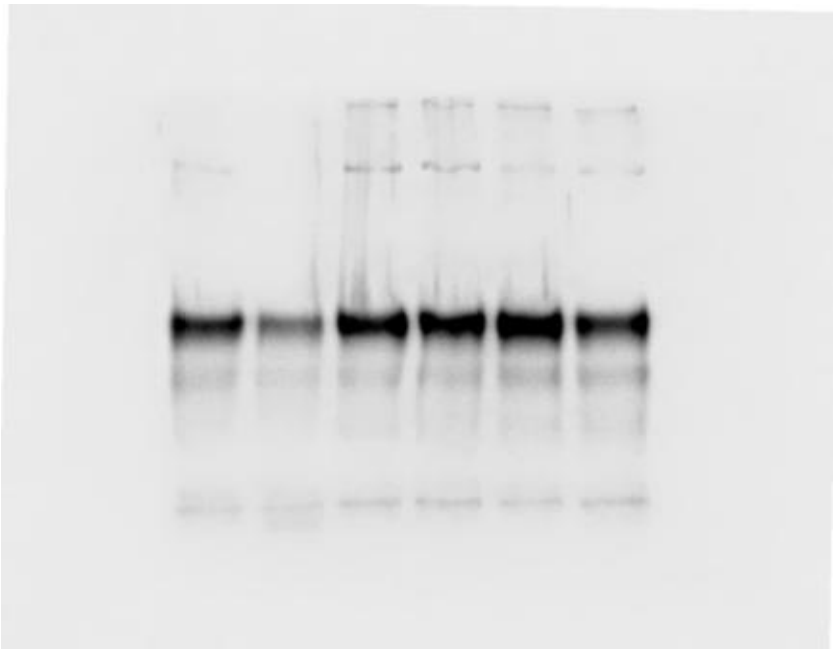

3mo iNE+iMG, pTau (AT270)

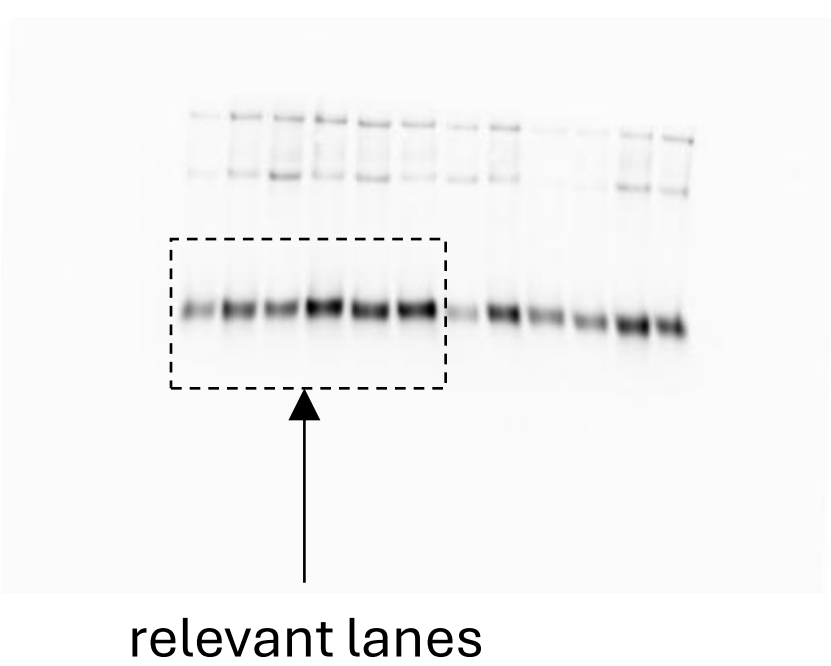

3mo iNE+iMG, total Tau

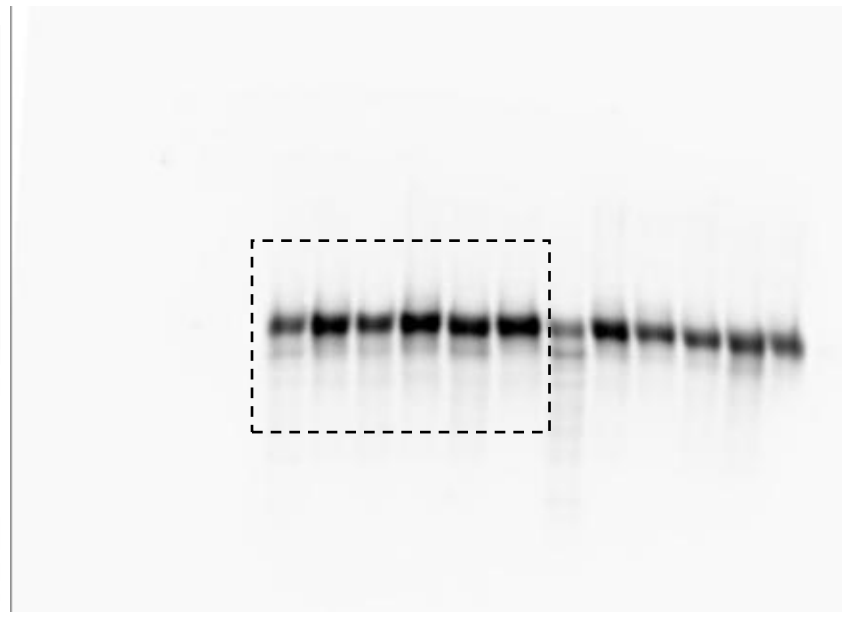

3mo iNE+iAS+iMG, pTau (AT270)

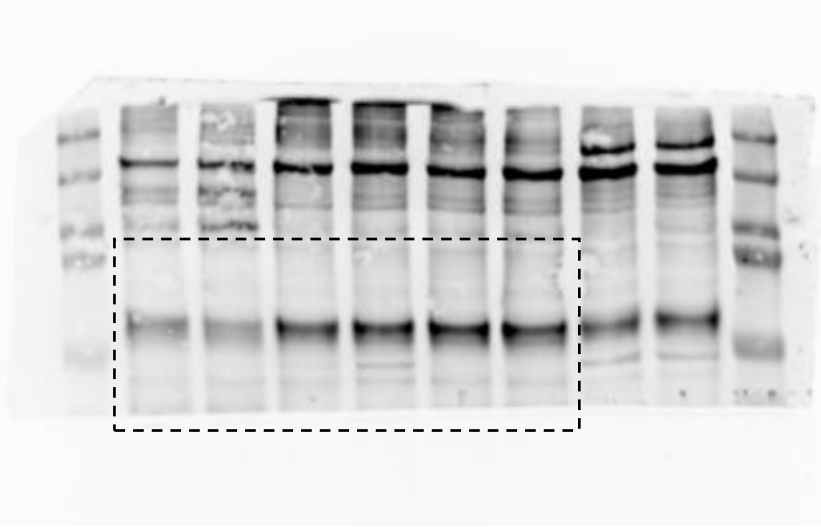

3mo iNE+iAS+iMG, total Tau

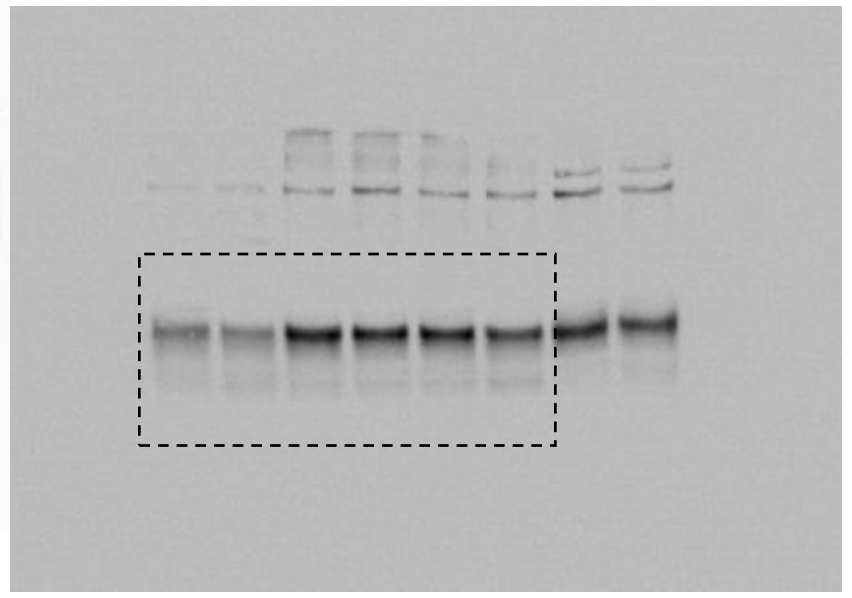

Extended Data Fig. 7O

3mo iNE+iAS+iMG, pTau (PHF1)

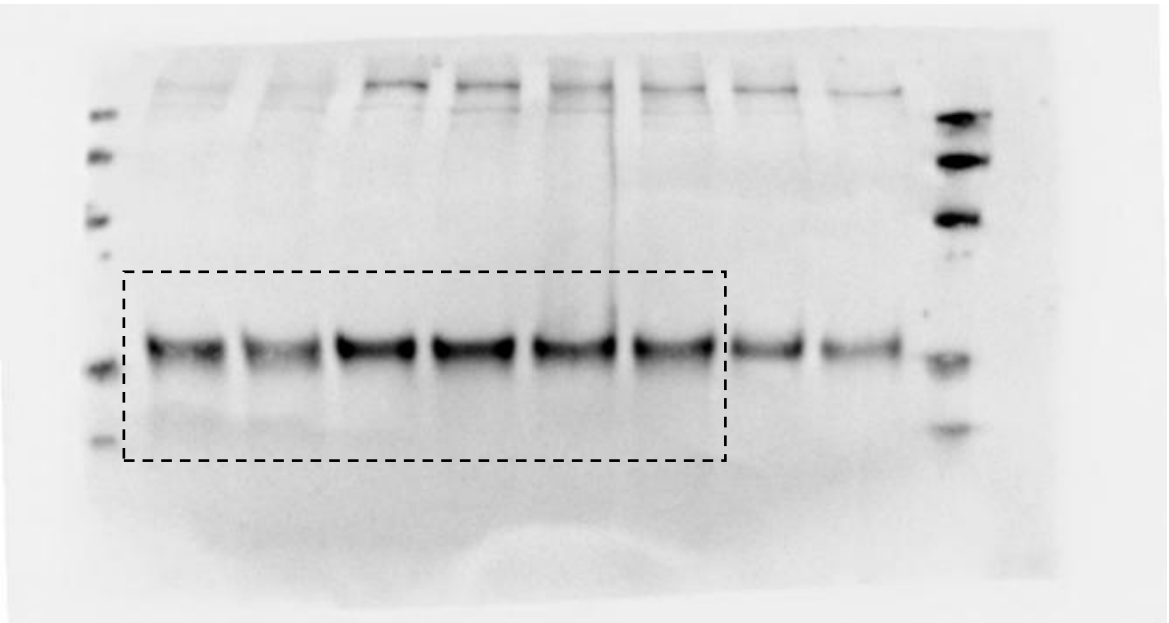

3mo iNE+iAS+iMG, total Tau

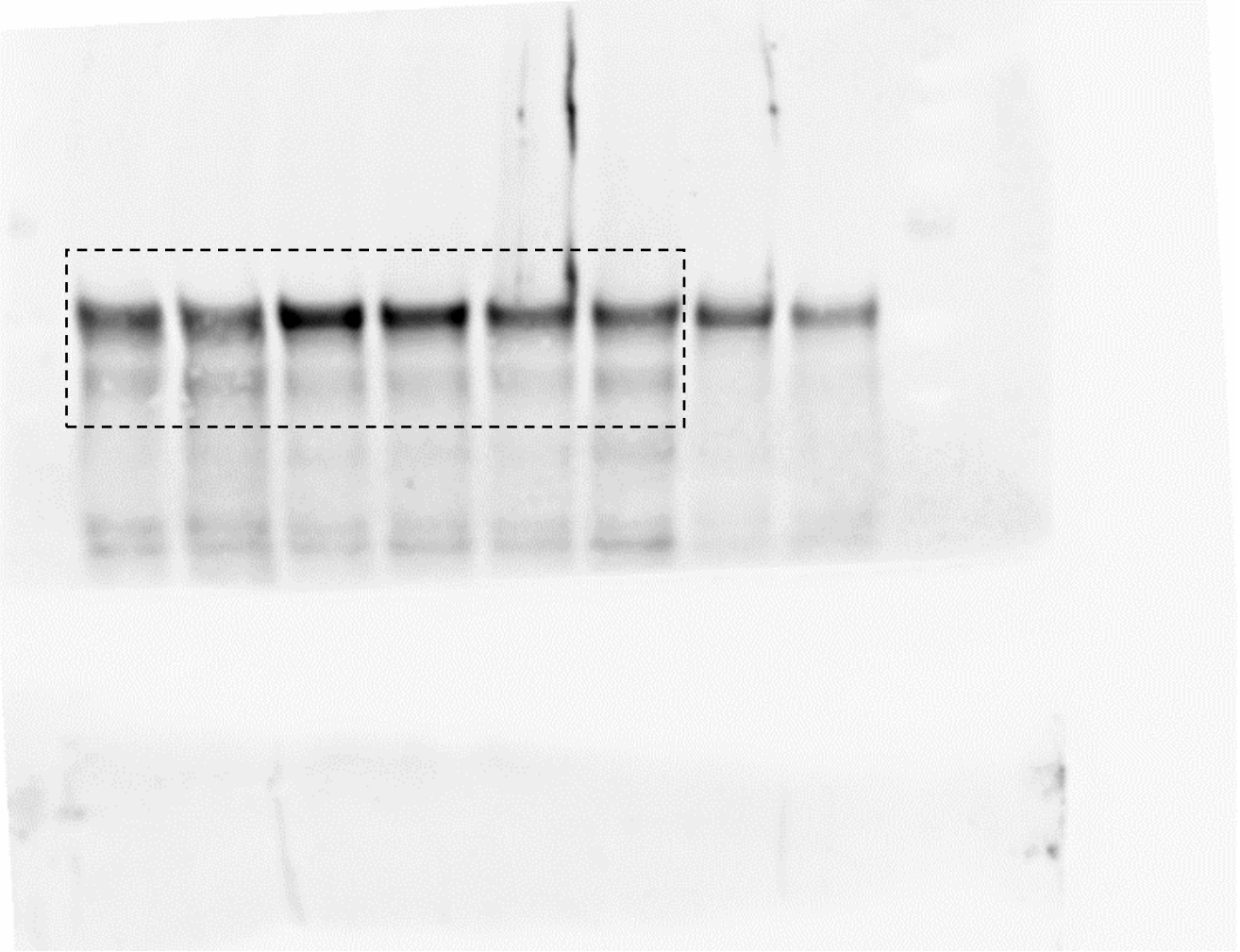

Supplement: Supplementary file 2 [file 41593_2026_2367_MOESM23_ESM.pdf]
